# ABCB1 Overexpression Is a Key Initiator of Resistance to Tyrosine Kinase Inhibitors in CML Cell Lines

**DOI:** 10.1371/journal.pone.0161470

**Published:** 2016-08-18

**Authors:** Laura N. Eadie, Timothy P. Hughes, Deborah L. White

**Affiliations:** 1 Cancer Theme, South Australian Health and Medical Research Institute (SAHMRI), Adelaide, South Australia; 2 Department of Medicine, University of Adelaide, Adelaide, South Australia; 3 Department of Paediatrics, University of Adelaide, Adelaide, South Australia; 4 Division of Haematology, SA Pathology, Adelaide, South Australia; 5 Centre for Cancer Biology, Adelaide, South Australia; B.C. Cancer Agency, CANADA

## Abstract

The tyrosine kinase inhibitor (TKI) imatinib has resulted in excellent responses in the majority of Chronic Myeloid Leukaemia (CML) patients; however, resistance is observed in 20–30% of patients. More recently, resistance to the second generation TKIs, nilotinib and dasatinib, has also been observed albeit at a lower incidence. ABCB1 has previously been implicated in TKI export and its overexpression linked to TKI resistance. In this study the dynamics of nilotinib resistance was studied in CML cell lines with particular focus on ABCB1 expression levels during development of resistance. Results revealed ABCB1 overexpression is likely an important initiator of nilotinib resistance *in vitro*. ABCB1 overexpression was also observed in cell lines as an intermediate step during development of resistance to imatinib and dasatinib *in vitro*. We conclude that ABCB1 overexpression may provide an initial platform to facilitate development of additional mechanisms for resistance to TKIs. This provides a rationale for investigating this phenomenon in patients undergoing TKI therapy.

## Introduction

Chronic myeloid leukemia (CML) is a malignant disorder of the blood stem cells characterized by the presence of the Philadelphia chromosome which gives rise to the BCR-ABL tyrosine kinase[1, 2]. Tyrosine kinase inhibitors (TKIs), such as imatinib[3], and the second generation inhibitors nilotinib[4] and dasatinib[5], act by selectively binding to the BCR-ABL kinase domain to inhibit kinase activity and subsequent downstream oncogenic signaling[6]. The advent of TKIs resulted in significantly improved treatment outcomes for the majority of patients with chronic phase(CP)-CML[7]; however, an estimated 20–30% of patients treated with first line imatinib therapy will exhibit primary or secondary resistance[8, 9]. More recently, resistance to second generation TKIs has also been observed[10, 11].

There are several overlapping modes of resistance to TKIs, which can be broadly classified as BCR-ABL dependent and independent. *BCR-ABL1* overexpression has been observed both *in vitro*[12–17] and *in vivo*[18–22] and presumably leads to an increase in the amount of active BCR-ABL kinase within the cell rendering clinically achievable concentrations of TKI ineffective. The most common mode of secondary TKI resistance observed clinically is mutation to key regions of the BCR-ABL kinase domain, which prevents TKI binding; up to 40% of resistant patients develop mutations while on TKI therapy[23–25]. Dasatinib is a dual BCR-ABL/Src family kinase (SFK) inhibitor whereas imatinib and nilotinib are not active against most SFKs[26]. Accordingly, overexpression and activation of SFKs, such as LYN, are often observed in imatinib and nilotinib resistant cells, both *in vitro*[27–32] and *in vivo*[28, 30, 32, 33].

Intracellular drug concentrations are dependent on numerous parameters including absorption, distribution, metabolism, excretion and toxicity (ADME-Tox). One of the major groups of transporters involved in ADME-Tox is the ATP-Binding Cassette (ABC) superfamily. The interaction of ABCB1 and ABCG2 with TKIs has been extensively reviewed with the consensus indicating that ABCB1 is involved in TKI export at clinically relevant concentrations[34]. Overexpression of ABCB1 has also been implicated in resistance to imatinib, nilotinib and dasatinib *in vitro*[15, 30, 35–39].

With the emerging interest in treating CML patients with customized therapeutic regimes, to reduce TKI resistance and maximize treatment outcomes, there will be a need for implementation of screening procedures in order to identify patients most at risk of developing resistance, thus allowing preventative clinical intervention. However, in order to determine which patients are more likely to develop TKI resistance it is necessary to understand the kinetics of resistance emergence. Generation of TKI-resistant cell lines via *in vitro* exposure to gradually increasing concentrations of imatinib[13–16, 28, 29, 36, 39–41], nilotinib[12, 30, 41] and dasatinib[15, 40] provides an experimental system resembling the *in vivo* situation. However, in the majority of previous studies, resistance mechanisms were determined only once cells demonstrated overt resistance to the end-point concentration of TKI[12, 13, 16, 28–30, 36, 39, 41, 42]; researchers did not generally examine modes of resistance in the intermediary cells created during incremental increases of drug. Those studies that did examine the intermediate stages of resistance focused on the generation of imatinib and dasatinib resistance only[14, 15, 35, 40]. Thus, in this study, two *BCR-ABL1*-expressing cell lines were cultured in increasing concentrations of nilotinib with samples examined at every stage of resistance generation, allowing interrogation of resistance development dynamics.

In an *in vitro* model of imatinib resistance, ABCB1 overexpression appeared most relevant during early stages of resistance development[40]. Thus, we postulated that increased ABCB1, leading to reduced intracellular levels of TKI, created a favorable environment for development of additional resistance mechanisms. In addition to the two nilotinib resistant cell lines generated as part of the current study, we also had access to imatinib- and dasatinib-resistant cells previously generated in our laboratory. In this prior study, kinase domain mutations and *BCR-ABL* overexpression were identified as end-point resistance mechanisms, however, ABCB1 expression was not examined over the course of resistance generation[15]. In the current study we now investigate the kinetics of ABCB1 expression and thus are able to evaluate the interplay of ABCB1 expression and secondary resistance mechanisms *in vitro* across three FDA approved TKIs. Additionally, *in vivo* evidence from two separate studies suggest that ABCB1 overexpression may also precede mutation development, disease progression and treatment failure in patients undergoing imatinib therapy[43–45]. Taken together, these results support ABCB1 overexpression as a likely initiator of resistance to imatinib, nilotinib and dasatinib and provide a strong rationale for investigations into the use of ABCB1 as a prognostic biomarker for identification of patients likely to respond poorly to TKI therapy.

## Materials and Methods

### Cell lines

*BCR-ABL1*-expressing cell lines K562 (American Type Culture Collection (ATCC) Manassas, VA, USA) and the ABCB1 overexpressing variant, K562-Dox, (Leonie Ashman, University of Newcastle, Callaghan, NSW) were cultured as described previously[46]. K562-Dox cells stably overexpress ABCB1 as a result of long-term exposure of the parental K562 cell line to the ABCB1 substrate doxorubicin.

### Generation of TKI-resistant cell lines

Cell lines maintained in liquid culture were gradually exposed to escalating concentrations of nilotinib (Tasigna; kindly provided by Novartis Pharmaceuticals, Basel, Switzerland) as described in Supplemental methods. In washout experiments, cells were washed 3× in drug free media, left to equilibrate overnight, washed 3× in drug free media and cells lysed. Control cell lines cultured in 0.1% DMSO were maintained in parallel. Imatinib and dasatinib resistant cells were generated and cultured as described previously[15].

### IC50 assays and western blotting

2×10^5^ *BCR-ABL1*-expressing cells were incubated for 2 h at 37°C/5%CO_2_ with concentrations of nilotinib ranging 0–100 μM, imatinib ranging 0–100 μM and dasatinib ranging 0–5000 nM. Following incubation, cells were lysed in Laemmli’s buffer[47] before resolution by 12% SDS-PAGE and electrophoretic transfer to PVDF membrane (GE Healthcare, Buckinghamshire, UK) at 65 mA overnight. Western blotting for phosphorylated CT10 regulator of kinase like (p-CRKL) was performed as previously described[48]. IC50 values were determined as the dose of drug required to reduce p-CRKL levels by 50% and are presented as mean ± SEM. Cyclosporine A (Sigma-Aldrich, St. Louis, MO, USA) is an inhibitor of ABCB1 and was used at 10 μM; PSC-833 is a cyclosporine A derivative kindly provided by Novartis Pharmaceuticals and was used at 10 μM. Western blotting for other proteins was performed as described in Supplemental methods.

### Cell viability assays

Cells were washed and resuspended in fresh culture media before culture in 24-well plates (Thermo Fisher Scientific, Waltham, MA, USA) in the presence of TKI at a density of 2×10^5^ cells/mL. Plates were seeded with 1 mL of cell suspension in triplicate, placed in sterilised containers and incubated for 72 h before cell viability determination with 7-aminoactinomycin D (7-AAD; Invitrogen Life Technologies, Carlsbad, CA, USA) and Phycoerythrin (PE)-conjugated Annexin V antibody (BD Biosciences, Franklin Lakes, NJ, USA). Flow cytometric analysis was conducted with an FC500 flow cytometer (Beckman Coulter, Miami, FL, USA) and FCS Express 4 software (De Novo Software, Los Angeles, CA, USA).

### Flow cytometry and fluorescent substrate efflux studies

ABCB1 cell surface expression and function was measured as described previously[46]. Mean Fluorescence Intensity was used to calculate fold changes in ABCB1 expression in resistant vs. control cells.

### Real time quantitative polymerase chain reaction (RQ-PCR)

1×10^7^ intermediately resistant cells were stored in TRIzol stabilization solution (Invitrogen Life Technologies) at -80°C. RNA was extracted using the phenol/chloroform method[49] and cDNA synthesized using random hexamers (GeneWorks, Hindmarsh, SA, Australia) and Superscript II reverse transcriptase (Invitrogen Life Technologies). Primers were designed using Primer Express software v2.0 (Applied Biosystems, Foster City, CA, USA), and sequences were as described in Supplemental methods. Amplification was performed using RT^2^ real-time SYBR Green/ROX PCR Master Mix (SuperArray Bioscience, Frederick, MD, USA) on a RotorGene real time PCR machine (Corbett Research, San Francisco, CA, USA). Results were analysed using Rotor-Gene 6000 Series software (Corbett Research) and the relative expression levels of the genes of interest were calculated by the comparative Ct method using the 2^ΔΔCt^ formula to achieve results for relative quantification. Control cell lines were used as calibrators and all samples were normalized to house keeping genes (*BCR* for *ABCB1*, *GUSB* for *LYN*).

### *BCR-ABL1* quantitation and mutation analysis

*BCR-ABL1* mRNA expression levels were quantitated using the TaqMan Universal PCR Master Mix (Applied Biosystems) and the ABI Prism 7500 Sequence Detection System (Applied Biosystems) as described previously[15]. *BCR-ABL1* kinase domain sequencing was performed as described previously[15].

### Statistics

Statistical tests were performed using the GraphPad Prism 5 statistical software (GraphPad Prism Inc, La Jolla, CA, USA). Normality tests were performed on each data set using the D’Agostino & Pearson omnibus normality test. The Mann-Whitney Rank Sum or the Student’s *t*-test were used to determine differences between experimental groups depending on whether the data sets failed or passed the normality test, respectively. Differences were considered to be statistically significant when the probability value (*p*-value) was <0.05.

## Results

### Generation of nilotinib resistant cells lines

**Fig 1:**
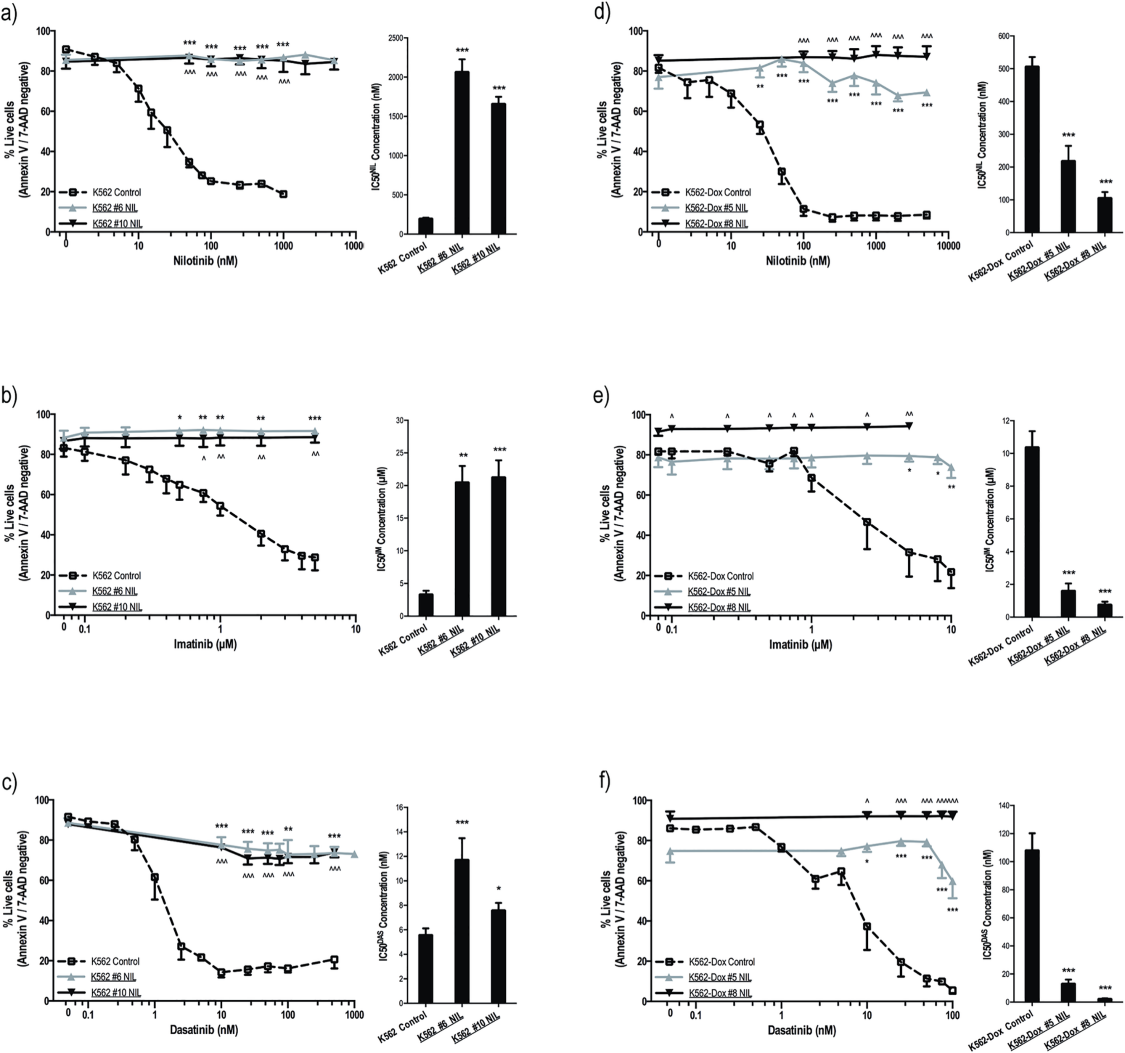
K562 and K562-Dox cells cultured long term in nilotinib demonstrate cross-resistance to imatinib and dasatinib. (**a-c**) K562 and (**d-f**) K562-Dox cells were incubated for 3 days with the indicated concentrations of (**a,d**) nilotinib, (**b,e**) imatinib or (**c,f**) dasatinib. Cell viability was determined by Annexin V/7-aminoactinomycin D staining in at least three independent experiments and expressed as %live cells (line graphs). p-CRKL dependent IC50 (dose of TKI required to reduce p-CRKL levels by 50%) was calculated by western blot in at least three independent experiments. Average IC50 values of the corresponding densitometry analyses are denoted in the column graphs. Representative western blots are shown in S1 Fig. Cell viability statistical analyses compared % live cells at common TKI concentrations; western blot statistical analyses compared resistant cells to corresponding controls. Analyses were performed using unpaired Student’s *t*-test (Welch’s correction was applied for data groups with unequal SD) or Mann-Whitney Rank Sum test. Statistically significant *p*-values are denoted by carets (^) or asterisks (* *p*<0.05; ** *p*<0.01; *** *p*<0.001). Error bars represent SEM. NIL = nilotinib.

Nilotinib resistant K562 and K562-Dox (ABCB1 overexpressing) cells were generated; nilotinib concentrations up to 100 nM were easily tolerated, however, upon exposure to 125 nM nilotinib, cell growth stalled and viability significantly decreased (S1 Table). After prolonged exposure to 125 nM nilotinib (K562 nilotinib intermediate #6), cell viability increased to ~80% and resistance to nilotinib was evaluated by IC50 based on p-CRKL protein expression. These assays rely on BCR-ABL kinase inhibition as the key readout and thus enable a distinction between BCR-ABL *dependent* and BCR-ABL *independent* resistance; an increase in IC50 value indicates *on target*, BCR-ABL dependent resistance. K562 #6 NIL cells demonstrated significantly increased IC50^NIL^ when compared with control cells (2066 nM vs. 199 nM, *p*<0.001; Fig 1A, S1A Fig). Resistance was confirmed by cell viability assays (87% vs. 19% survival in 1000 nM nilotinib, *p*<0.001; Fig 1A). K562 #6 NIL cells were assessed for resistance to imatinib and dasatinib and again demonstrated significantly increased IC50 when compared with control cells (20.5 μM vs. 3.3 μM, *p* = 0.004 and 11.7 nM vs. 5.6 nM, *p*<0.001 for imatinib and dasatinib respectively; Fig 1B and 1C, S1B and S1C Fig). These results were confirmed by cell viability assays (92% vs. 29% in 5 μM imatinib and 74% vs. 21% in 500 nM dasatinib, *p*<0.001; Fig 1B and 1C).

In order to replicate the *in vivo* concentration of nilotinib to which a patient’s leukemic cells are exposed[50], K562 #6 NIL cells were cultured in increasing concentration of nilotinib for a further two months until a concentration of 2 μM nilotinib was reached. Once this concentration was tolerated, the resultant K562 #10 NIL cells were assessed for maintenance of resistance compared with control cells (IC50^NIL^ = 1661 nM; 85% survival in 1000 nM nilotinib, *p*<0.001 for all nilotinib concentrations; Fig 1A). K562 #10 NIL cells also maintained resistance to imatinib (IC50^IM^ = 21.2 μM, 89% survival in 5 μM imatinib, *p*<0.001; Fig 1B) and dasatinib (IC50^DAS^ = 7.6 nM, *p* = 0.038; 74% survival in 500 nM dasatinib, *p*<0.001; Fig 1C). Thus the generation of nilotinib resistance in K562 led to a pan-resistant phenotype in all cases.

Development of nilotinib resistance was also evaluated in the setting of ABCB1 overexpression (S1 Table): K562-Dox #5 NIL cells demonstrated significantly increased survival compared with control cells (74% vs. 8% survival in 1000 nM nilotinib, *p*<0.001; Fig 1D). Similar results were observed for imatinib (79% vs. 31% survival in 5 μM imatinib, *p* = 0.014; Fig 1E) and dasatinib (60% vs. 5% survival in 100 nM dasatinib, *p*<0.001; Fig 1F). Analogous levels of resistance were observed for K562-Dox #8 NIL cells (Fig 1D–1F).

However, when resistance to TKI was assessed by p-CRKL dependent IC50, unexpected results were observed: IC50 to all three TKIs decreased. IC50^NIL^ decreased from 507 nM to 219 nM (*p*<0.001; Fig 1D, S1D Fig); IC50^IM^ decreased from 10.4 μM to 1.62 μM (*p*<0.001; Fig 1E, S1E Fig); IC50^DAS^ decreased from 108 nM to 13.1 nM (p<0.001; Fig 1F, S1F Fig). A further decrease in IC50 was observed in K562-Dox #8 NIL cells despite the obvious resistance to all three TKIs measured in cell viability assays (Fig 1D–1F, S1D, S1E and S1F Fig). Given that p-CRKL is a downstream target of BCR-ABL, a decrease in IC50 indicated that these cells had developed a BCR-ABL independent mechanism of resistance.

### ABCB1 overexpression is the initiator of resistance to nilotinib *in vitro*

**Fig 2:**
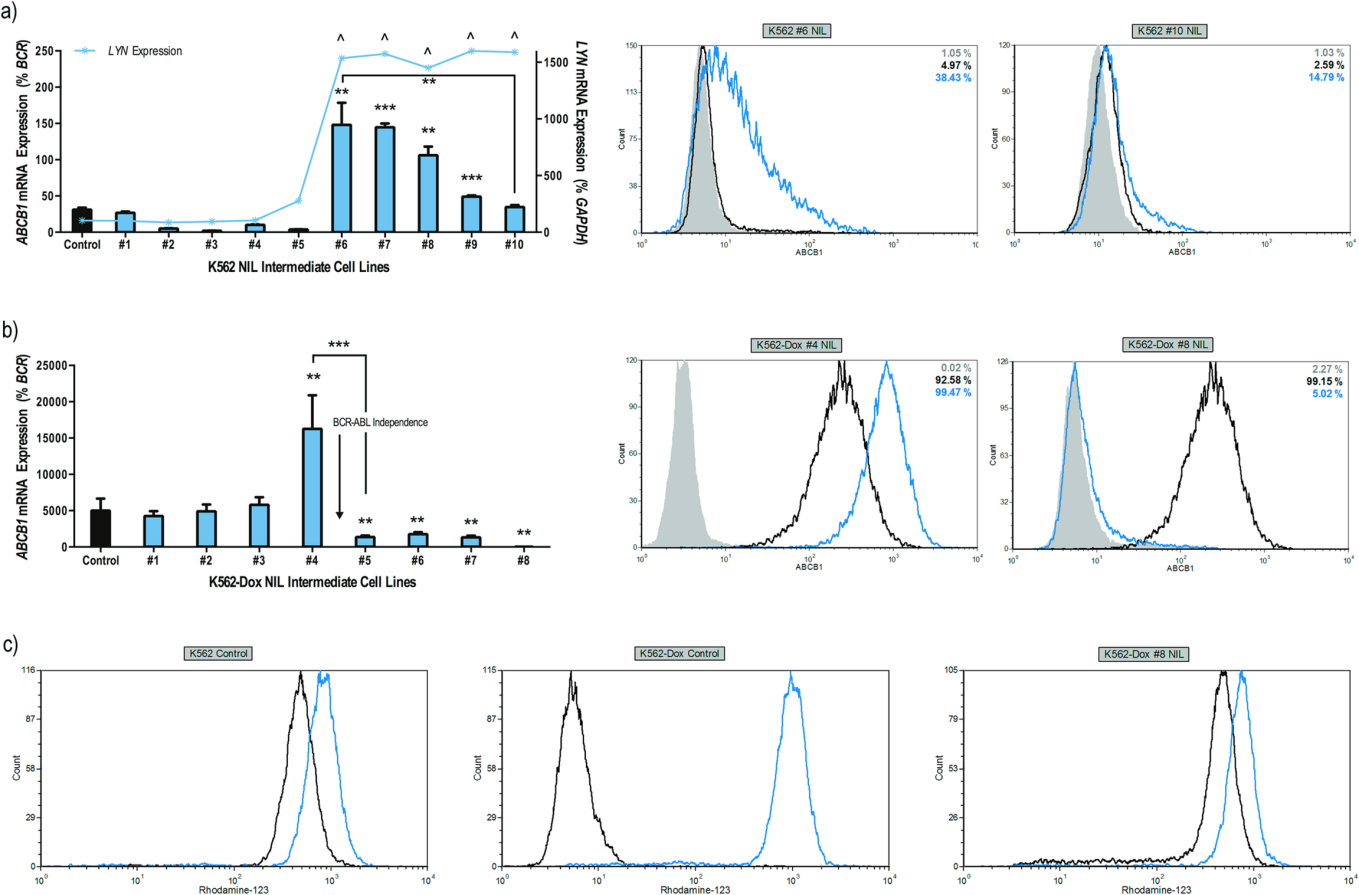
Onset of nilotinib resistance in K562 and K562-Dox cells coincides with ABCB1 overexpression; ABCB1 levels decrease upon continued high dose nilotinib exposure. Expression levels of ABCB1 mRNA/protein were assessed in (**a**) K562 and (**b**) K562-Dox nilotinib resistant cells. K562 cells were also assessed for *LYN* mRNA expression. mRNA expression represents the mean of six independent experiments performed in triplicate. The histograms shown are representative of typical protein expression levels. The blue and black lines represent resistant and control cells respectively, the grey filled histograms represent cells stained with isotype control and percentage of cells staining positive for ABCB1 expression is indicated (**c**) K562-Dox cells were stained with the fluorescent ABCB1 substrate rhodamine-123 and assessed for their ability to effectively efflux rhodamine-123 in the absence and presence of the specific ABCB1 inhibitor PSC-833. K562 cells were used as a negative control for ABCB1 efflux. The histograms shown are representative of typical efflux patterns. The black lines represent fluorescent substrate alone while the blue lines represent PSC-833 mediated inhibition of ABCB1. Statistical analyses compared mRNA levels (as a percentage of house keeping genes) in each resistant intermediate to control cells. Analyses were performed using unpaired Student’s *t*-test (Welch’s correction was applied for data groups with unequal SD) or Mann-Whitney Rank Sum test. Statistically significant *p*-values are denoted by carets (^) or asterisks (* *p*<0.05; ** *p*<0.01; *** *p*<0.001). Error bars represent SEM. NIL = nilotinib.

Once overt TKI resistance had been established, potential mechanisms of resistance were interrogated. Due to the likelihood of ABCB1 involvement in nilotinib transport[34], ABCB1 protein and mRNA expression was monitored over the course of resistance development. The onset of resistance within K562 #6 NIL cells coincided with a significant increase in *ABCB1* mRNA expression: resistant cells demonstrated up to 4.7-fold greater levels of *ABCB1* mRNA compared with control cells (*p*<0.01; Fig 2A). Surface ABCB1 protein expression was also significantly increased in K562 #6 NIL cells compared with control (up to 56% ABCB1 vs. 8%; *p* = 0.002). However, upon continued culture to 2 μM nilotinib (intermediate #10), both ABCB1 mRNA and protein expression reduced to levels comparable with that of control cells (Fig 2A).

**Fig 3:**
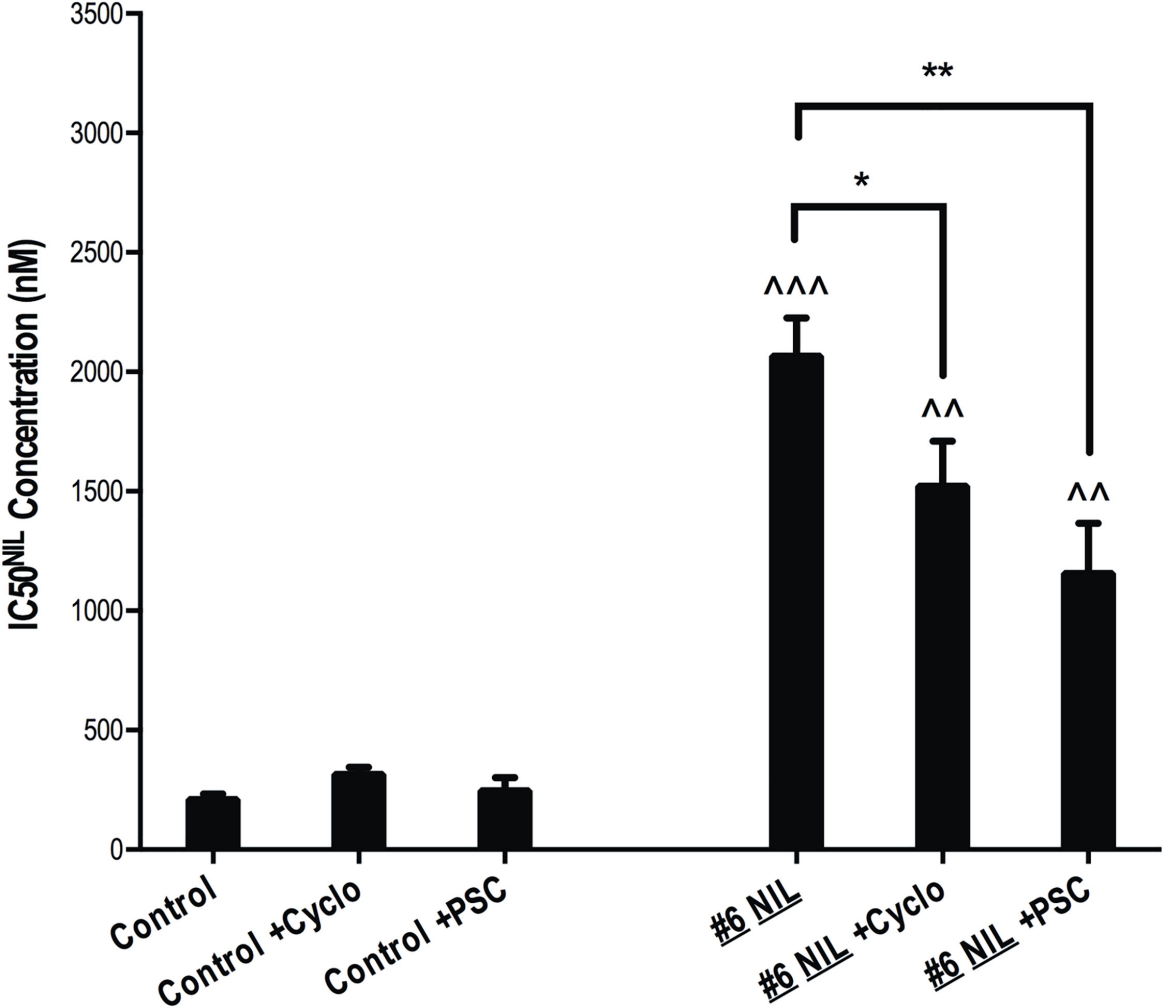
Inhibition of ABCB1 significantly reduces resistance in ABCB1 overexpressing K562 cells. p-CRKL dependent IC50 (dose of TKI required to reduce p-CRKL levels by 50%) was calculated by western blot in at least four independent experiments with the average IC50 values from the resultant densitometry analyses denoted by the columns. Statistical analyses compared resistant cells in the presence vs. absence of ABCB1 inhibition (asterisks) and also resistant cells in the absence and presence of ABCB1 inhibitors with control cells in the absence of inhibitor (carets). Analyses were performed using unpaired Student’s *t*-test (Welch’s correction was applied for data groups with unequal SD) or Mann-Whitney Rank Sum test. Statistically significant *p*-values are denoted by carets (^) or asterisks (* *p*<0.05; ** *p*<0.01; *** *p*<0.001). Error bars represent SEM. NIL = nilotinib; cyclo = cyclosporine; PSC = PSC-833.

In order to determine whether increased expression of ABCB1 had a direct impact on nilotinib resistance, p-Crkl dependent IC50^NIL^ experiments were performed in the presence and absence of two ABCB1 inhibitors (cyclosporine and PSC-833). Indeed, a significant reduction in IC50^NIL^ was observed in resistant cells in the presence of both inhibitors: K562 #6 NIL IC50^NIL^ reduced from 2066 nM to 1522 nM in the presence of cyclosporine (*p* = 0.043) and to 1155 nM in the presence of PSC-833 (*p* = 0.009). No significant effect of ABCB1 inhibition on IC50^NIL^ was observed in K562 control cells (Fig 3). Interestingly, even in the presence of complete ABCB1 inhibition, the IC50^NIL^ of K562 #6 NIL cells remained significantly increased compared with K562 control cells indicating the presence of an additional resistance mechanism in this cell line. Further evidence for the direct involvement of ABCB1 overexpression in nilotinib resistance was provided when IC50^NIL^ was examined over a seven-day period in K562 #6 NIL cells overexpressing ABCB1. During this time period, ABCB1 expression decreased from 85% to 72% to 61% with a corresponding decrease in IC50^NIL^ from 2700 nM to 2120 nM to 1570 nM (S2 Fig). Taken together, these data indicate a key role for ABCB1 overexpression in the nilotinib resistance observed in this cell line model.

The phenomenon of initial ABCB1 overexpression followed by a significant reduction to almost negligible levels was also observed during development of TKI resistance in K562-Dox cells; an intriguing observation given that this cell line has stably expressed ABCB1 for over a decade. Initially, elevated expression of both *ABCB1* mRNA (3.5-fold increase, *p* = 0.002) and protein (MFI 487 vs. 213, *p*<0.001) was observed when compared with levels in K562-Dox control cells (Fig 2B). However, upon prolonged culture (when cell growth stalled and viability decreased, S1 Table) two populations of cells became apparent, an ABCB1-positive population and an ABCB1-negative population. Interestingly, the ABCB1-negative population increased correspondingly with time in culture (S3 Fig) suggesting that those cells with decreased ABCB1 expression had a growth advantage and were clonally selected over time. Following continued escalation of nilotinib to 2 μM (#8 NIL resistance intermediate), ABCB1 mRNA and protein expression was negligible and comparable to levels observed in parental K562 cells (Fig 2B).

### K562-Dox cells lose all functional ABCB1 following continued culture in nilotinib

To our knowledge this is the first report documenting loss of ABCB1 expression in K562-Dox cells due to continued culture in TKI. Thus, it was important to determine whether there was also loss of ABCB1 function. Therefore, K562-Dox #8 NIL cells were assessed for ability to effectively efflux the ABCB1 substrate rhodamine-123. Results demonstrated a complete loss of ABCB1 function with the resistant cell line demonstrating comparable rhodamine-123 levels to the parental K562 cell line. Rhodamine-123 levels in K562-Dox control cells were as expected (Fig 2C).

### ABCB1 overexpression precedes increased LYN phosphorylation in nilotinib resistant K562 cells

**Fig 4:**
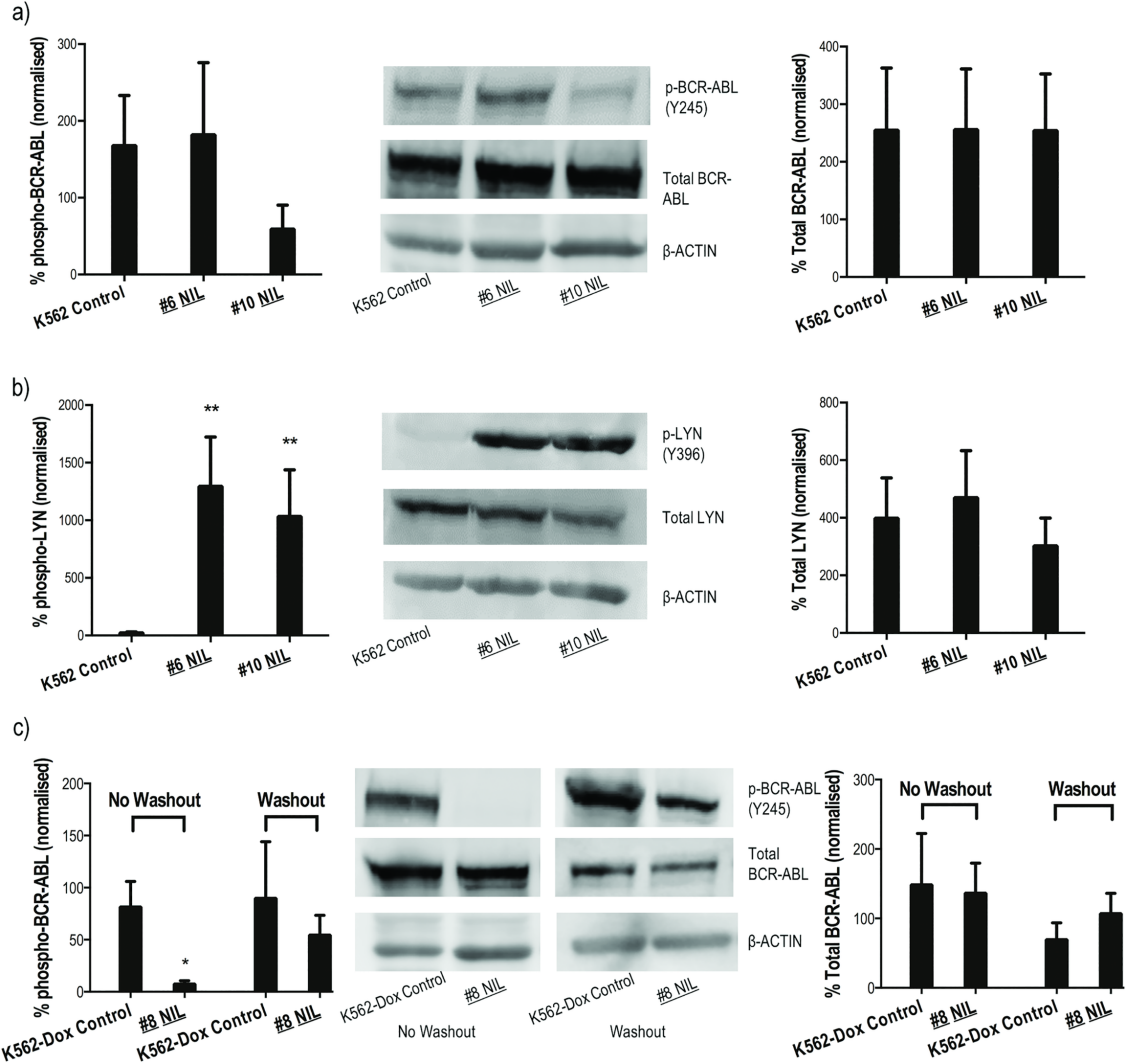
Nilotinib resistant K562 cells exhibit increased activity of LYN kinase. Expression levels of total and phospho (**a,c**) BCR-ABL and (**b**) LYN were assessed during development of nilotinib resistance in (**a,b**) K562 and (**c**) K562-Dox cells. Protein levels were normalised to β-ACTIN and levels in nilotinib resistant intermediates were then compared to levels in control cells. The western blot analyses are representative and the corresponding densitometry analyses represent the mean of at least three experiments. Statistical analyses compared resistant cells to corresponding controls. Analyses were performed using unpaired Student’s *t*-test (Welch’s correction was applied for data groups with unequal SD). Statistically significant *p*-values denoted by asterisks (* *p*<0.05; ** *p*<0.01). Error bars represent SEM. NIL = nilotinib.

Because *BCR-ABL1* overexpression is a common form of TKI resistance observed in patients[21], resistant K562 cells were screened for increased expression of BCR-ABL protein. Results demonstrated this possibility unlikely since no overexpression or increased kinase activity (as measured by phospho-BCR-ABL) was observed in early or late stage resistance intermediates (Fig 4A). No kinase domain mutations were detected in any K562 resistance intermediates (S1 Appendix). Overexpression/increased activity of LYN was also investigated: results demonstrated increased *LYN* mRNA in K562 #6 NIL cells that remained high for the duration of nilotinib escalation (15–18 fold increase compared with control cells, *p*<0.05; Fig 2A). Additionally, catalytic activation of LYN increased significantly as measured by autophosphorylation at tyrosine 396 (Y396, *p* = 0.008; Fig 4B).

### ABCB1 overexpression precedes Bcr-Abl independent resistance in nilotinib resistant K562-Dox cells

BCR-ABL kinase activity was assessed in nilotinib resistant K562-Dox cells to confirm adequate inhibition of phosphorylation. Nilotinib was removed from K562-Dox #8 NIL culture media by thorough washing followed by overnight equilibration. Total and phospho-BCR-ABL levels were determined and compared with the levels in cells that remained in continuous culture with nilotinib. Results demonstrated complete inhibition of kinase activity in the presence of nilotinib. However, upon drug washout, reactivation of BCR-ABL occurred resulting in similar levels of phospho-BCR-ABL in both resistant and control cells (*p*>0.05). Importantly, total BCR-ABL levels remained unaffected in the absence and presence of nilotinib (Fig 4C). Thus, while nilotinib was effective at inhibiting BCR-ABL kinase activity, cells demonstrated complete resistance indicating the presence of an independent resistance mechanism. Additionally, no increase in LYN expression or activity was observed (S4 Fig); in fact, resistant cells demonstrated significantly decreased levels of LYN compared with control cells excluding LYN as a likely effector of resistance as has previously been reported[30, 41].

### ABCB1 overexpression precedes resistance to imatinib and dasatinib *in vitro*

**Fig 5:**
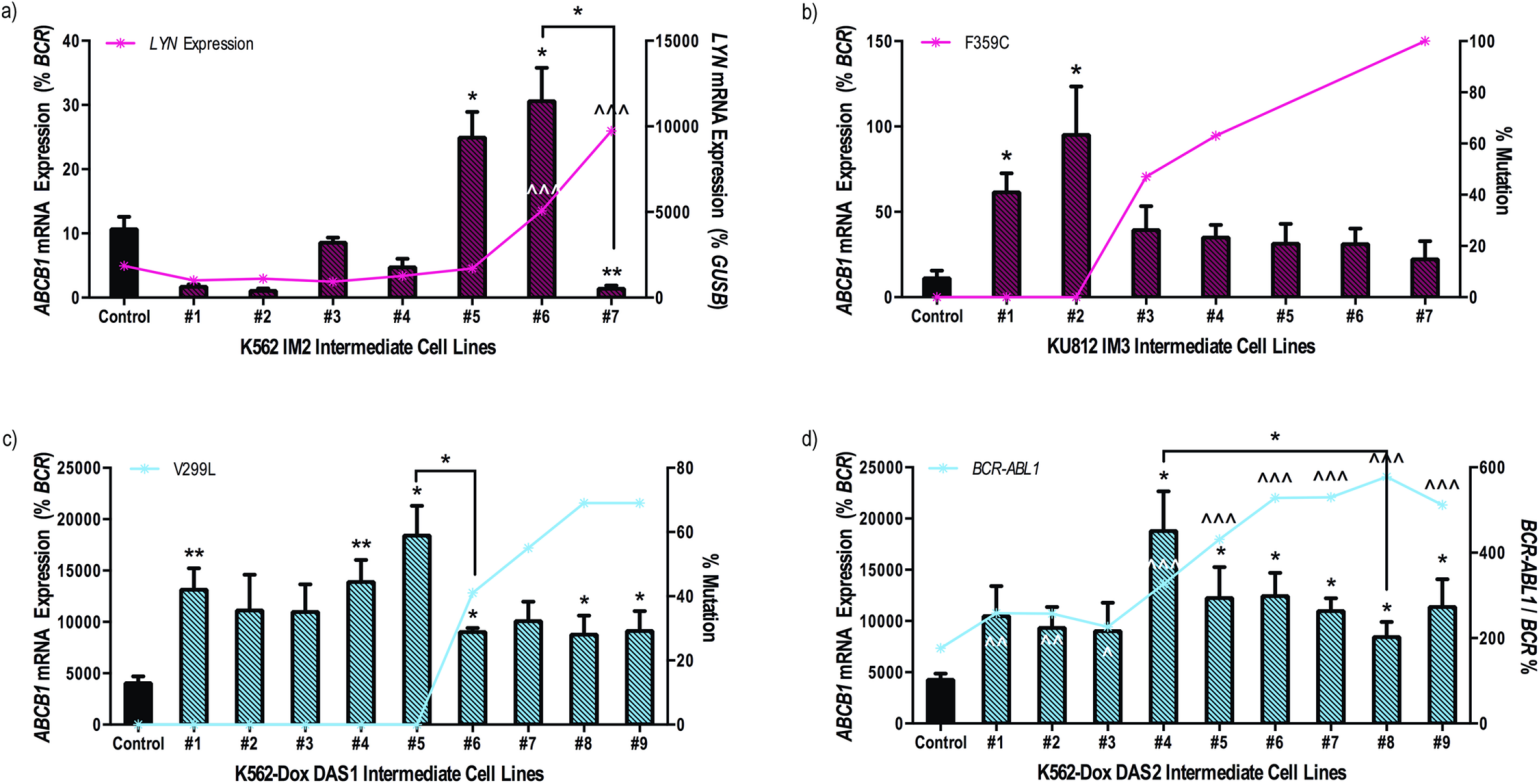
*ABCB1* mRNA levels increase initially in imatinib and dasatinib resistant cell lines but decrease following emergence of additional resistance mechanisms. Expression levels of *ABCB1* mRNA were assessed in (**a**) K562, (**b**) KU812 and (**c, d**) K562-Dox cells resistant to (**a, b**) imatinib or (**c, d**) dasatinib. Expression levels were then correlated with other, previously defined, resistance mechanisms denoted by red and blue lines[15]. Specifically, (**a**) *LYN* mRNA expression, % of (**b**) imatinib resistant (F359C) and (**c**) dasatinib resistant (V299L) kinase domain mutations, (**d**) *BCR-ABL1* mRNA expression. *ABCB1* mRNA expression represents the mean of at least four independent experiments performed in triplicate. Statistical analyses compared mRNA levels (as a percentage of house keeping genes) in each resistant intermediate to corresponding control cells. Analyses were performed using unpaired Student’s *t*-test (Welch’s correction was applied for data groups with unequal SD) or Mann-Whitney Rank Sum test. Statistically significant *p*-values are denoted by carets (^) or asterisks (* *p*<0.05; ** *p*<0.01). Error bars represent SEM. IM = imatinib; DAS = dasatinib.

Given that ABCB1 is likely to play a role in the transport of, and resistance to, imatinib and dasatinib[34], ABCB1 expression was examined in previously established imatinib and dasatinib resistant cell lines and correlated with already defined mechanisms of resistance[15]. Indeed, *ABCB1* mRNA expression increased initially in response to both imatinib and dasatinib exposure, and then decreased once further resistance mechanisms predominated. When compared with control cells, imatinib-resistant K562 cells demonstrated up to 2.8-fold greater levels of *ABCB1* mRNA; a significant decrease in expression of *ABCB1* mRNA was associated with an increase in *LYN* mRNA expression (Fig 5A). Similarly, a peak of 8.6-fold greater *ABCB1* mRNA levels in KU812 imatinib resistant cells was observed prior to a concomitant decrease in expression following development of the imatinib resistant F359C kinase domain mutation (Fig 5B). The same initial increase in *ABCB1* mRNA followed by a decrease once additional resistance mechanisms predominated was observed in two separate dasatinib resistant K562-Dox cell lines (DAS1 and DAS2; Fig 5C and 5D). ABCB1 protein expression confirmed these data (S5 Fig). Taken together, these results indicate that ABCB1 overexpression likely acts as an initial mediator of resistance to nilotinib, imatinib and dasatinib providing favourable conditions for further resistance development.

## Discussion

Nilotinib-resistant cell lines have previously been generated as a means of recapitulating *in vivo* resistance, however, this is the first study in which the resistance mechanisms were studied in a sequential fashion to better define the dynamics of resistance development. The model described here allowed an interrogation of the interplay between various mechanisms in the development of frank resistance. In addition, previous studies have generated cells resistant to 20 nM[30] and 50 nM[12] nilotinib whereas the end-point concentration of nilotinib chosen in this study (2 μM) better reflects the trough plasma level observed in patients[50] and thus is a more accurate representation of the nilotinib concentration to which a patient’s leukaemic cells are exposed.

Even though TKIs such as imatinib, and more recently nilotinib and dasatinib, have resulted in significantly better treatment outcomes for the majority of patients with CML, resistance to therapy is still a problem for a significant proportion of patients (up to 30%)[8, 9]. Additionally, BCR-ABL independent mechanisms of resistance are poorly understood. Accordingly, a greater understanding of the kinetics of resistance development, especially in the BCR-ABL independent setting, is required. As we move into the era of customised treatment regimes, it becomes important to predict which patients are likely to respond to a given TKI such that treatment plans are tailored to the individual. Thus, *in vitro* cell line models are key in determining, not only common modes of TKI resistance, but also kinetics of resistance development. In this study, two cell line models of nilotinib resistance were generated and resistance emergence examined; resistance kinetics in response to imatinib and dasatinib were assessed in four additional cell lines. Results highlighted ABCB1 overexpression as a likely initiator of resistance to all three TKIs.

Notably, this is the first report of ABCB1 overexpression in the K562 cell line in response to exposure to nilotinib, which decreased following a concordant increase in LYN kinase activity. LYN overexpression has previously been reported as a BCR-ABL independent mode of resistance[27]. However, in the current study, K562 #10 NIL cells with increased LYN activity also demonstrated increased p-CRKL dependent IC50, which was unexpected if the resistance was BCR-ABL-independent. This, combined with the fact that LYN reportedly regulates BCR-ABL activity[33, 51, 52], suggests that LYN overexpression is in fact a BCR-ABL dependent mechanism of resistance, which challenges current dogma. More intriguing is the fact that these cells remain resistant to dasatinib, which has activity against Src Family Kinases including Lyn. This raises the possibility that an additional resistance mechanism/s is present (eg: overexpression of an efflux transporter, activation of an alternate signalling pathway) and warrants further investigation.

Exposure of K562-Dox cells to nilotinib resulted in a complete loss of ABCB1 expression and function following initial ABCB1 overexpression. K562-Dox cells have stably expressed ABCB1 for over a decade and this is the first report of complete loss of ABCB1 expression back to levels observed in parental K562 cells. No overexpression or increased activity of LYN was observed. Instead, resistance is likely attributable to a novel BCR-ABL independent mechanism of resistance, an area of research under current investigation.

Examination of imatinib- and dasatinib-resistant cell lines generated within our laboratory and characterised previously[15] also highlighted ABCB1 overexpression as an initiating mechanism of resistance to these TKIs. Again, most previous studies examined resistance in cells surviving end-point TKI concentration only[12, 13, 16, 28–30, 36, 39, 41, 42]. Two studies in which intermediately resistant cells were studied either failed to examine ABCB1 expression[14], or did not correlate the increased ABCB1 expression observed with TKI resistance[35]. One recent study, however, did confirm our findings with results demonstrating exposure of K562 cells lines to imatinib and dasatinib resulted in increased *ABCB1* mRNA. Authors also hypothesised that increases in transporter expression may be important in the early stages of resistance development but not as crucial after prolonged exposure to TKI[40]. The involvement of ABCB1 in development of resistance to nilotinib, imatinib and dasatinib is further substantiated when the rate of resistance acquisition in K562-Dox cells, which already overexpress ABCB1, is considered. When compared with K562 and KU812 cells (which express negligible basal levels of ABCB1) K562-Dox cells develop resistance to nilotinib, imatinib and dasatinib at a faster rate, withstanding higher concentrations of TKIs earlier in the dose escalation process. K562-Dox cells also required less time to tolerate a given concentration of TKI, reaching end point concentrations faster. These data confirm ABCB1 overexpression as a key initiator of resistance to all three TKIs (S2 Table). Thus, it appears likely that ABCB1 overexpression causes increased export of TKIs leading to lower intracellular concentrations. This then results in suboptimal BCR-ABL inhibition, which presumably creates a favourable environment for development of other more efficient resistance mechanisms. Given the variety of resistance mechanisms observed, both in our studies and those from others, it is likely that the events following ABCB1 up regulation, are stochastic in nature and dependent on the cell lines studied.

In conclusion, the findings detailed here suggest that ABCB1 overexpression likely provides an initial platform required for development of additional mechanisms of resistance to TKIs *in vitro*. The cell line models of resistance described provide a valuable tool for studying resistance in patients. We observed a consistent upregulation of ABCB1 as a result of exposure to three FDA approved TKIs; we are currently investigating whether this phenomenon also occurs in patients receiving TKI therapy. With further *in vivo* validation, the results presented here suggest monitoring of ABCB1 levels may potentially identify patients likely to develop additional resistance and/or respond poorly to their current therapy regime.

## Supporting Information

S1 Appendix K562 nilotinib resistance intermediates do not harbour any Bcr-Abl kinase domain mutations. DNA sequencing of the Bcr-Abl kinase domain of K562 control cells and nilotinib 4resistance intermediates was conducted and compared with the GenBank ABL reference sequence. Data demonstrate that neither the control cells nor any of the resistance intermediates contain mutations. Data were analysed using Mutation Surveyor Version 3.24 with each peak representing a DNA base (A = Adenine; C = Cytosine; G = Guanine; or T = Thymine) in the 5’-3’ direction of the ABL sequence. The base number for both the reference sequence and the sequences of interest are indicated. The amino acid and corresponding residue number are also indicated. The amino acid sequences exactly match the reference sequence with no kinase domain mutations present. NIL = nilotinib.
(PDF)

S1 Fig K562 and K562-Dox cells cultured long term in nilotinib demonstrate cross-resistance to imatinib and dasatinib. (**a-c**) K562 and (**d-f**) K562-Dox cells were incubated with the indicated concentrations of (**a,d**) nilotinib, (**b,e**) imatinib or (**c,f**) dasatinib. CRKL western blotting was performed to determine the concentration of TKI required for 50% BCR-ABL kinase inhibition. The western blot analyses are representative and the arrows indicate approximate IC50. NIL = nilotinib; IM = imatinib; DAS = dasatinib.
(TIF)

S2 Fig ABCB1 expression levels directly influence IC50^NIL^ in nilotinib resistant K562 cells. p-CRKL dependent IC50 (dose of TKI required to reduce p-CRKL levels by 50%) was determined three separate times over a period of seven days; ABCB1 expression was simultaneously determined. The western blot analyses shown represent a single experiment with the ImageQuant densitometry analyses depicted underneath. The boxes around the 1500 nM nilotinib western bands highlight the clear difference in %p-CRKL likely attributable to the level of ABCB1 expression. The percentages displayed in the histograms denote cells positive for ABCB1 expression. The bold blue and black lines represent resistant and control cells respectively, stained with ABCB1 antibody while the grey filled histograms represent cells stained with isotype control antibody.
(TIF)

S3 Fig Two populations of K562-Dox cells (ABCB1 positive and ABCB1 negative) arise following prolonged culture in nilotinib. Expression levels of ABCB1 protein were assessed in K562-Dox #5 NIL cells over a period of two months compared with that in control cells. The histograms shown are representative of typical expression levels. The blue and black lines represent resistant and control cells respectively, the grey filled histograms represent cells stained with isotype control.
(TIF)

S4 Fig There is no increase in LYN expression or activity in K562-Dox cells suggesting BCR-ABL independent resistance to nilotinib. (**a**) mRNA and (**b**) protein expression levels for LYN kinase were assessed during development of nilotinib resistance in K562-Dox cells. mRNA expression represents the mean of at least three independent experiments performed in triplicate. Western blot analyses shown are representative with the corresponding quantitation representing the mean of three experiments. mRNA levels were normalised to *GUSB*, protein levels were normalised to β-ACTIN. Statistical analyses were performed using unpaired Student’s t-test with statistically significant *p*-values denoted by asterisks (** *p*<0.01; *** *p*<0.001). Error bars represent SEM. NIL = nilotinib.
(TIF)

S5 Fig ABCB1 protein levels increase initially in imatinib and dasatinib resistant cell lines but decrease following emergence of predominating resistance mechanisms. Expression levels of ABCB1 protein were assessed in (**a**) K562 (**b**) KU812 (**c, d**) K562-Dox cells cultured in increasing concentrations of (**a,b**) imatinib and (**c,d**) dasatinib and compared with levels in corresponding control cells. The histograms shown are representative of typical expression levels with the MFI indicated. The blue and black lines represent resistant and control cells respectively, the grey filled histograms represent cells stained with isotype control. IM = imatinib; DAS = dasatinib.
(TIF)

S6 Fig *ABCB1* mRNA levels increase initially in imatinib resistant KU812 cells then decrease following emergence of kinase domain mutations. Expression levels of *ABCB1* mRNA were assessed in KU812 cells resistant to imatinib. Expression levels were then correlated with other, previously defined, resistance mechanisms[15]. Specifically, % of *BCR-ABL1* mRNA (maroon line) and % of various kinase domain mutations (orange, yellow, green, blue, purple lines) are indicated. mRNA expression represents the mean of at least three independent experiments performed in triplicate. Error bars represent SEM. IM = imatinib.
(TIF)

S1 Table Summary of nilotinib (NIL) concentrations to which cell line resistance intermediates were exposed and the corresponding number of days before dose was increased. (DOCX)

S2 Table Summary of imatinib (IM) and dasatinib (DAS) concentrations to which cell line resistance intermediates were exposed and the corresponding number of days before dose was increased. Cells lines shown in bold have been assessed for ABCB1 expression in the current manuscript. Note that cell lines expressing negligible levels of ABCB1 (K562 and KU812) required longer periods of time to develop resistance to IM and DAS compared with K562-Dox cells which demonstrate overexpression of ABCB1 initially. Additionally, generation of a DAS resistant K562 cell line was extremely difficult (cells kept dying at the 1 nM DAS stage, intermediate #2) and was attempted three times before successful dose escalation occurred. A DAS resistant KU812 cell line could not be generated due to the inherent sensitivity to TKIs of this cell line.
(DOCX)

## References

[1] Nowell PC, Hungerford DA. A Minute Chromosome in Human Chronic Granulocytic Leukemia. Science. 1960;132:1497.

[2] Rowley JD. Letter: A new consistent chromosomal abnormality in chronic myelogenous leukaemia identified by quinacrine fluorescence and Giemsa staining. Nature. 1973;243(5405):290–3. Epub 1973/06/01. . 4126434 10.1038/243290a0

[3] Druker BJ, Tamura S, Buchdunger E, Ohno S, Segal GM, Fanning S, et al Effects of a selective inhibitor of the Abl tyrosine kinase on the growth of Bcr-Abl positive cells. Nat Med. 1996;2(5):561–6. Epub 1996/05/01. . 8616716 10.1038/nm0596-561

[4] Manley PW, Breitenstein W, Bruggen J, Cowan-Jacob SW, Furet P, Mestan J, et al Urea derivatives of STI571 as inhibitors of Bcr-Abl and PDGFR kinases. Bioorg Med Chem Lett. 2004;14(23):5793–7. Epub 2004/10/27. doi: S0960-894X(04)01164-3 [pii] 10.1016/j.bmcl.2004.09.042 . 15501042

[5] Lombardo LJ, Lee FY, Chen P, Norris D, Barrish JC, Behnia K, et al Discovery of N-(2-chloro-6-methyl- phenyl)-2-(6-(4-(2-hydroxyethyl)- piperazin-1-yl)-2-methylpyrimidin-4- ylamino)thiazole-5-carboxamide (BMS-354825), a dual Src/Abl kinase inhibitor with potent antitumor activity in preclinical assays. J Med Chem. 2004;47(27):6658–61. Epub 2004/12/24. 10.1021/jm049486a . 15615512

[6] Goldman JM, Melo JV. Chronic myeloid leukemia—advances in biology and new approaches to treatment. N Engl J Med. 2003;349(15):1451–64. Epub 2003/10/10. 10.1056/NEJMra020777 . 14534339

[7] Hughes TP, Kaeda J, Branford S, Rudzki Z, Hochhaus A, Hensley ML, et al Frequency of major molecular responses to imatinib or interferon alfa plus cytarabine in newly diagnosed chronic myeloid leukemia. N Engl J Med. 2003;349(15):1423–32. Epub 2003/10/10. 10.1056/NEJMoa030513 349/15/1423 [pii]. . 14534335

[8] Giles FJ, le Coutre PD, Pinilla-Ibarz J, Larson RA, Gattermann N, Ottmann OG, et al Nilotinib in imatinib-resistant or imatinib-intolerant patients with chronic myeloid leukemia in chronic phase: 48-month follow-up results of a phase II study. Leukemia. 2013;27(1):107–12. 10.1038/leu.2012.181 . 22763385

[9] Khorashad JS, Kelley TW, Szankasi P, Mason CC, Soverini S, Adrian LT, et al BCR-ABL1 compound mutations in tyrosine kinase inhibitor-resistant CML: frequency and clonal relationships. Blood. 2013;121(3):489–98. 10.1182/blood-2012-05-431379 23223358 PMC3548169

[10] Hochhaus A, Saglio G, Larson RA, Kim DW, Etienne G, Rosti G, et al Nilotinib is associated with a reduced incidence of BCR-ABL mutations vs imatinib in patients with newly diagnosed chronic myeloid leukemia in chronic phase. Blood. 2013;121(18):3703–8. 10.1182/blood-2012-04-423418 . 23502220 PMC4915803

[11] Kantarjian H, Shah NP, Hochhaus A, Cortes J, Shah S, Ayala M, et al Dasatinib versus imatinib in newly diagnosed chronic-phase chronic myeloid leukemia. N Engl J Med. 2010;362(24):2260–70. Epub 2010/06/08. doi: NEJMoa1002315 [pii] 10.1056/NEJMoa1002315 . 20525995

[12] Camgoz A, Gencer EB, Ural AU, Baran Y. Mechanisms responsible for nilotinib resistance in human chronic myeloid leukemia cells and reversal of resistance. Leuk Lymphoma. 2013;54(6):1279–87. 10.3109/10428194.2012.737919 . 23098068

[13] le Coutre P, Tassi E, Varella-Garcia M, Barni R, Mologni L, Cabrita G, et al Induction of resistance to the Abelson inhibitor STI571 in human leukemic cells through gene amplification. Blood. 2000;95(5):1758–66. Epub 2000/02/26. . 10688835

[14] Scappini B, Gatto S, Onida F, Ricci C, Divoky V, Wierda WG, et al Changes associated with the development of resistance to imatinib (STI571) in two leukemia cell lines expressing p210 Bcr/Abl protein. Cancer. 2004;100(7):1459–71. 10.1002/cncr.20131 . 15042680

[15] Tang C, Schafranek L, Watkins DB, Parker WT, Moore S, Prime JA, et al Tyrosine kinase inhibitor resistance in chronic myeloid leukemia cell lines: investigating resistance pathways. Leuk Lymphoma. 2011;52(11):2139–47. Epub 2011/07/02. 10.3109/10428194.2011.591013 . 21718141

[16] Weisberg E, Griffin JD. Mechanism of resistance to the ABL tyrosine kinase inhibitor STI571 in BCR/ABL-transformed hematopoietic cell lines. Blood. 2000;95(11):3498–505. . 10828035

[17] Zhao F, Mancuso A, Bui TV, Tong X, Gruber JJ, Swider CR, et al Imatinib resistance associated with BCR-ABL upregulation is dependent on HIF-1alpha-induced metabolic reprograming. Oncogene. 2010;29(20):2962–72. Epub 2010/03/17. doi: onc201067 [pii] 10.1038/onc.2010.67 20228846 PMC2874611

[18] Barnes DJ, Palaiologou D, Panousopoulou E, Schultheis B, Yong AS, Wong A, et al Bcr-Abl expression levels determine the rate of development of resistance to imatinib mesylate in chronic myeloid leukemia. Cancer Res. 2005;65(19):8912–9. Epub 2005/10/06. doi: 65/19/8912 [pii] 10.1158/0008-5472.CAN-05-0076 . 16204063

[19] Campbell LJ, Patsouris C, Rayeroux KC, Somana K, Januszewicz EH, Szer J. BCR/ABL amplification in chronic myelocytic leukemia blast crisis following imatinib mesylate administration. Cancer Genet Cytogenet. 2002;139(1):30–3. Epub 2003/01/28. doi: S0165460802006155 [pii]. . 12547154 10.1016/s0165-4608(02)00615-5

[20] De Braekeleer E, Douet-Guilbert N, Le Bris MJ, Morel F, De Braekeleer M. Translocation 3;21, trisomy 8, and duplication of the Philadelphia chromosome: a rare but recurrent cytogenetic pathway in the blastic phase of chronic myeloid leukemia. Cancer Genet Cytogenet. 2007;179(2):159–61. Epub 2007/11/27. 10.1016/j.cancergencyto.2007.09.003 . 18036406

[21] Gorre ME, Mohammed M, Ellwood K, Hsu N, Paquette R, Rao PN, et al Clinical resistance to STI-571 cancer therapy caused by BCR-ABL gene mutation or amplification. Science. 2001;293(5531):876–80. Epub 2001/06/26. 10.1126/science.1062538 1062538 [pii]. . 11423618

[22] Hochhaus A, Kreil S, Corbin AS, La Rosee P, Muller MC, Lahaye T, et al Molecular and chromosomal mechanisms of resistance to imatinib (STI571) therapy. Leukemia. 2002;16(11):2190–6. Epub 2002/10/26. 10.1038/sj.leu.2402741 . 12399961

[23] Branford S, Rudzki Z, Walsh S, Parkinson I, Grigg A, Szer J, et al Detection of BCR-ABL mutations in patients with CML treated with imatinib is virtually always accompanied by clinical resistance, and mutations in the ATP phosphate-binding loop (P-loop) are associated with a poor prognosis. Blood. 2003;102(1):276–83. Epub 2003/03/08. 10.1182/blood-2002-09-2896 2002-09-2896 [pii]. . 12623848

[24] Hughes T, Saglio G, Branford S, Soverini S, Kim DW, Muller MC, et al Impact of baseline BCR-ABL mutations on response to nilotinib in patients with chronic myeloid leukemia in chronic phase. J Clin Oncol. 2009;27(25):4204–10. Epub 2009/08/05. doi: JCO.2009.21.8230 [pii] 10.1200/JCO.2009.21.8230 . 19652056 PMC4979230

[25] Muller MC, Cortes JE, Kim DW, Druker BJ, Erben P, Pasquini R, et al Dasatinib treatment of chronic-phase chronic myeloid leukemia: analysis of responses according to preexisting BCR-ABL mutations. Blood. 2009;114(24):4944–53. Epub 2009/09/26. 10.1182/blood-2009-04-214221 . 19779040 PMC4916940

[26] Hantschel O, Rix U, Superti-Furga G. Target spectrum of the BCR-ABL inhibitors imatinib, nilotinib and dasatinib. Leuk Lymphoma. 2008;49(4):615–9. Epub 2008/04/10. doi: 791364886 [pii] 10.1080/10428190801896103 . 18398720

[27] Dai Y, Rahmani M, Corey SJ, Dent P, Grant S. A Bcr/Abl-independent, Lyn-dependent form of imatinib mesylate (STI-571) resistance is associated with altered expression of Bcl-2. J Biol Chem. 2004;279(33):34227–39. Epub 2004/06/04. 10.1074/jbc.M402290200 M402290200 [pii]. . 15175350

[28] Donato NJ, Wu JY, Stapley J, Gallick G, Lin H, Arlinghaus R, et al BCR-ABL independence and LYN kinase overexpression in chronic myelogenous leukemia cells selected for resistance to STI571. Blood. 2003;101(2):690–8. Epub 2003/01/02. 10.1182/blood.V101.2.690 101/2/690 [pii]. . 12509383

[29] Grosso S, Puissant A, Dufies M, Colosetti P, Jacquel A, Lebrigand K, et al Gene expression profiling of imatinib and PD166326-resistant CML cell lines identifies Fyn as a gene associated with resistance to BCR-ABL inhibitors. Mol Cancer Ther. 2009;8(7):1924–33. Epub 2009/07/02. doi: 1535-7163.MCT-09-0168 [pii] 10.1158/1535-7163.MCT-09-0168 . 19567819

[30] Mahon FX, Hayette S, Lagarde V, Belloc F, Turcq B, Nicolini F, et al Evidence that resistance to nilotinib may be due to BCR-ABL, Pgp, or Src kinase overexpression. Cancer Res. 2008;68(23):9809–16. Epub 2008/12/03. doi: 68/23/9809 [pii] 10.1158/0008-5472.CAN-08-1008 . 19047160

[31] Okabe S, Tauchi T, Tanaka Y, Ohyashiki K. Dasatinib preferentially induces apoptosis by inhibiting Lyn kinase in nilotinib-resistant chronic myeloid leukemia cell line. J Hematol Oncol. 2011;4(1):32. Epub 2011/08/03. doi: 1756-8722-4-32 [pii] 10.1186/1756-8722-4-32 21806844 PMC3163636

[32] Wu J, Meng F, Kong LY, Peng Z, Ying Y, Bornmann WG, et al Association between imatinib-resistant BCR-ABL mutation-negative leukemia and persistent activation of LYN kinase. J Natl Cancer Inst. 2008;100(13):926–39. Epub 2008/06/26. doi: djn188 [pii] 10.1093/jnci/djn188 18577747 PMC2902818

[33] Wu J, Meng F, Lu H, Kong L, Bornmann W, Peng Z, et al Lyn regulates BCR-ABL and Gab2 tyrosine phosphorylation and c-Cbl protein stability in imatinib-resistant chronic myelogenous leukemia cells. Blood. 2008;111(7):3821–9. Epub 2008/02/01. doi: blood-2007-08-109330 [pii] 10.1182/blood-2007-08-109330 18235045 PMC2275035

[34] Eadie LN, Hughes TP, White DL. Interaction of the efflux transporters ABCB1 and ABCG2 with imatinib, nilotinib, and dasatinib. Clin Pharmacol Ther. 2014;95(3):294–306. 10.1038/clpt.2013.208 . 24107928

[35] Burger H, van Tol H, Brok M, Wiemer EA, de Bruijn EA, Guetens G, et al Chronic imatinib mesylate exposure leads to reduced intracellular drug accumulation by induction of the ABCG2 (BCRP) and ABCB1 (MDR1) drug transport pumps. Cancer Biol Ther. 2005;4(7):747–52. Epub 2005/06/23. doi: 1826 [pii]. . 15970668 10.4161/cbt.4.7.1826

[36] Hirayama C, Watanabe H, Nakashima R, Nanbu T, Hamada A, Kuniyasu A, et al Constitutive overexpression of P-glycoprotein, rather than breast cancer resistance protein or organic cation transporter 1, contributes to acquisition of imatinib-resistance in K562 cells. Pharm Res. 2008;25(4):827–35. Epub 2007/10/16. 10.1007/s11095-007-9376-3 . 17934801

[37] Illmer T, Schaich M, Platzbecker U, Freiberg-Richter J, Oelschlagel U, von Bonin M, et al P-glycoprotein-mediated drug efflux is a resistance mechanism of chronic myelogenous leukemia cells to treatment with imatinib mesylate. Leukemia. 2004;18(3):401–8. Epub 2004/01/16. 10.1038/sj.leu.2403257 2403257 [pii]. . 14724652

[38] Mahon FX, Belloc F, Lagarde V, Chollet C, Moreau-Gaudry F, Reiffers J, et al MDR1 gene overexpression confers resistance to imatinib mesylate in leukemia cell line models. Blood. 2003;101(6):2368–73. Epub 2003/03/01. 10.1182/blood.V101.6.2368 101/6/2368 [pii]. . 12609962

[39] Mahon FX, Deininger MW, Schultheis B, Chabrol J, Reiffers J, Goldman JM, et al Selection and characterization of BCR-ABL positive cell lines with differential sensitivity to the tyrosine kinase inhibitor STI571: diverse mechanisms of resistance. Blood. 2000;96(3):1070–9. Epub 2000/07/27. . 10910924

[40] Gromicho M, Dinis J, Magalhaes M, Fernandes AR, Tavares P, Laires A, et al Development of imatinib and dasatinib resistance: dynamics of expression of drug transporters ABCB1, ABCC1, ABCG2, MVP, and SLC22A1. Leuk Lymphoma. 2011;52(10):1980–90. Epub 2011/06/15. 10.3109/10428194.2011.584005 . 21663515

[41] Okabe S, Tauchi T, Tanaka Y, Ohyashiki K. Efficacy of ponatinib against ABL tyrosine kinase inhibitor-resistant leukemia cells. Biochem Biophys Res Commun. 2013;435(3):506–11. 10.1016/j.bbrc.2013.05.022 . 23684619

[42] Yuan H, Wang Z, Gao C, Chen W, Huang Q, Yee JK, et al BCR-ABL gene expression is required for its mutations in a novel KCL-22 cell culture model for acquired resistance of chronic myelogenous leukemia. J Biol Chem. 2010;285(7):5085–96. 10.1074/jbc.M109.039206 20007699 PMC2836111

[43] Eadie LN, Hughes TP, White DL. The Clinical Significance of Early Imatinib Induced ABCB1 Overexpression in Chronic Phase CML Patients: A TIDEL II Sub-Study. Blood. 2015;126(23):348–.

[44] White D, Dang P, Venables A, Saunders V, Zrim S, Zannettino A, et al ABCB1 Overexpression May Predispose Imatinib Treated CML Patients to the Development of Abl Kinase Domain Mutations, and May Be an Important Contributor to Acquired Resistance. Blood. 2006;108(11):2144–.

[45] Eadie LN, Dang P, Saunders VA, Yeung DT, Osborn MP, Grigg AP, et al The clinical significance of ABCB1 overexpression in predicting outcome of CML patients undergoing first-line imatinib treatment. Leukemia. 2016 10.1038/leu.2016.179 27416909

[46] Eadie LN, Saunders VA, Hughes TP, White DL. Degree of kinase inhibition achieved in vitro by imatinib and nilotinib is decreased by high levels of ABCB1 but not ABCG2. Leuk Lymphoma. 2013;54(3):569–78. 10.3109/10428194.2012.715345 . 22845311

[47] Nichols GL, Raines MA, Vera JC, Lacomis L, Tempst P, Golde DW. Identification of CRKL as the constitutively phosphorylated 39-kD tyrosine phosphoprotein in chronic myelogenous leukemia cells. Blood. 1994;84(9):2912–8. . 7524758

[48] White D, Saunders V, Lyons AB, Branford S, Grigg A, To LB, et al In vitro sensitivity to imatinib-induced inhibition of ABL kinase activity is predictive of molecular response in patients with de novo CML. Blood. 2005;106(7):2520–6. Epub 2005/06/16. doi: 2005-03-1103 [pii] 10.1182/blood-2005-03-1103 . 15956284

[49] Chomczynski P, Sacchi N. Single-step method of RNA isolation by acid guanidinium thiocyanate-phenol-chloroform extraction. Anal Biochem. 1987;162(1):156–9. 10.1006/abio.1987.9999 . 2440339

[50] Tanaka C, Yin OQ, Sethuraman V, Smith T, Wang X, Grouss K, et al Clinical pharmacokinetics of the BCR-ABL tyrosine kinase inhibitor nilotinib. Clin Pharmacol Ther. 2010;87(2):197–203. Epub 2009/11/20. doi: clpt2009208 [pii] 10.1038/clpt.2009.208 . 19924121

[51] Meyn MA 3rd, Wilson MB, Abdi FA, Fahey N, Schiavone AP, Wu J, et al Src family kinases phosphorylate the Bcr-Abl SH3-SH2 region and modulate Bcr-Abl transforming activity. J Biol Chem. 2006;281(41):30907–16. Epub 2006/08/17. doi: M605902200 [pii] 10.1074/jbc.M605902200 . 16912036

[52] Warmuth M, Bergmann M, Priess A, Hauslmann K, Emmerich B, Hallek M. The Src family kinase Hck interacts with Bcr-Abl by a kinase-independent mechanism and phosphorylates the Grb2-binding site of Bcr. J Biol Chem. 1997;272(52):33260–70. Epub 1998/01/31. . 9407116 10.1074/jbc.272.52.33260

