# Supplementary material for: ABCB1 Overexpression Is a Key Initiator of Resistance to Tyrosine Kinase Inhibitors in CML Cell Lines
Source: PLoS One. 2016 Aug 18;11(8):e0161470. doi: 10.1371/journal.pone.0161470 (PMC4990177; doi:10.1371/journal.pone.0161470)
Supplement: S1 Appendix — DNA sequencing of the Bcr-Abl kinase domain of K562 control cells and nilotinib 4resistance intermediates was conducted and compared with the GenBank ABL reference sequence. Data demonstrate that neither the control cells nor any of the resistance intermediates contain mutations. Data were analysed using Mutation Surveyor Version 3.24 with each peak representing a DNA base (A = Adenine; C = Cytosine; G = Guanine; or T = Thymine) in the 5’-3’ direction of the ABL sequence. The base number for both the reference sequence and the sequences of interest are indicated. The amino acid and corresponding residue number are also indicated. The amino acid sequences exactly match the reference sequence with no kinase domain mutations present. NIL = nilotinib. (PDF) [file pone.0161470.s001.pdf]

S1 Appendix: K562 nilotinib resistance intermediates do not harbour any Bcr-Abl kinase domain mutations

DNA sequencing of the Bcr-Abl kinase domain of K562 control cells and nilotinib 4resistance intermediates was conducted and compared with the GenBank ABL reference sequence. Data demonstrate that neither the control cells nor any of the resistance intermediates contain mutations. Data were analysed using Mutation Surveyor Version 3.24 with each peak representing a DNA base (A=Adenine; C=Cytosine; G=Guanine; or T=Thymine) in the 5'-3' direction of the ABL sequence. The base number for both the reference sequence and the sequences of interest are indicated. The amino acid and corresponding residue number are also indicated. The amino acid sequences exactly match the reference sequence with no kinase domain mutations present. NIL=nilotinib

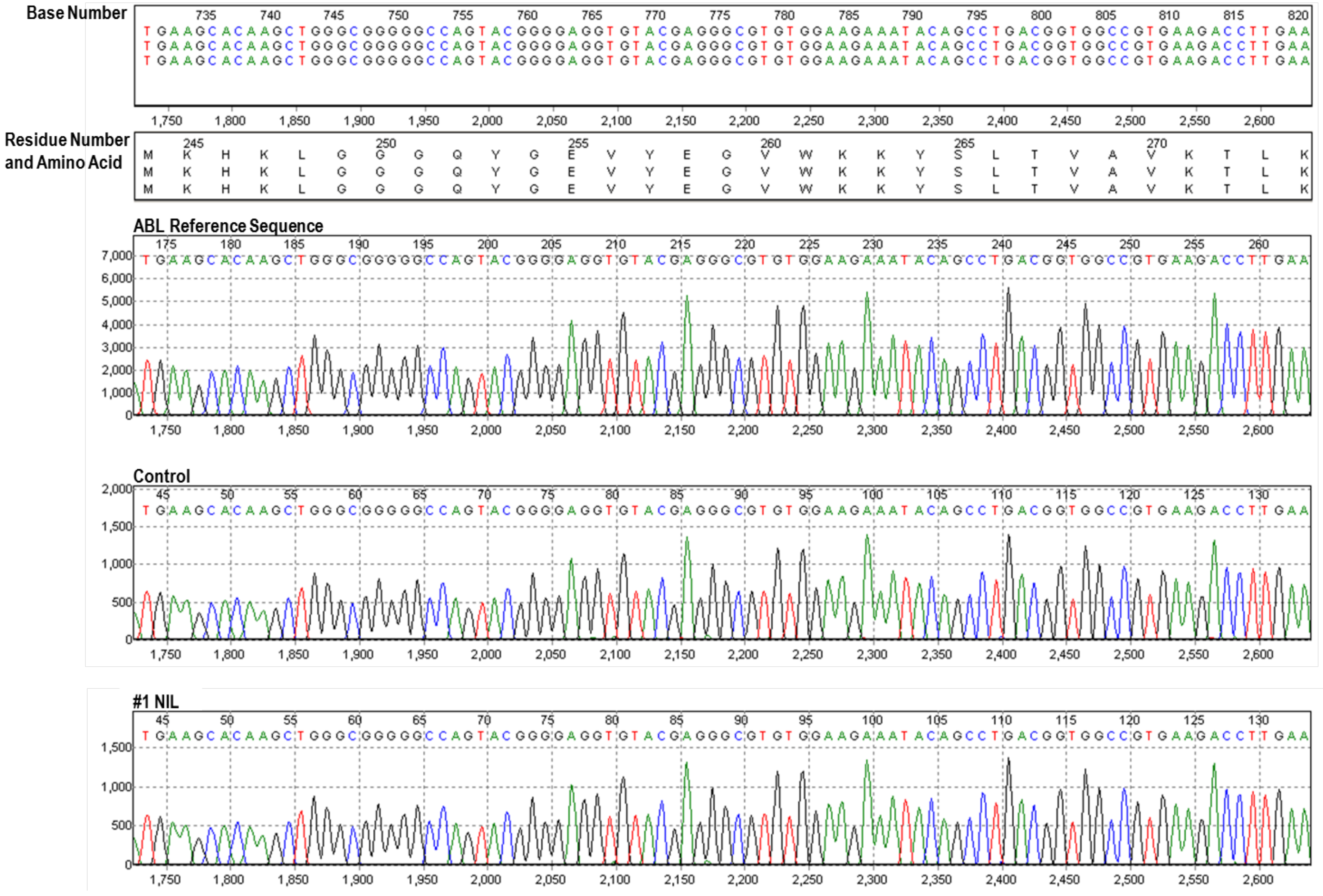
Base Number
Residue Number and Amino Acid
M K H K L G G G Q Y G E V Y E G V W K K Y S L T V A V K T L K
ABL Reference Sequence
Control
#1 NIL

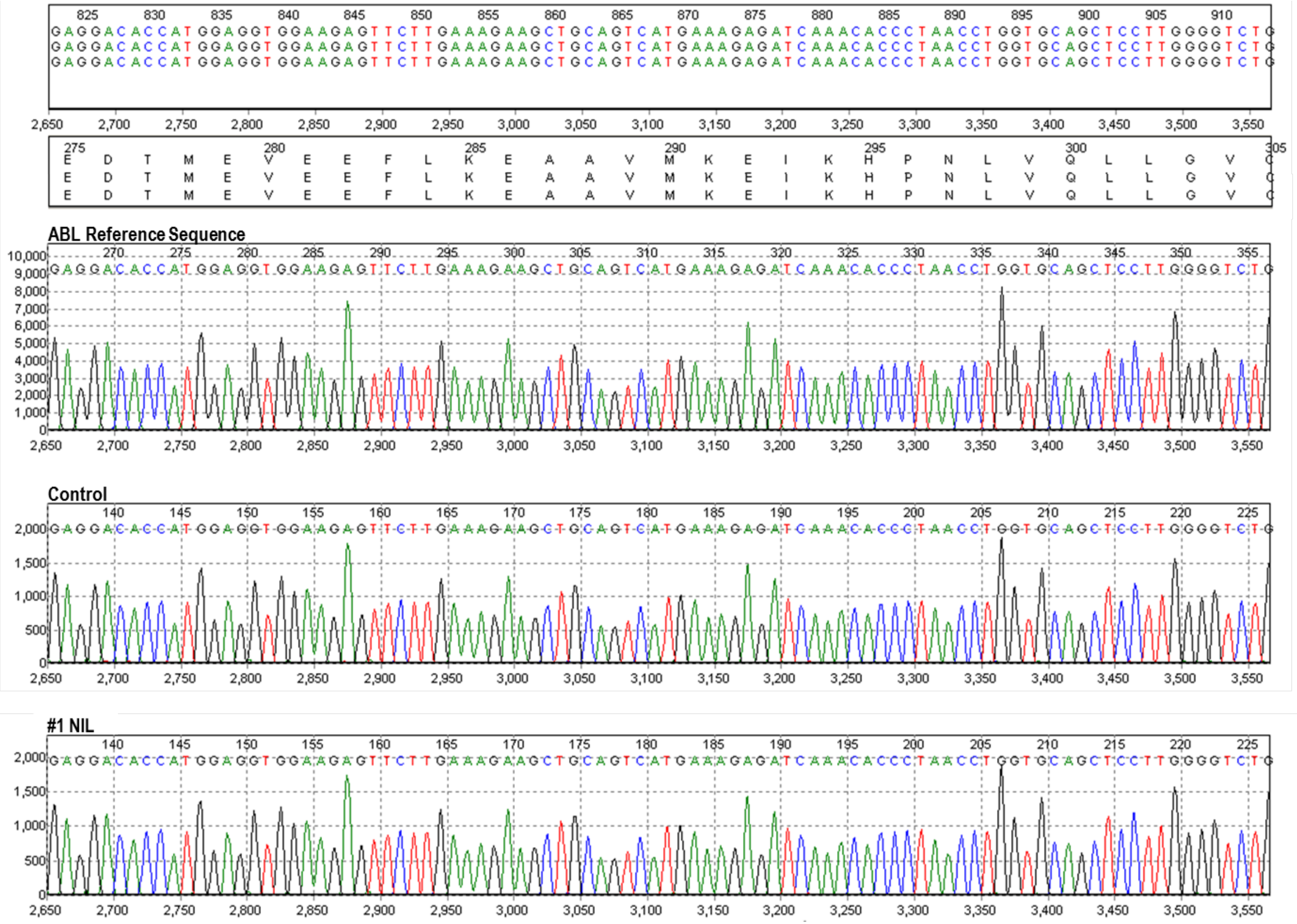
ABL Reference Sequence
Control
#1 NIL

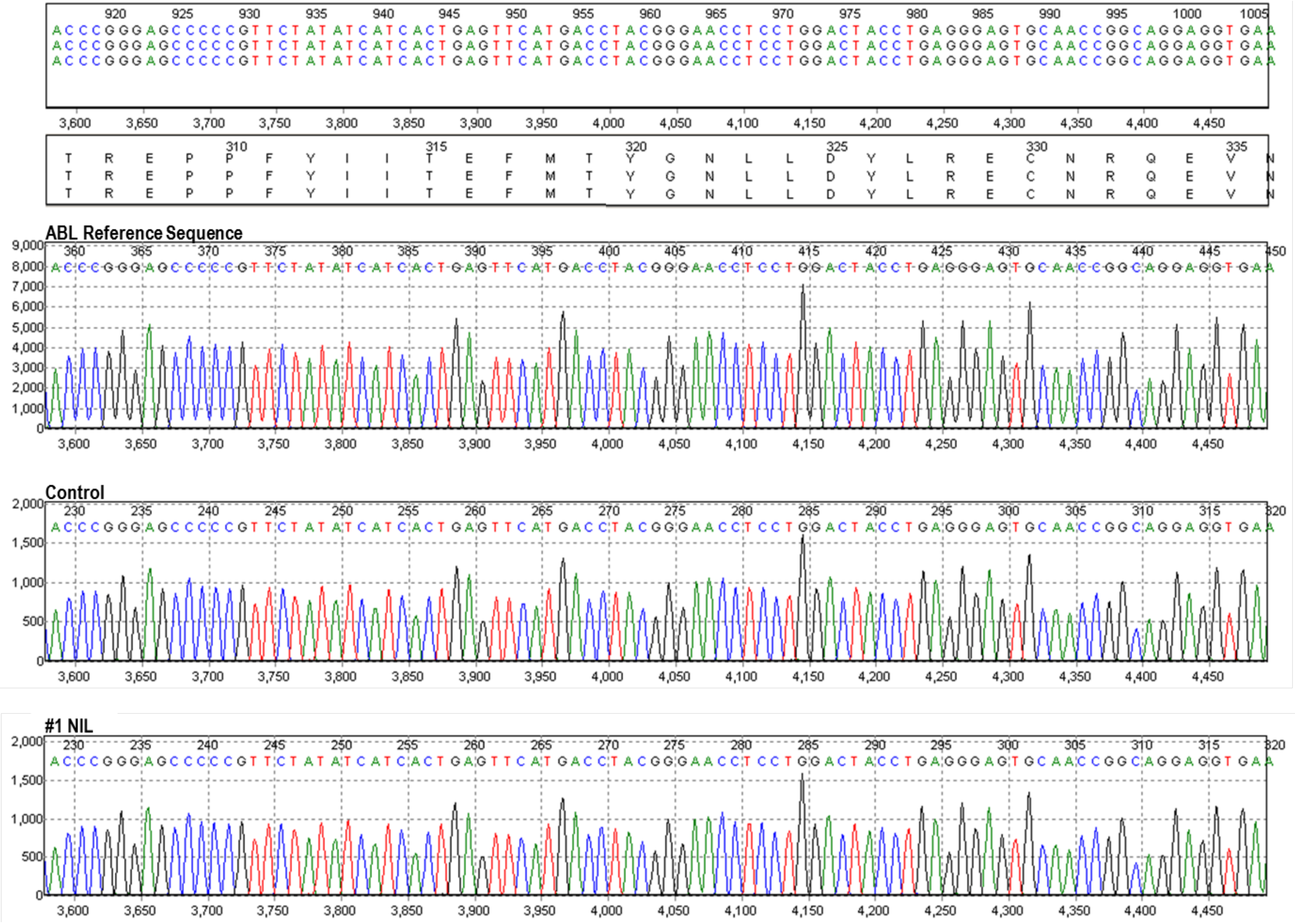
ABL Reference Sequence
Control
#1 NIL

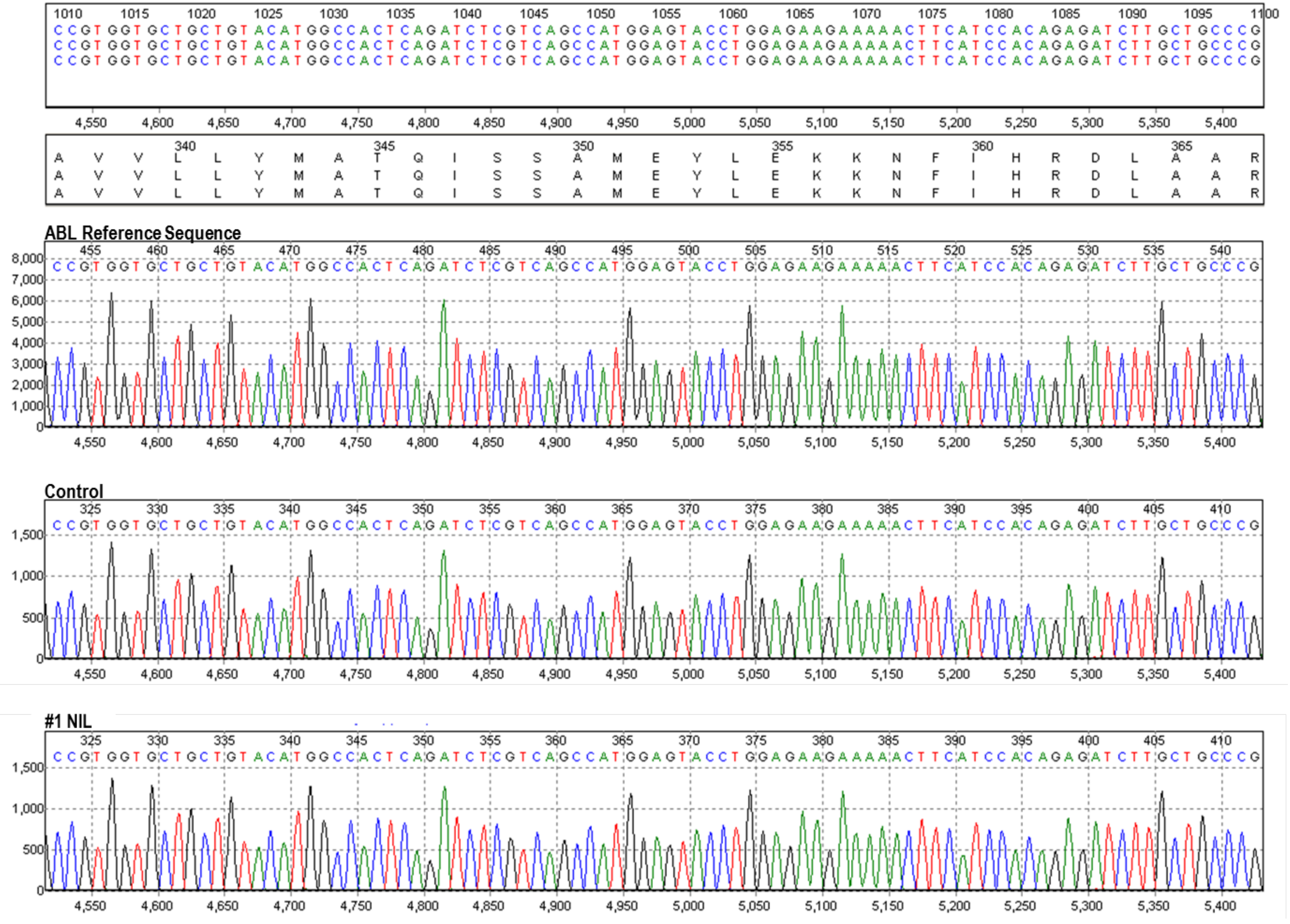

CCGTGGTGCTGCTGTACATGGCCACTCAGATCTCGTCAGCCATGGAGTACCTGGAGAAGAAAAACTTCATCCACAGAGATCTTGCTGCCCG
A V V L L Y M A T Q I S S A M E Y L E K K N F I H R D L A A R
ABL Reference Sequence
Control
#1 NIL

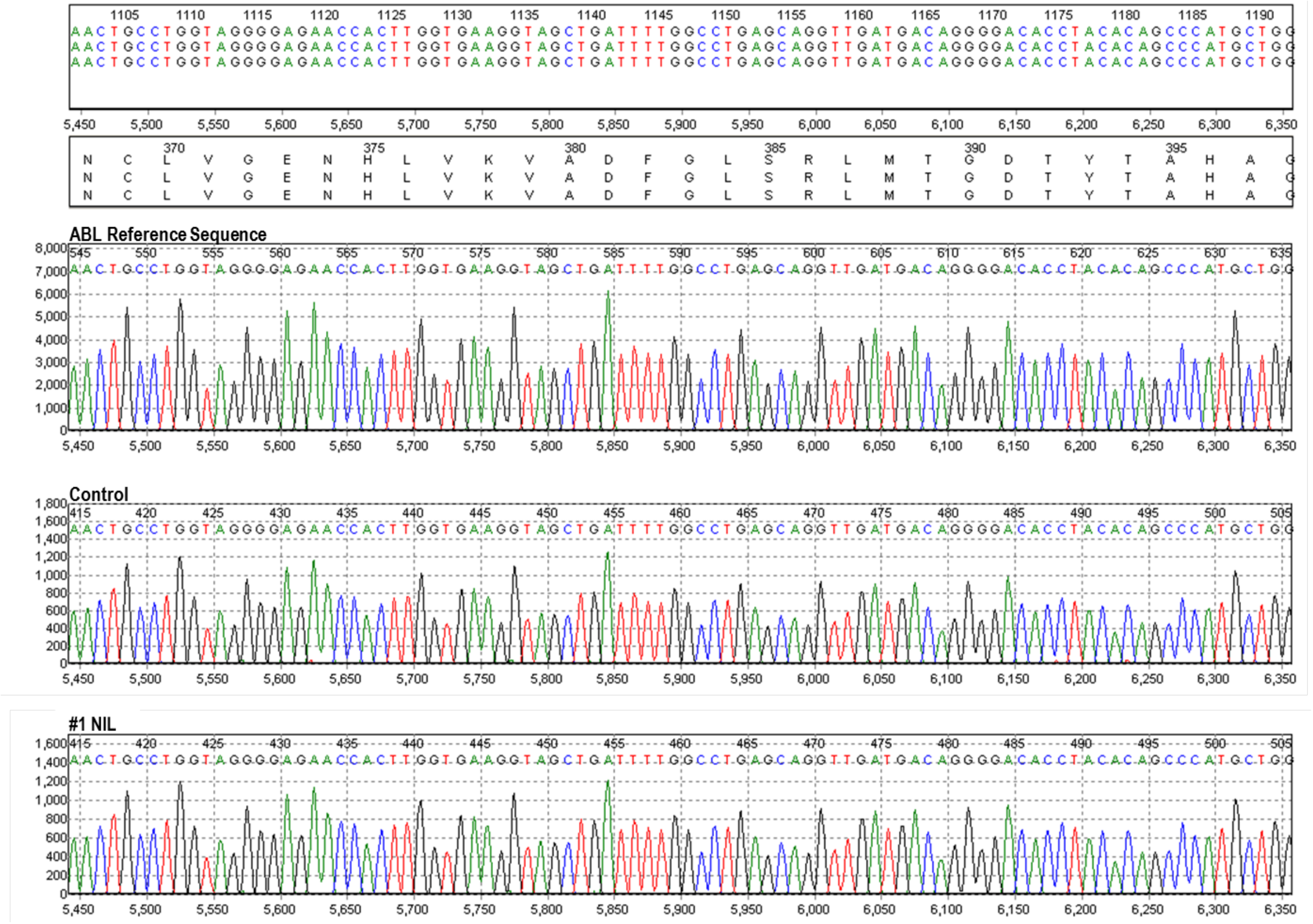
ABL Reference Sequence
Control
#1 NIL

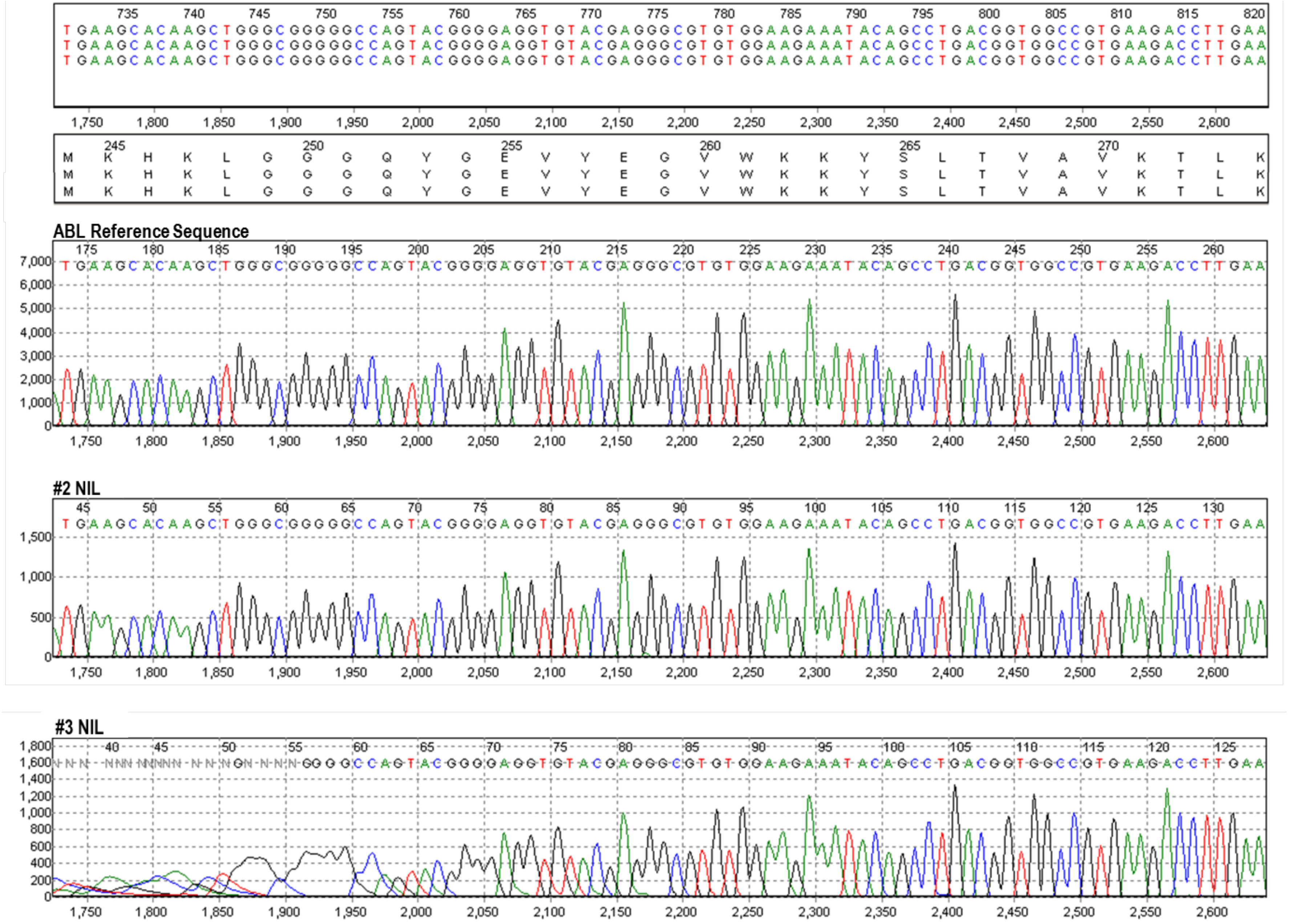
TGAAGCACAAGCTGGGCGGGGGCCAGTACGGGGAGGTGTACGAGGGCGTGTGGAAGAAATACAGCCTGACGGTGGCCGTGAAGACCTTGAA
M K H K L G G G Q Y G E V Y E G V W K K Y S L T V A V K T L K
ABL Reference Sequence
#2 NIL
#3 NIL

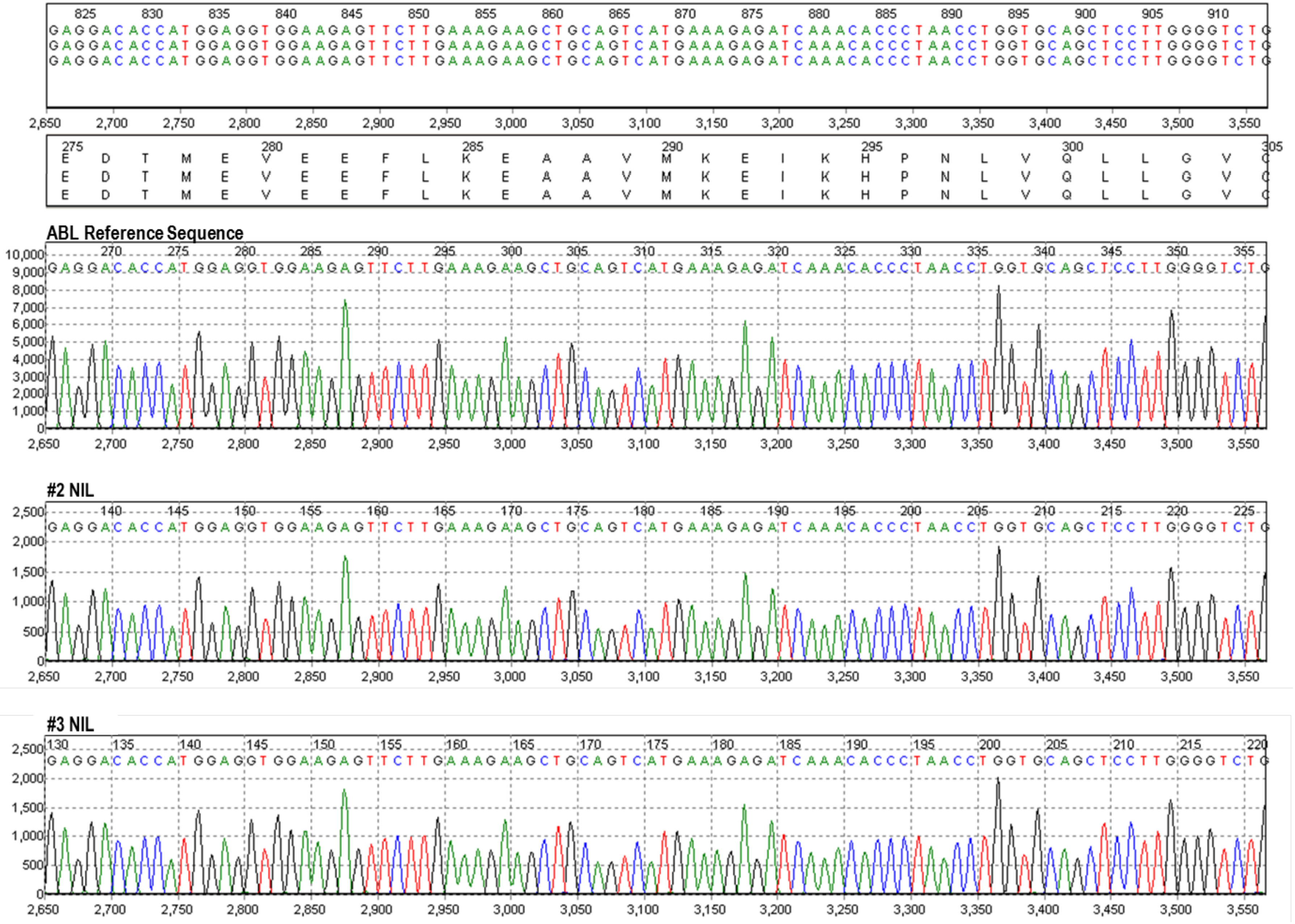
ABL Reference Sequence
#2 NIL
#3 NIL

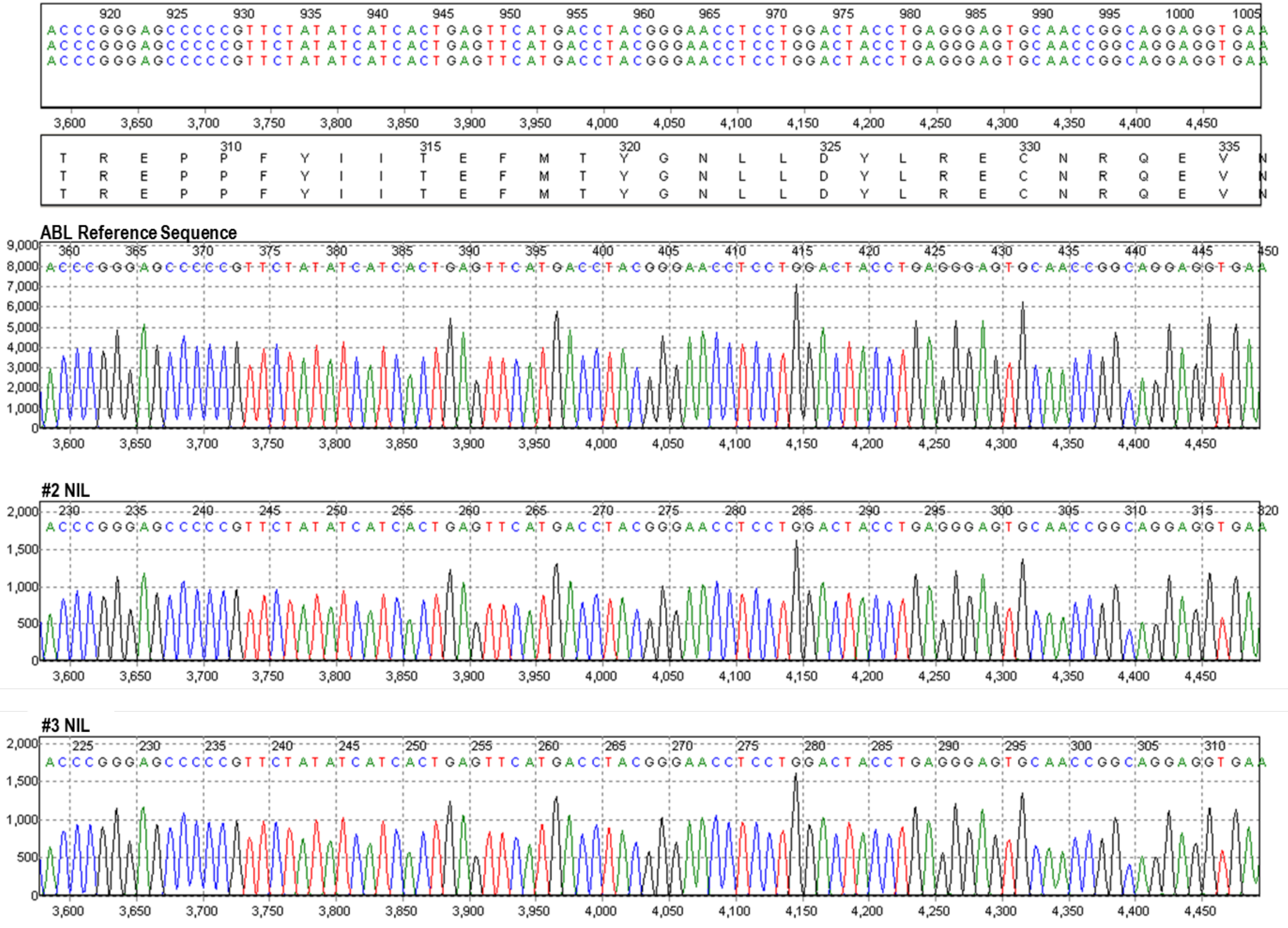
ACCCGGGAGCCCCCGTTCTATATCATCACTGAGTTCATGACCTACGGGAACCTCCTGGACTACCTGAGGGAGTGCAACCGGCAGGAGGTGAA
T R E P P F Y I I T E F M T Y G N L L D Y L R E C N R Q E V N
ABL Reference Sequence
#2 NIL
#3 NIL

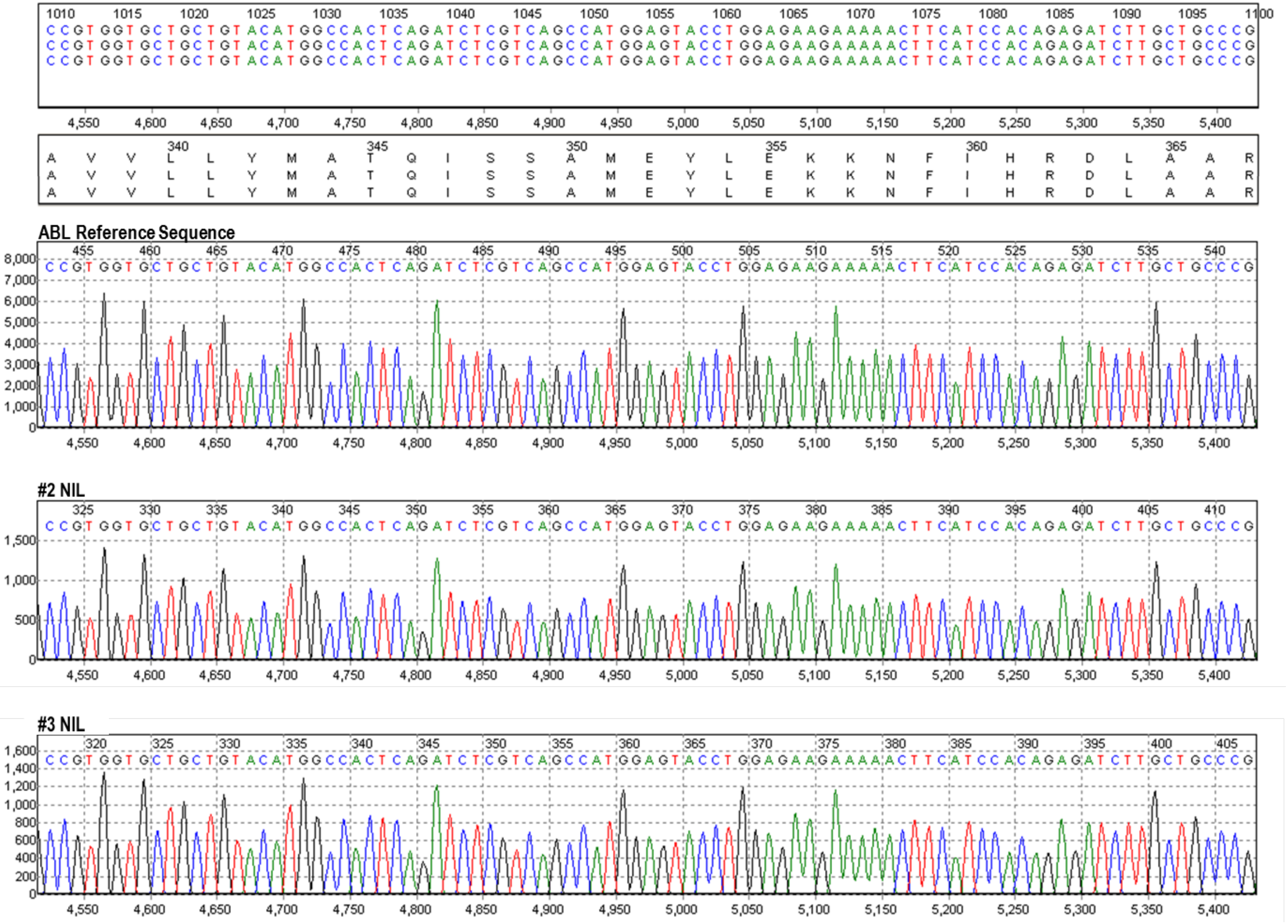
CCGTGGTGCTGCTGTACATGGCCACTCAGATCTCGTCAGCCATGGAGTACCTGGAGAAGAAAAACTTCATCCACAGAGATCTTGCTGCCCG
A V V L L Y M A T Q I S S A M E Y L E K K N F I H R D L A A R
ABL Reference Sequence
#2 NIL
#3 NIL

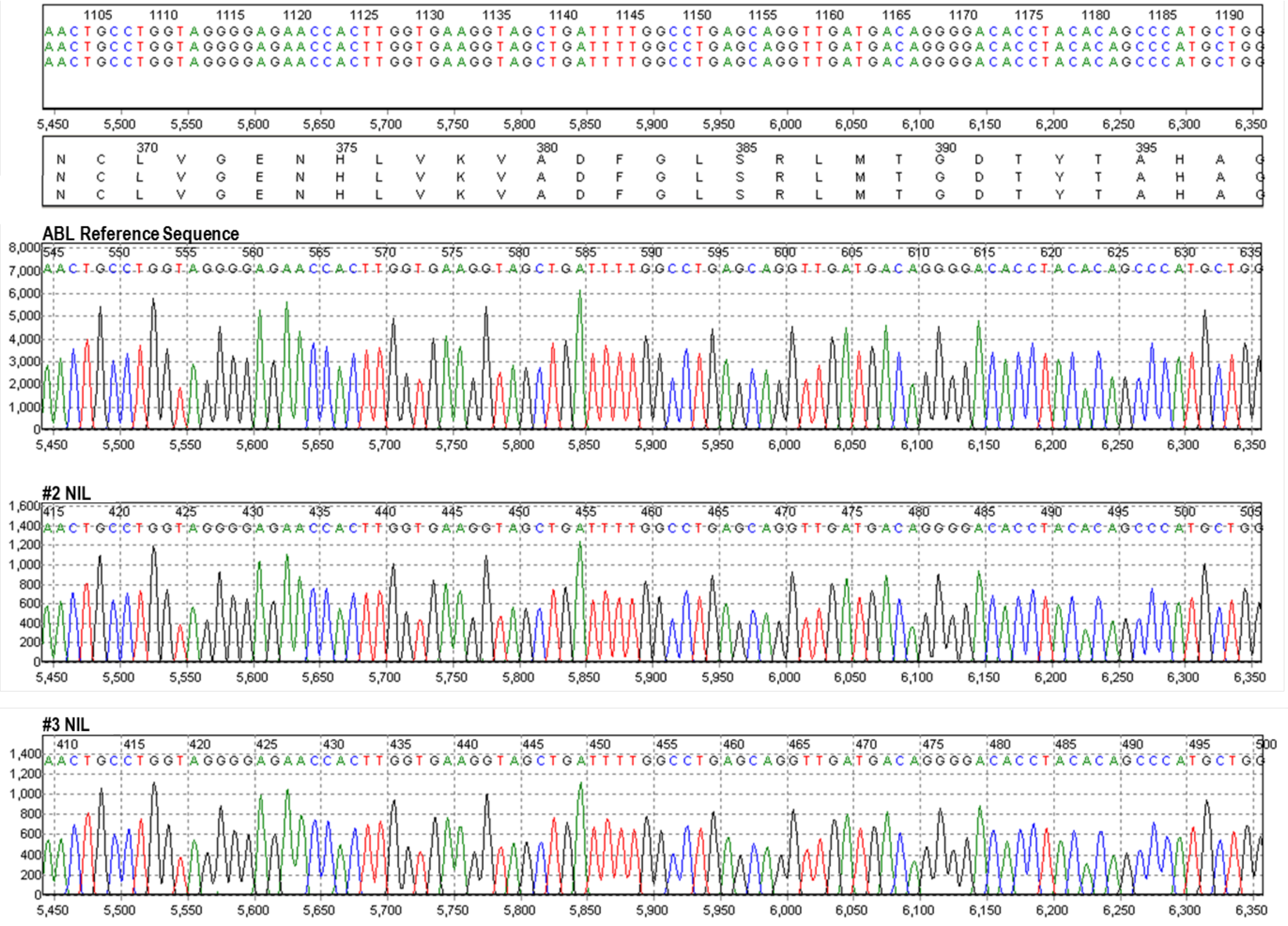
ABL Reference Sequence
#2 NIL
#3 NIL

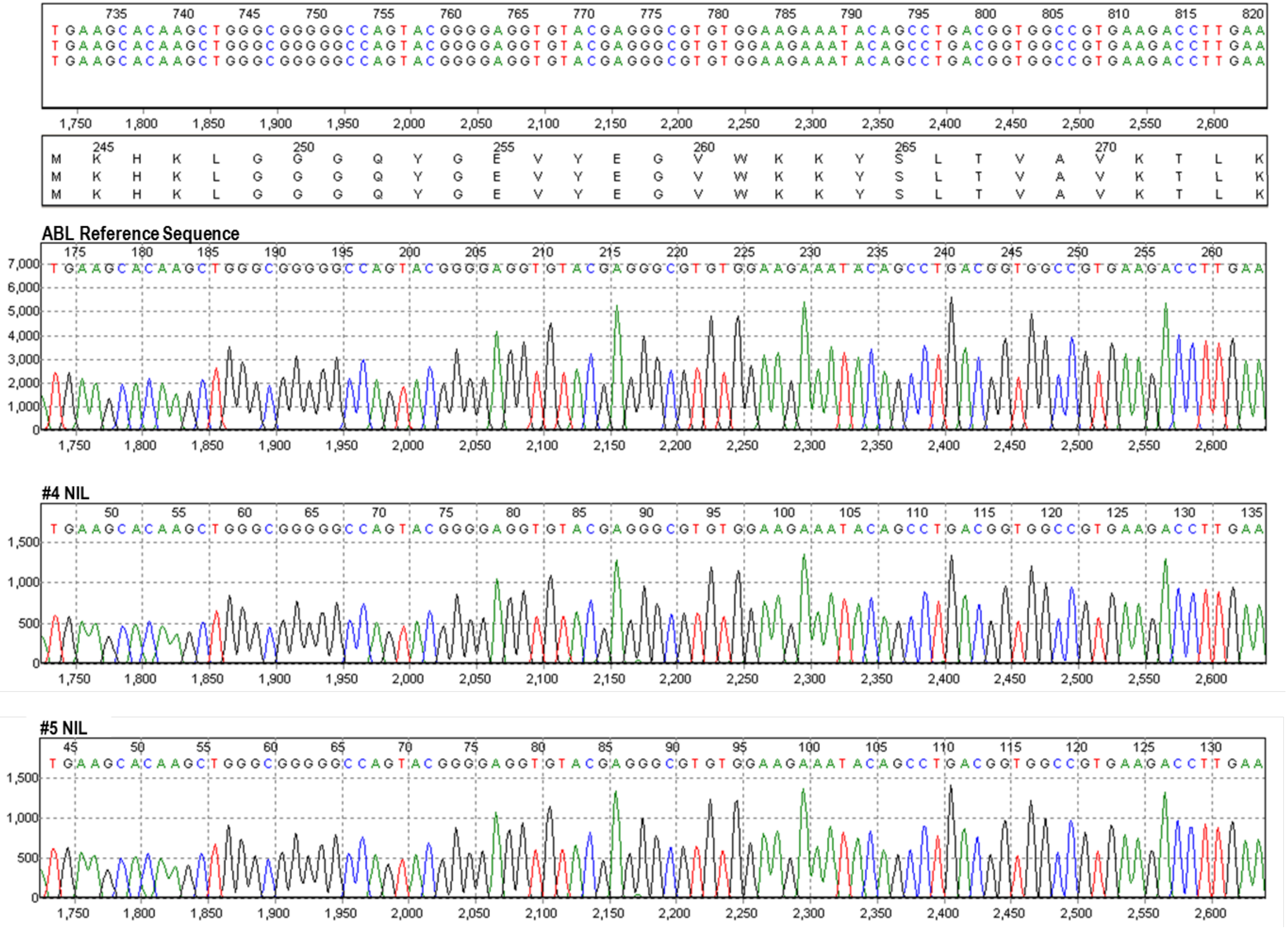
ABL Reference Sequence
#4 NIL
#5 NIL

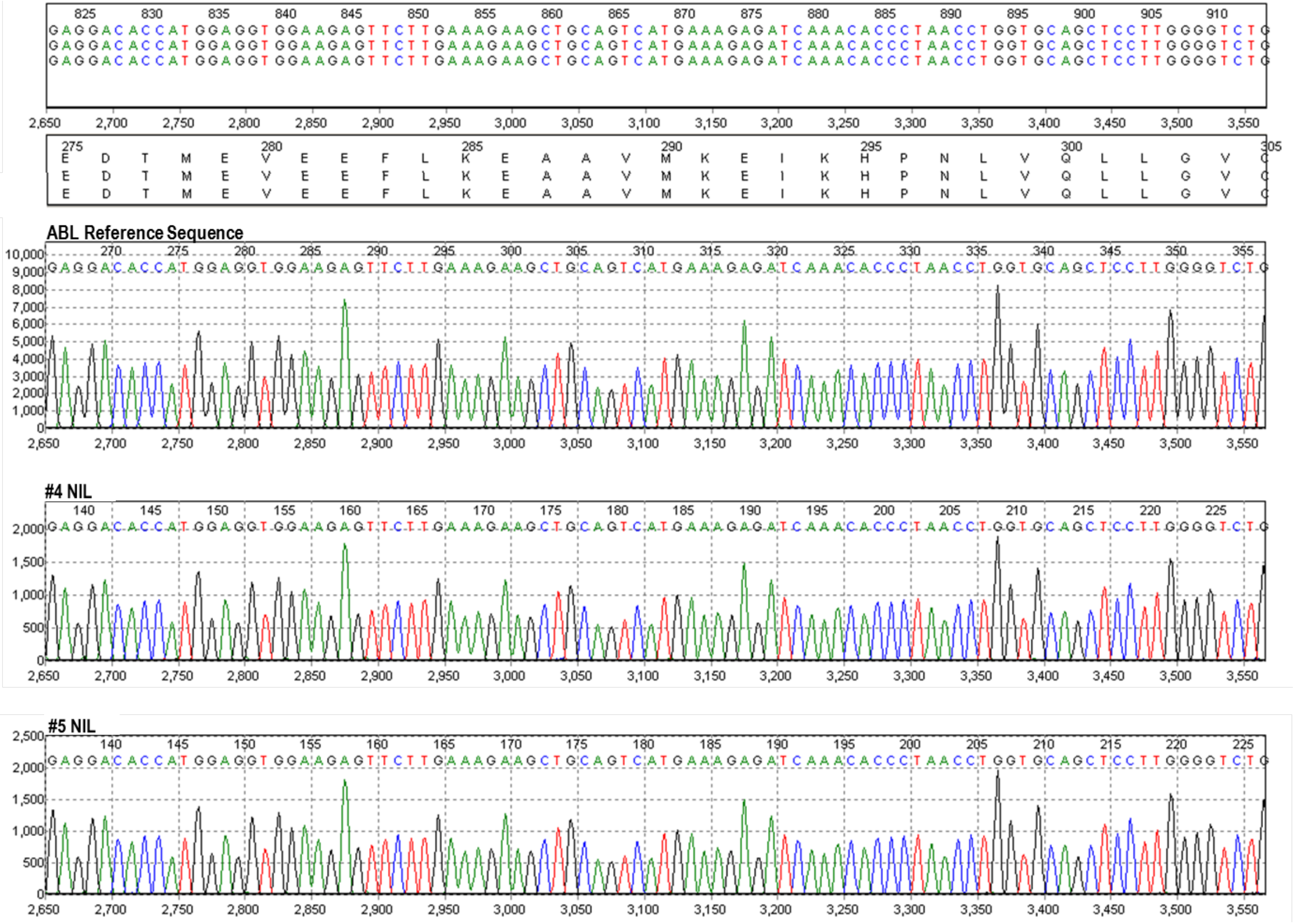
ABL Reference Sequence
#4 NIL
#5 NIL

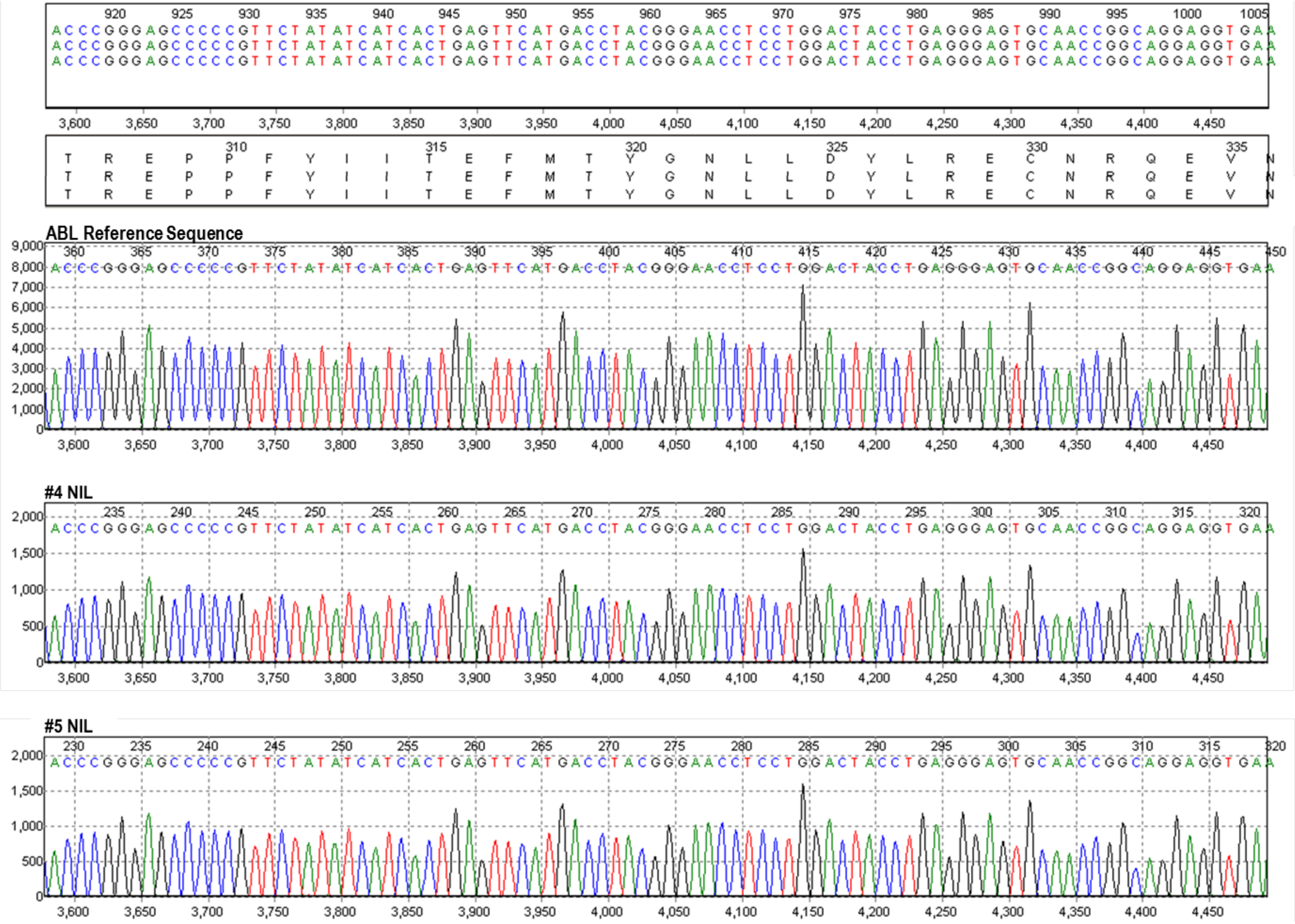

ACCCGGGAGCCCCCGTTCTATATCATCACTGAGTTCATGACCTACGGGAACCTCCTGGACTACCTGAGGGAGTGCAACCGGCAGGAGGTGAA
T R E P P F Y I I T E F M T Y G N L L D Y L R E C N R Q E V N
ABL Reference Sequence
#4 NIL
#5 NIL

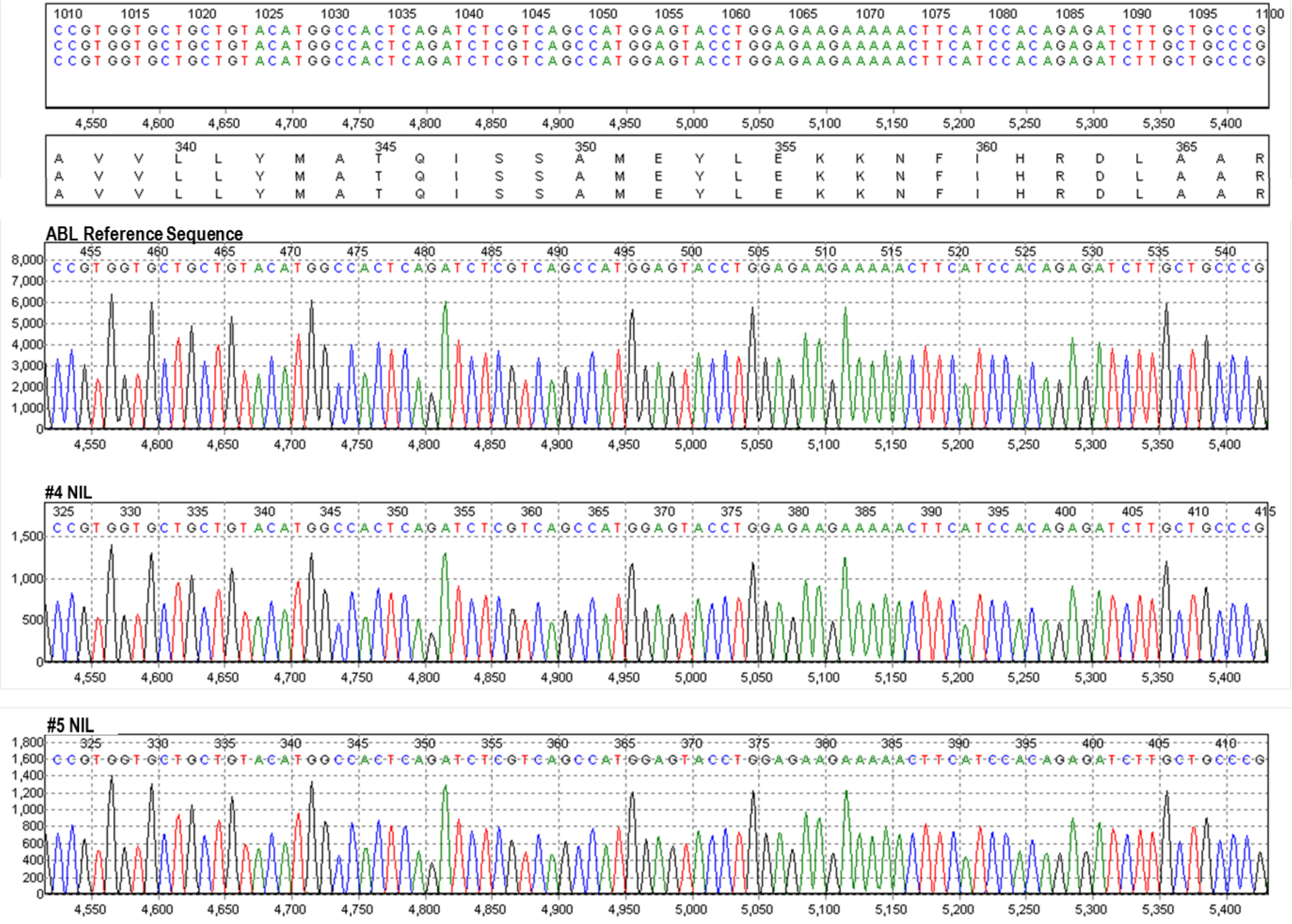

ABL Reference Sequence
#4 NIL
#5 NIL

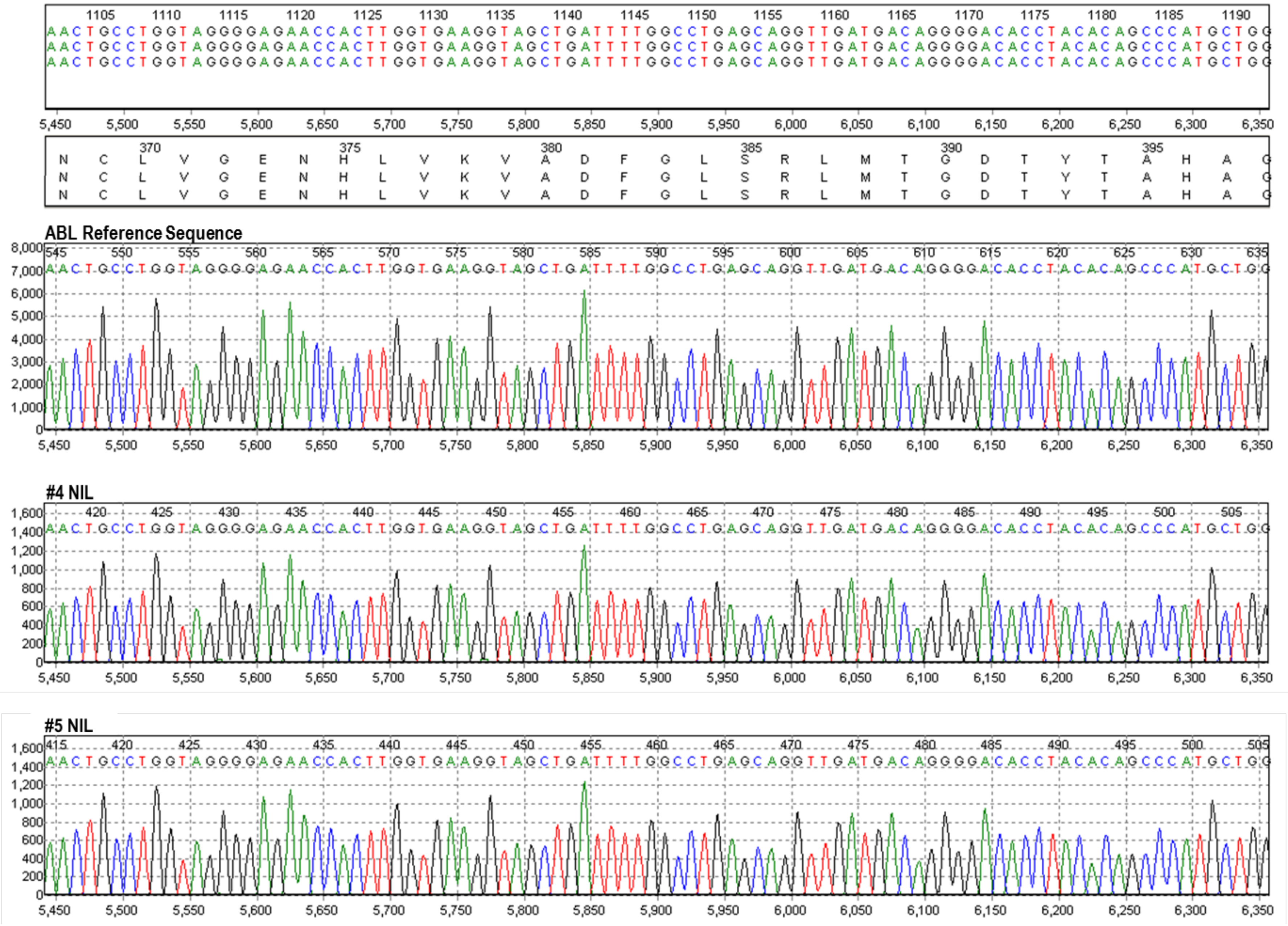
ABL Reference Sequence
#4 NIL
#5 NIL

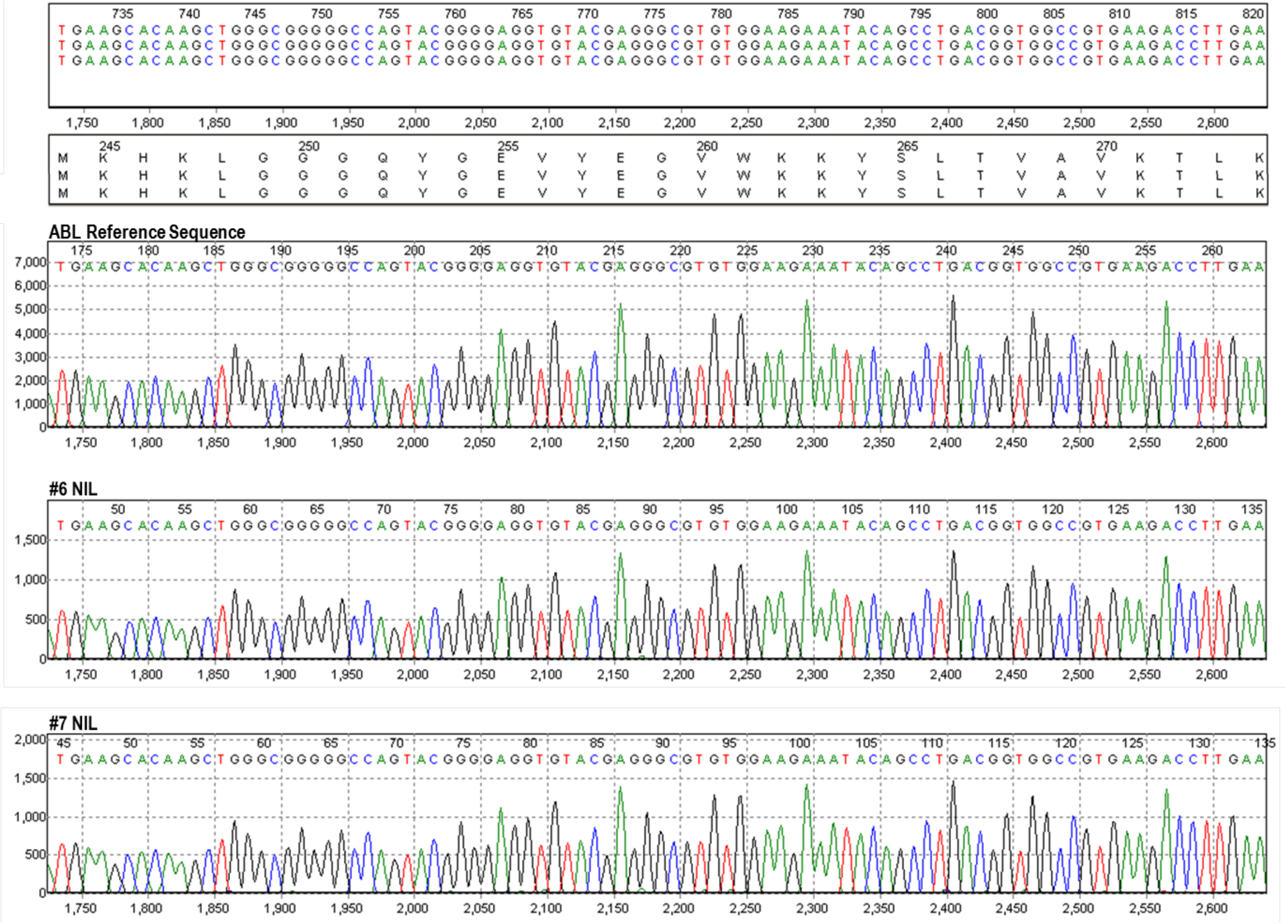
ABL Reference Sequence
#6 NIL
#7 NIL

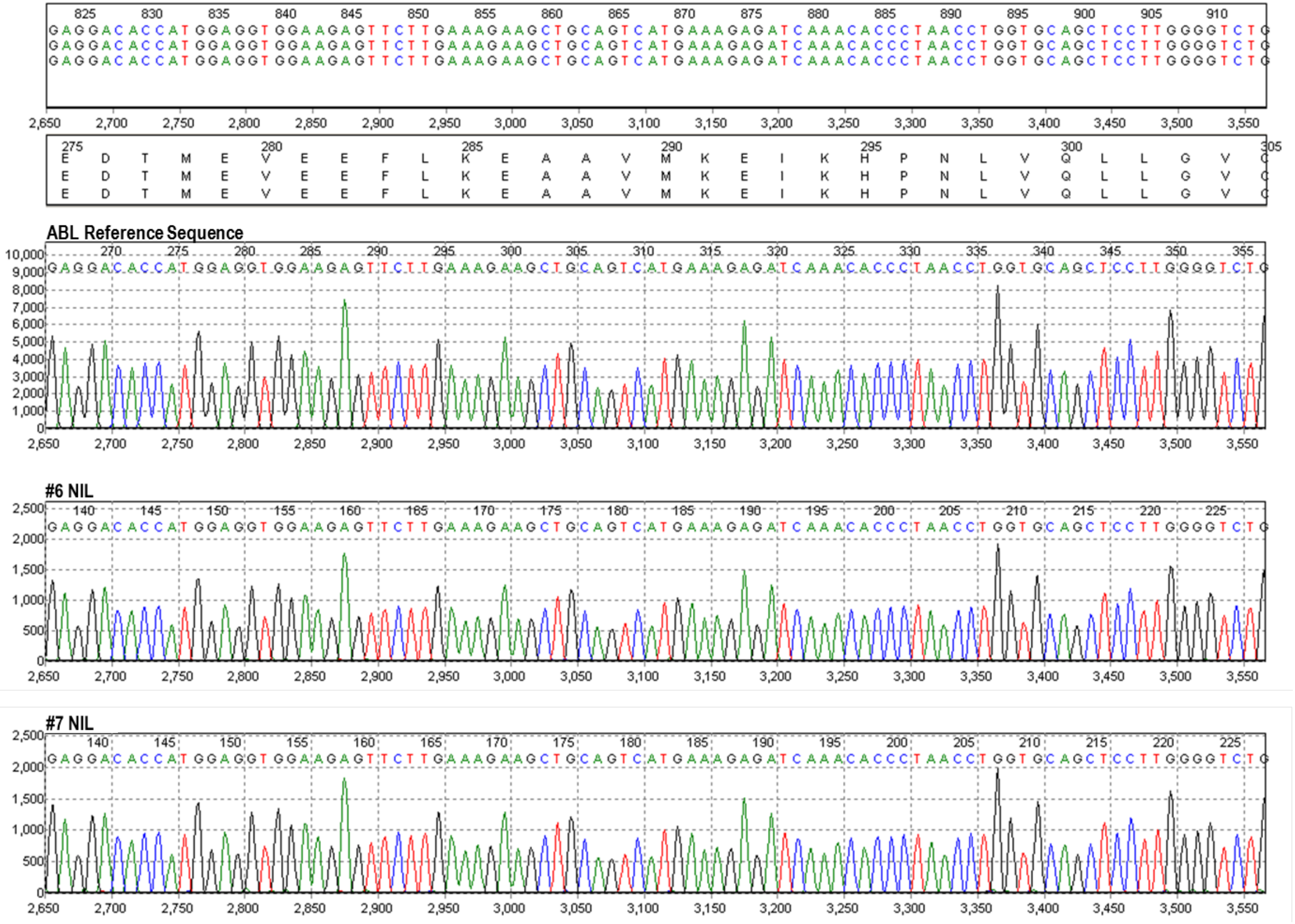
ABL Reference Sequence
#6 NIL
#7 NIL

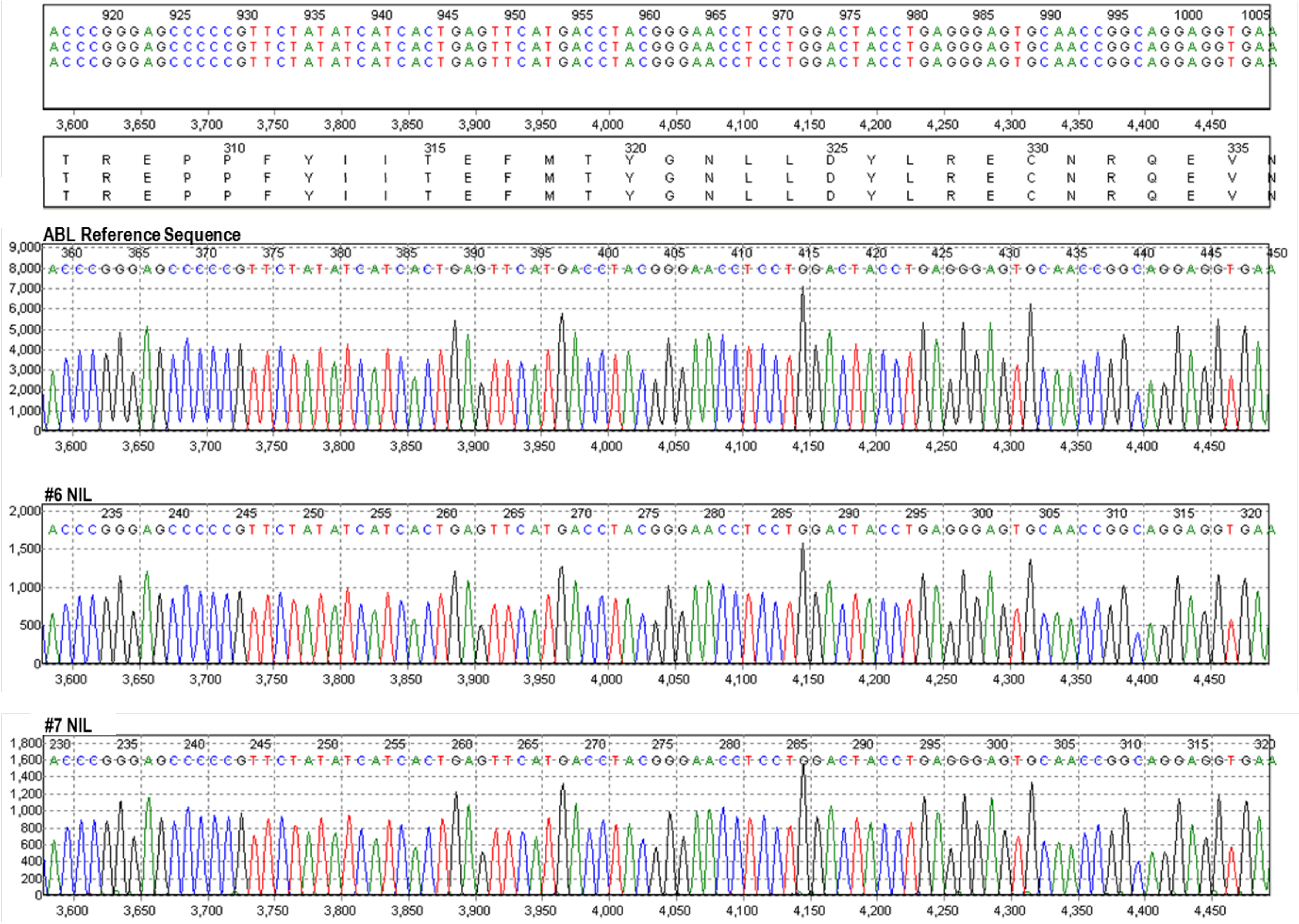
ABL Reference Sequence
#6 NIL
#7 NIL

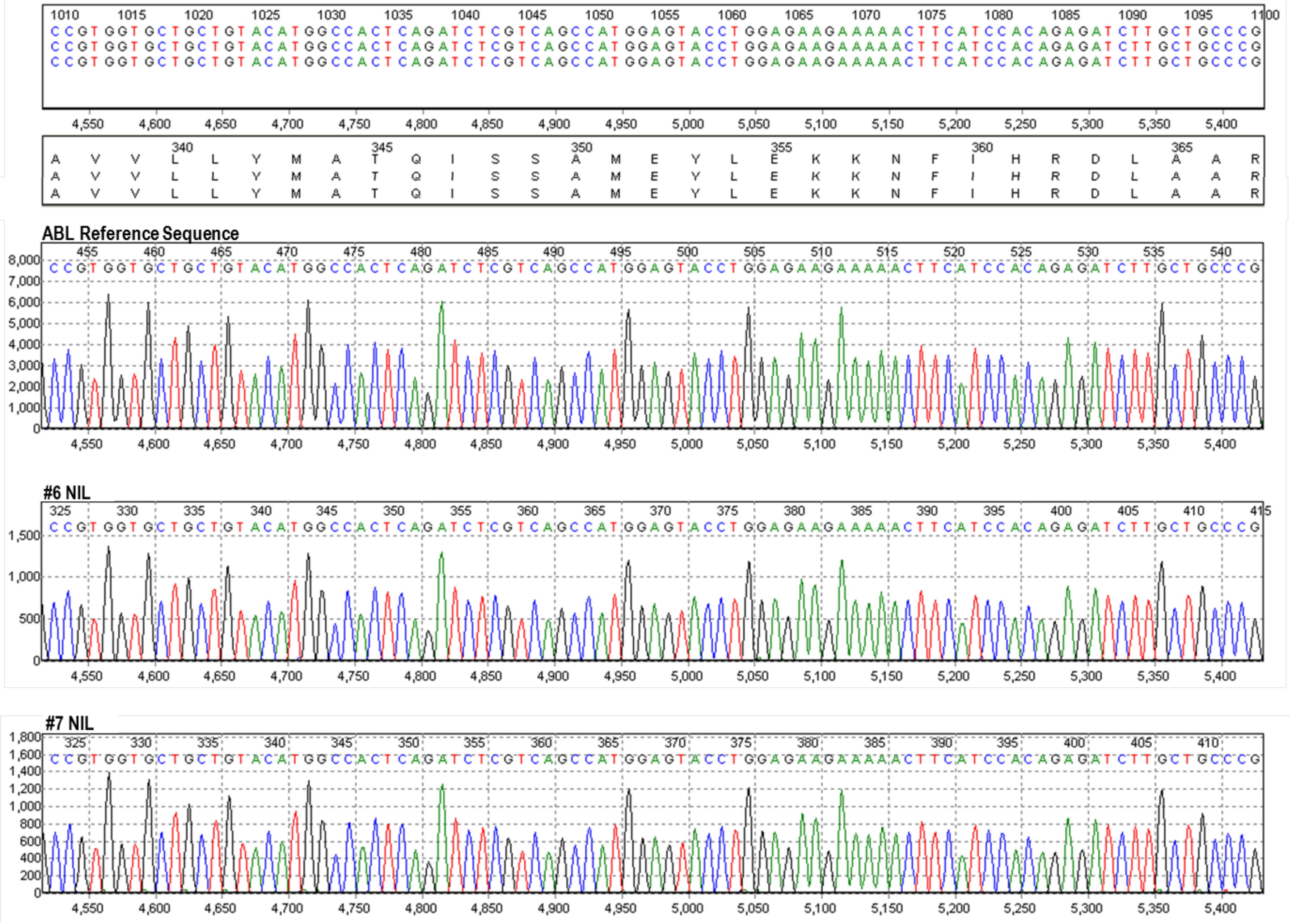
ABL Reference Sequence
#6 NIL
#7 NIL

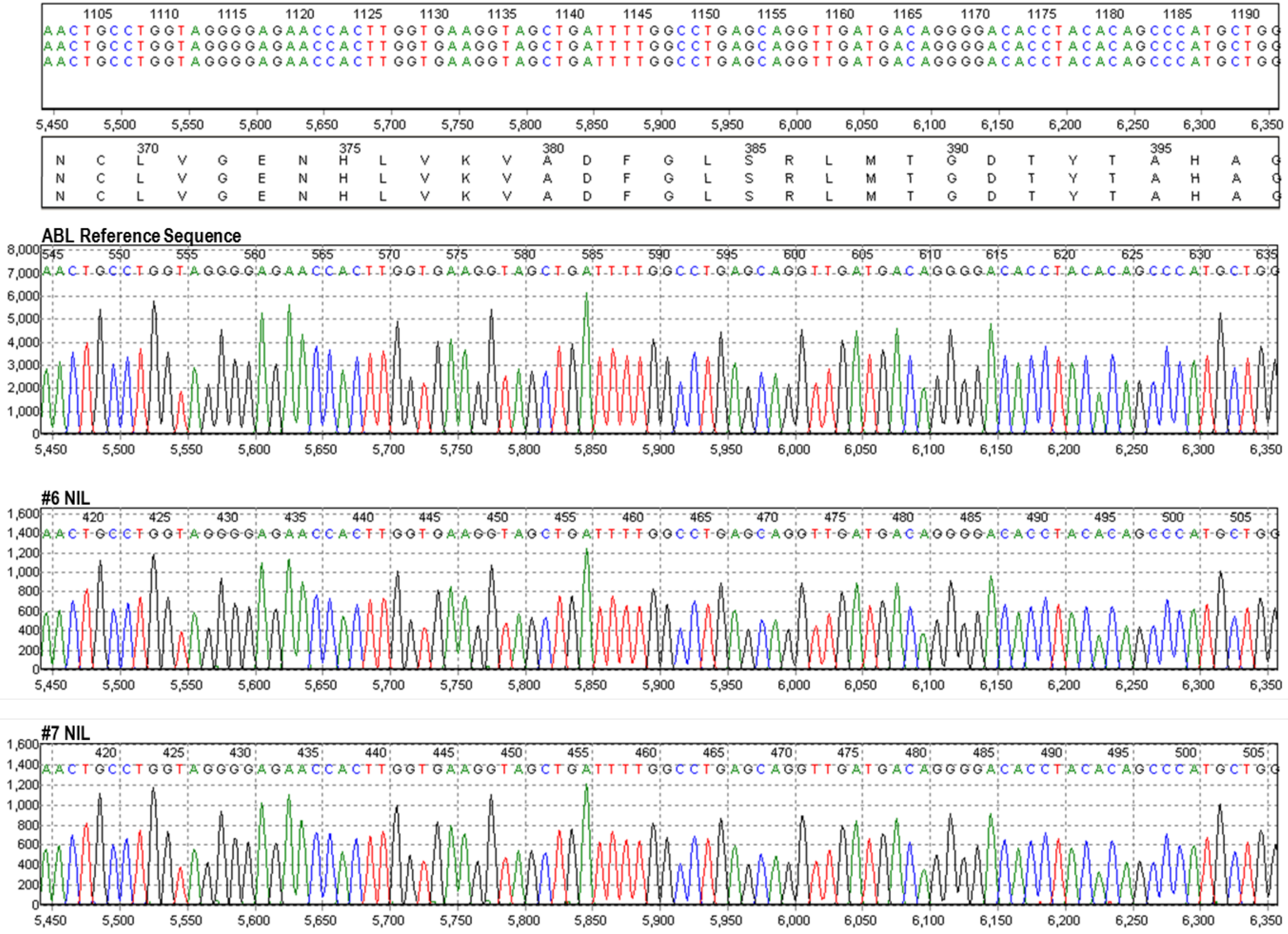

AACTGCCTGGTAGGGGAGAACCACTTGGTGAAGGTAGCTGATTTTGGCCTGAGCAGGTTGATGACAGGGGACACCTACACAGCCCATGCTGG
N C L V G E N H L V K V A D F G L S R L M T G D T Y T A H A G
ABL Reference Sequence
#6 NIL
#7 NIL

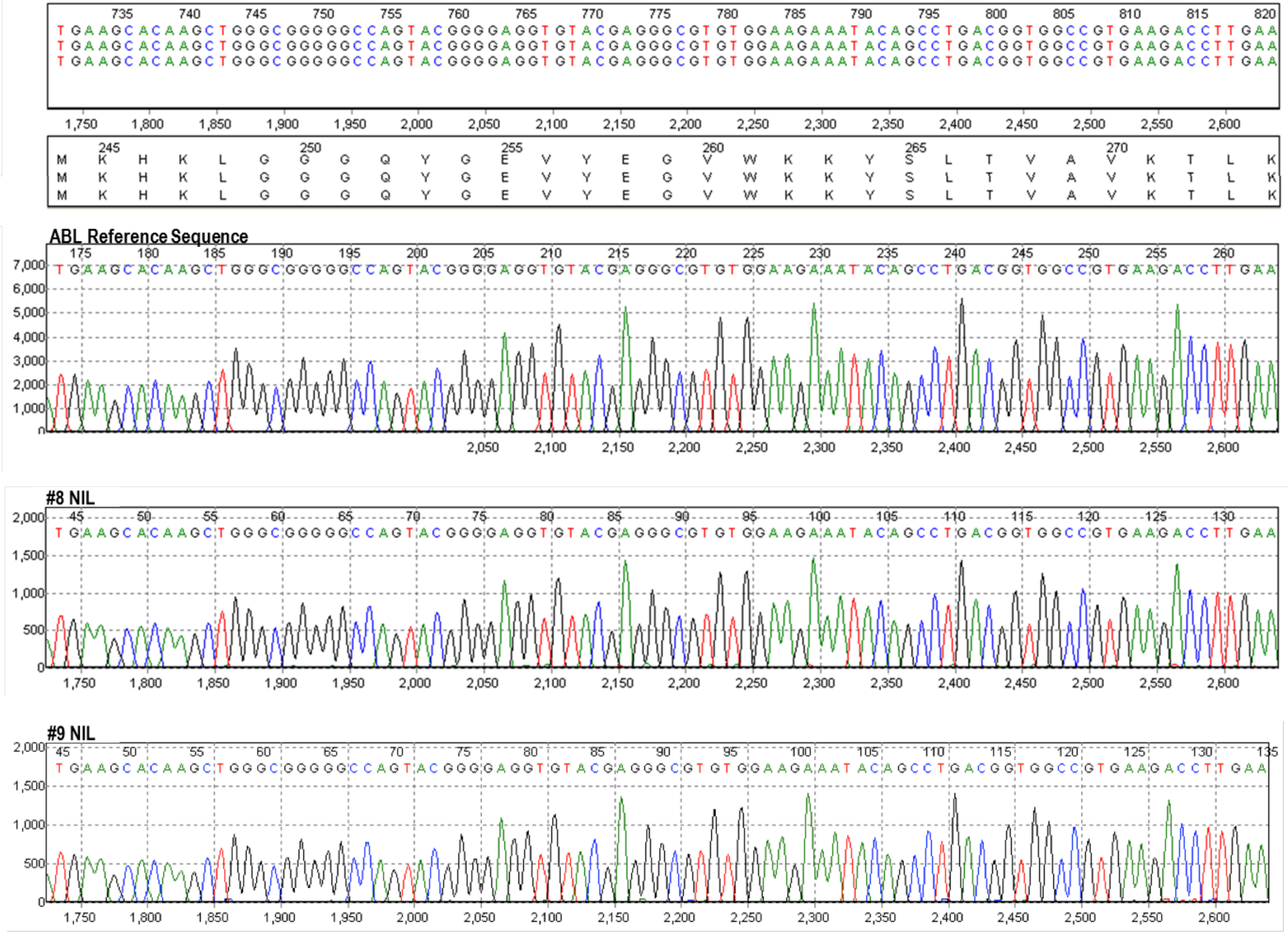
TGAAGCACAAGCTGGGCGGGGGCCAGTACGGGGAGGTGTACGAGGGCGTGTGGAAGAAATACAGCCTGACGGTGGCCGTGAAGACCTTGAA
M K H K L G G G Q Y G E V Y E G V W K K Y S L T V A V K T L K
ABL Reference Sequence
#8 NIL
#9 NIL

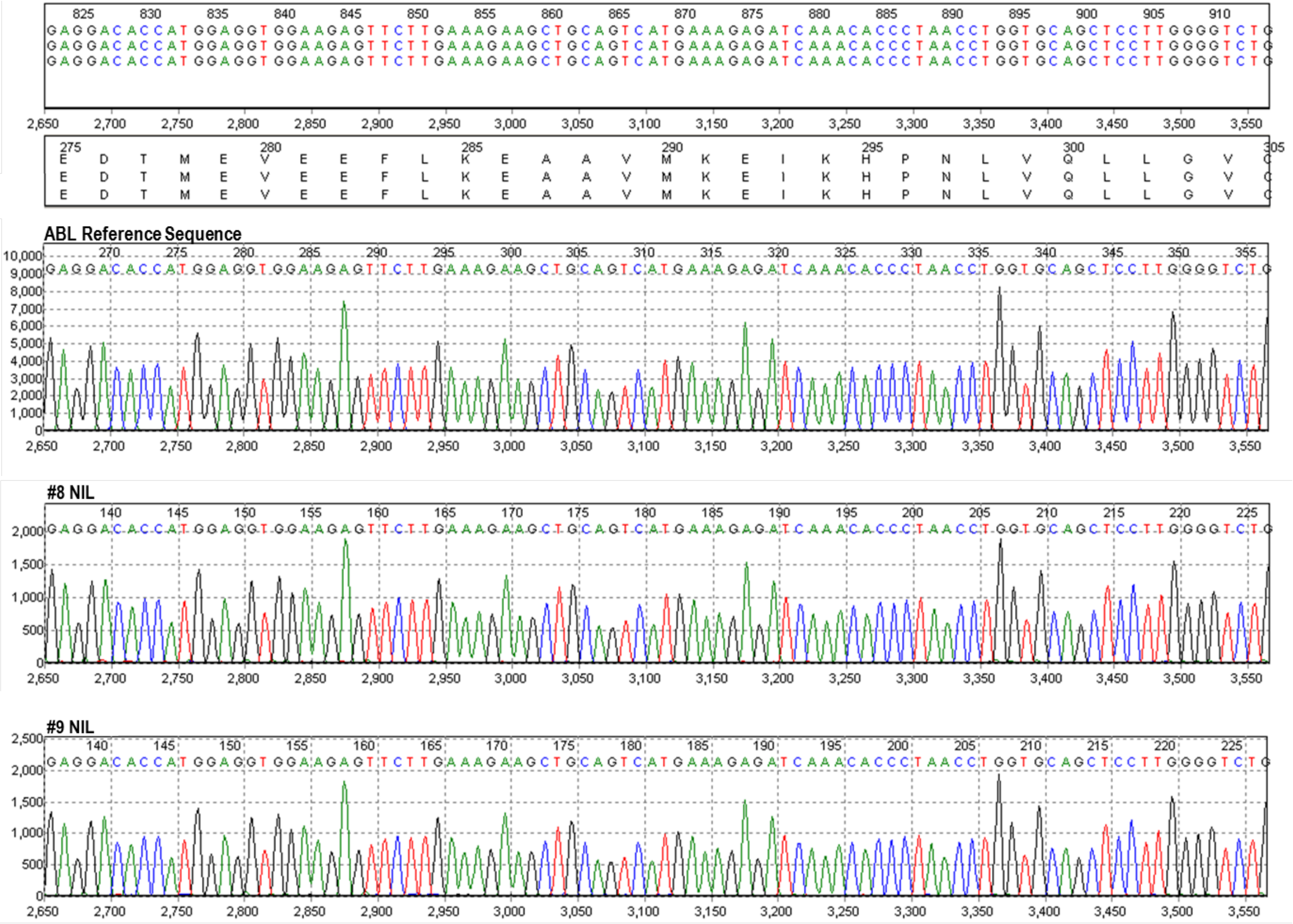

GAGGACACCATGGAGGTGGAAGAGTTCTTGAAAGAAGCTGCAGTCATGAAAGAGATCAAACACCCTAACCTGGTGCAGCTCCTTGGGGTCTG
E D T M E V E E F L K E A A V M K E I K H P N L V Q L L G V
ABL Reference Sequence
#8 NIL
#9 NIL

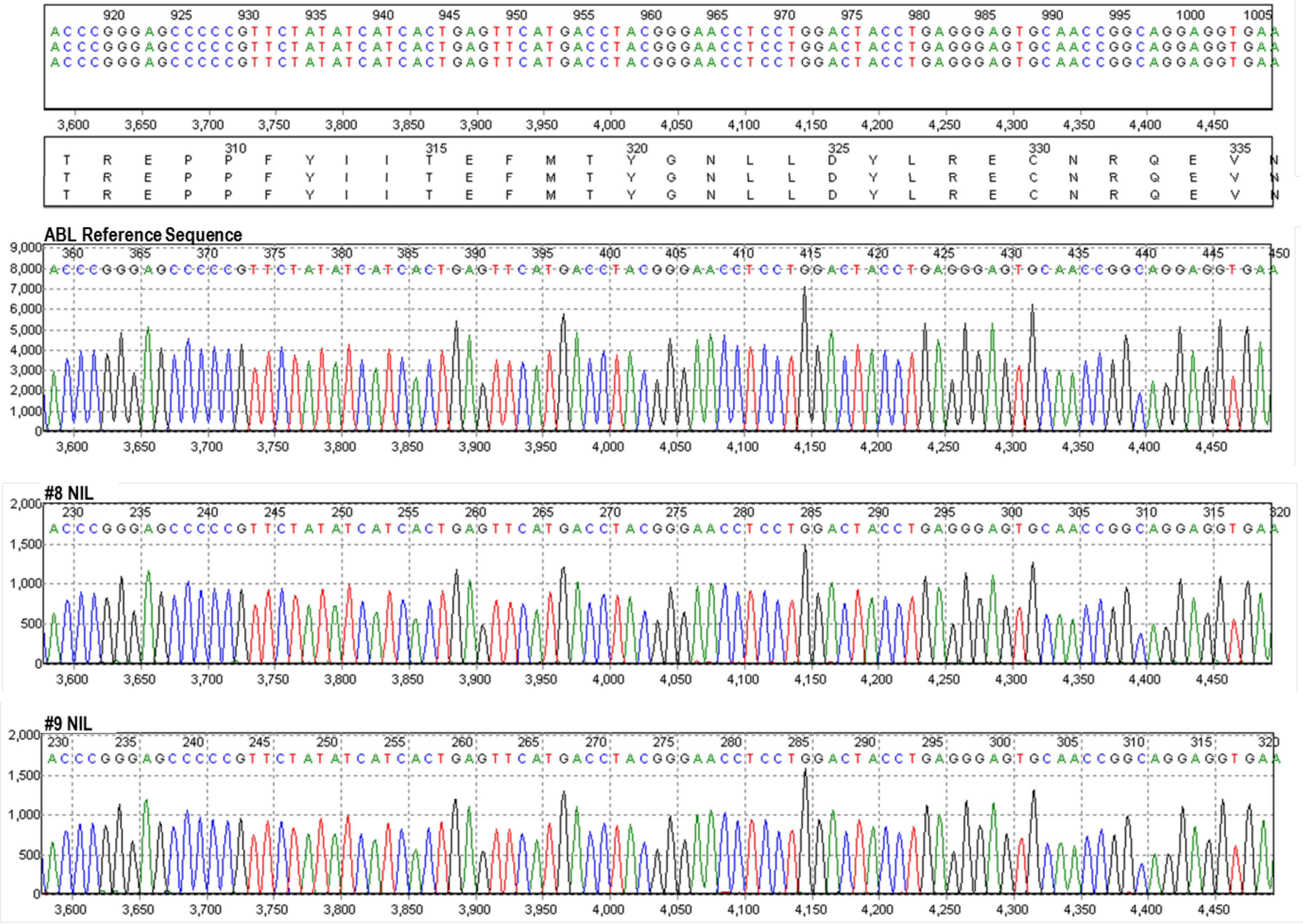

ABL Reference Sequence
#8 NIL
#9 NIL

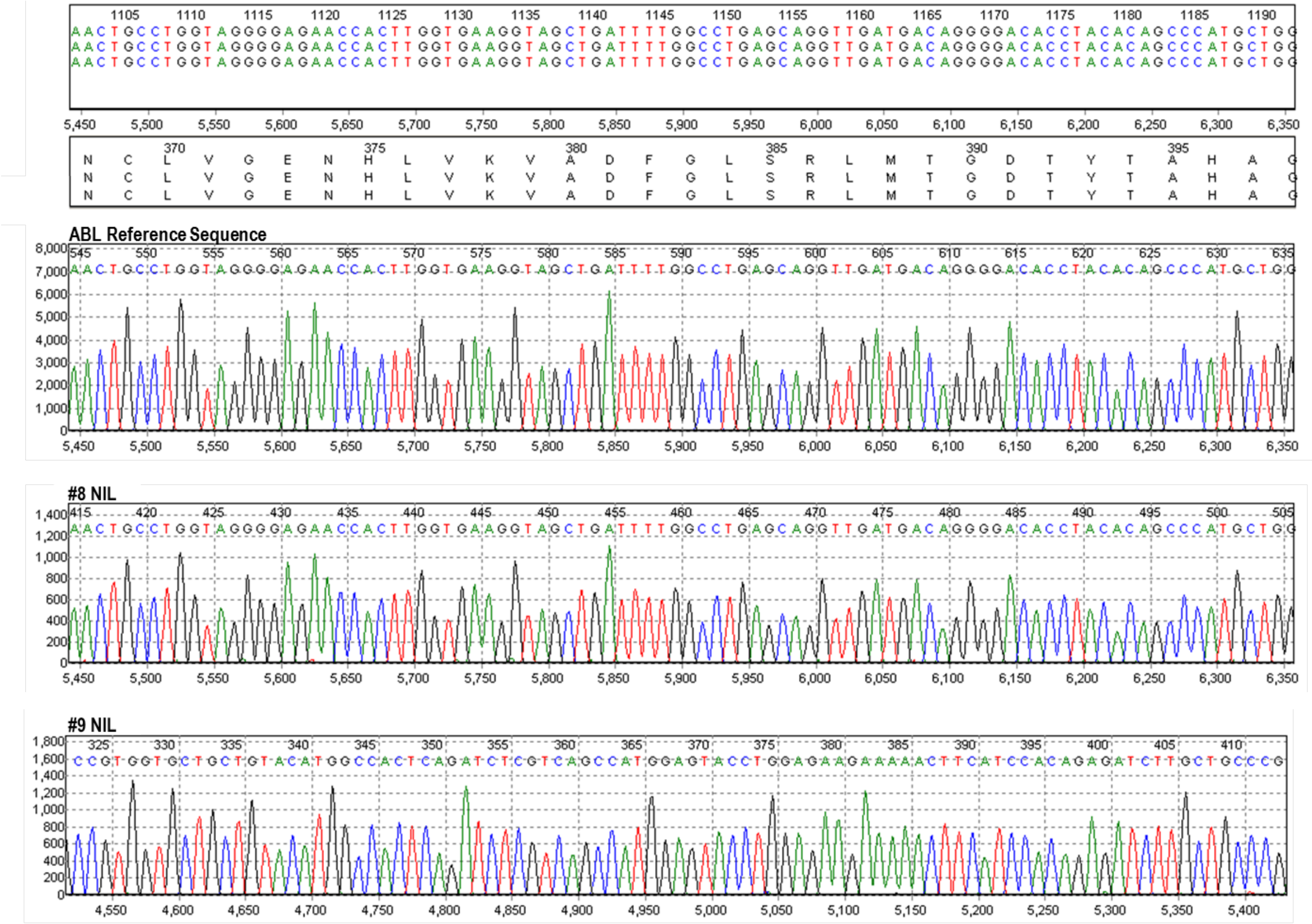
ABL Reference Sequence
#8 NIL
#9 NIL

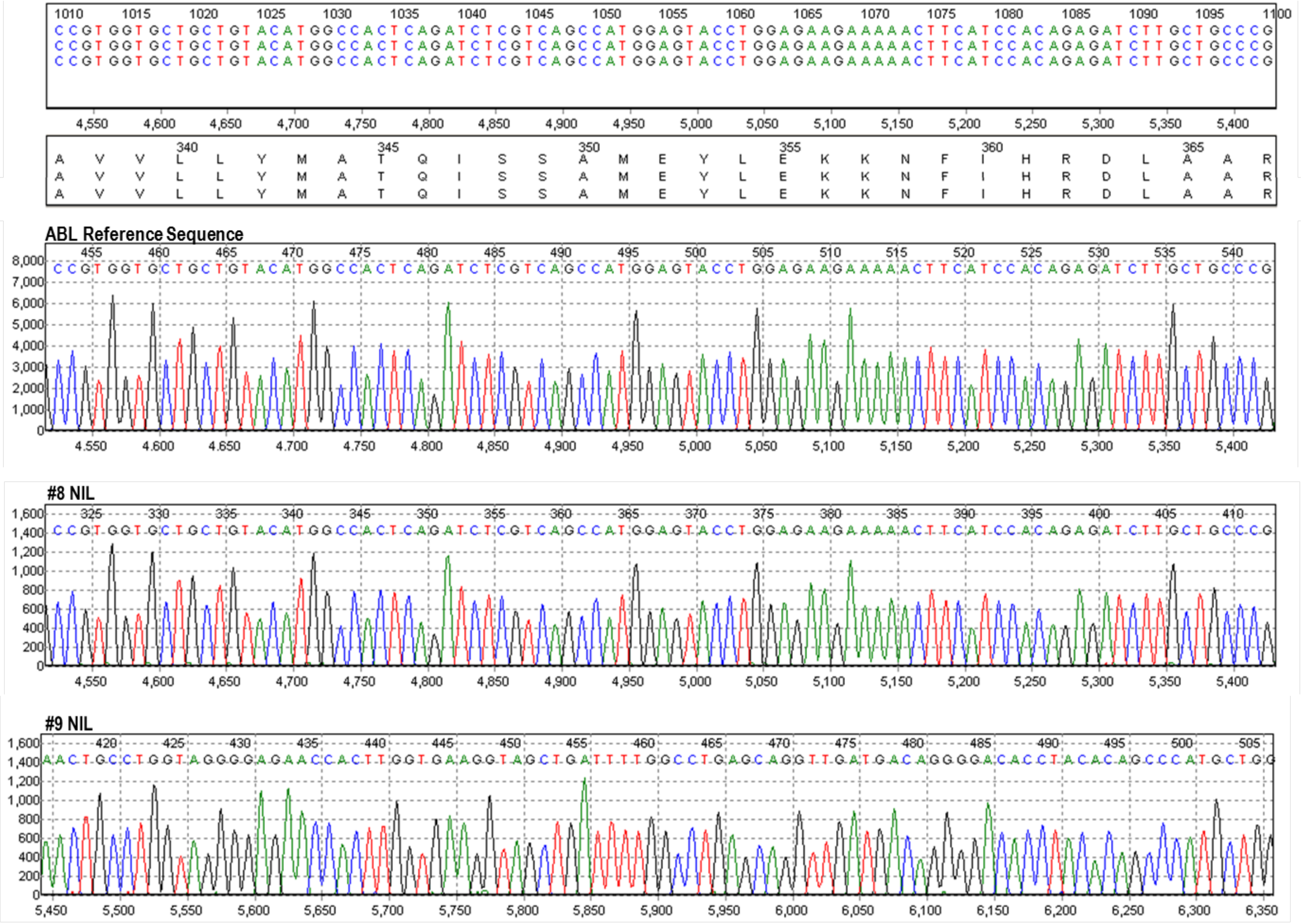
ABL Reference Sequence
#8 NIL
#9 NIL

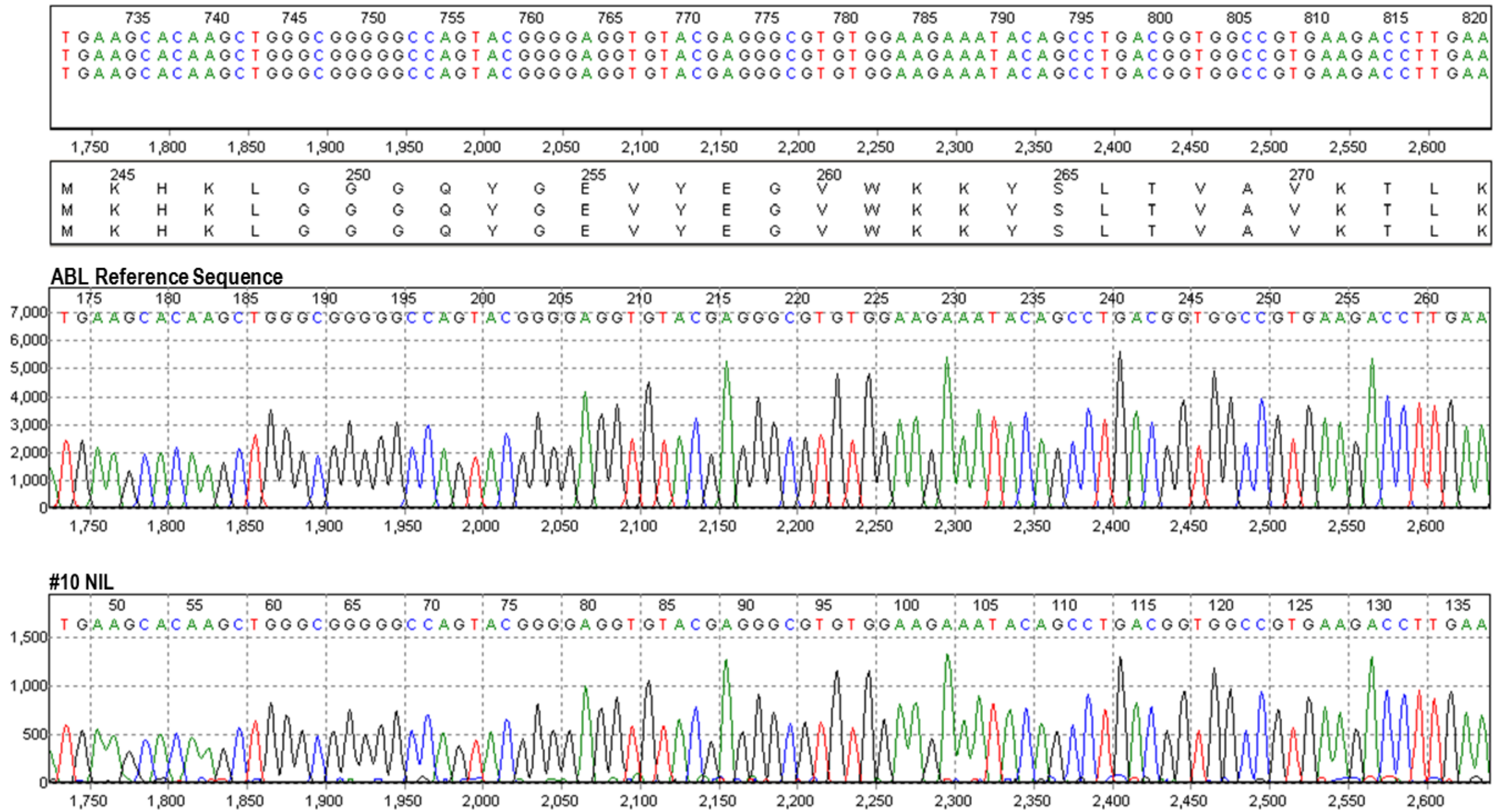
M K H K L G G G Q Y G E V Y E G V W K K Y S L T V A V K T L K
ABL Reference Sequence
#10 NIL

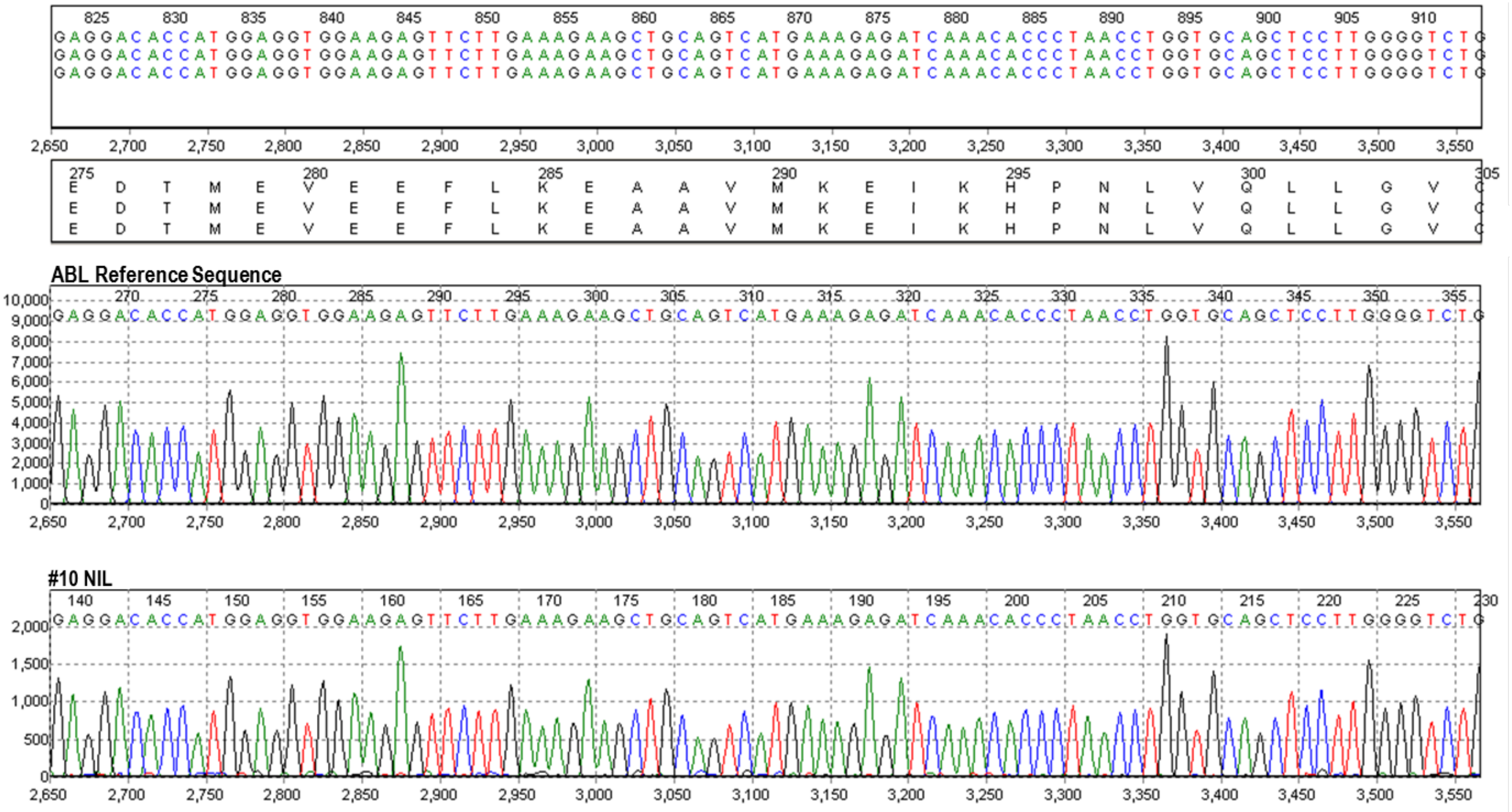
GAGGACACCATGGAGGTGGAAGAGTTCTTGAAAGAAGCTGCAGTCATGAAAGAGATCAAACACCCTAACCTGGTGCAGCTCCTTGGGGTCTG
E D T M E V E E F L K E A A V M K E I K H P N L V Q L L G V C
ABL Reference Sequence
#10 NIL

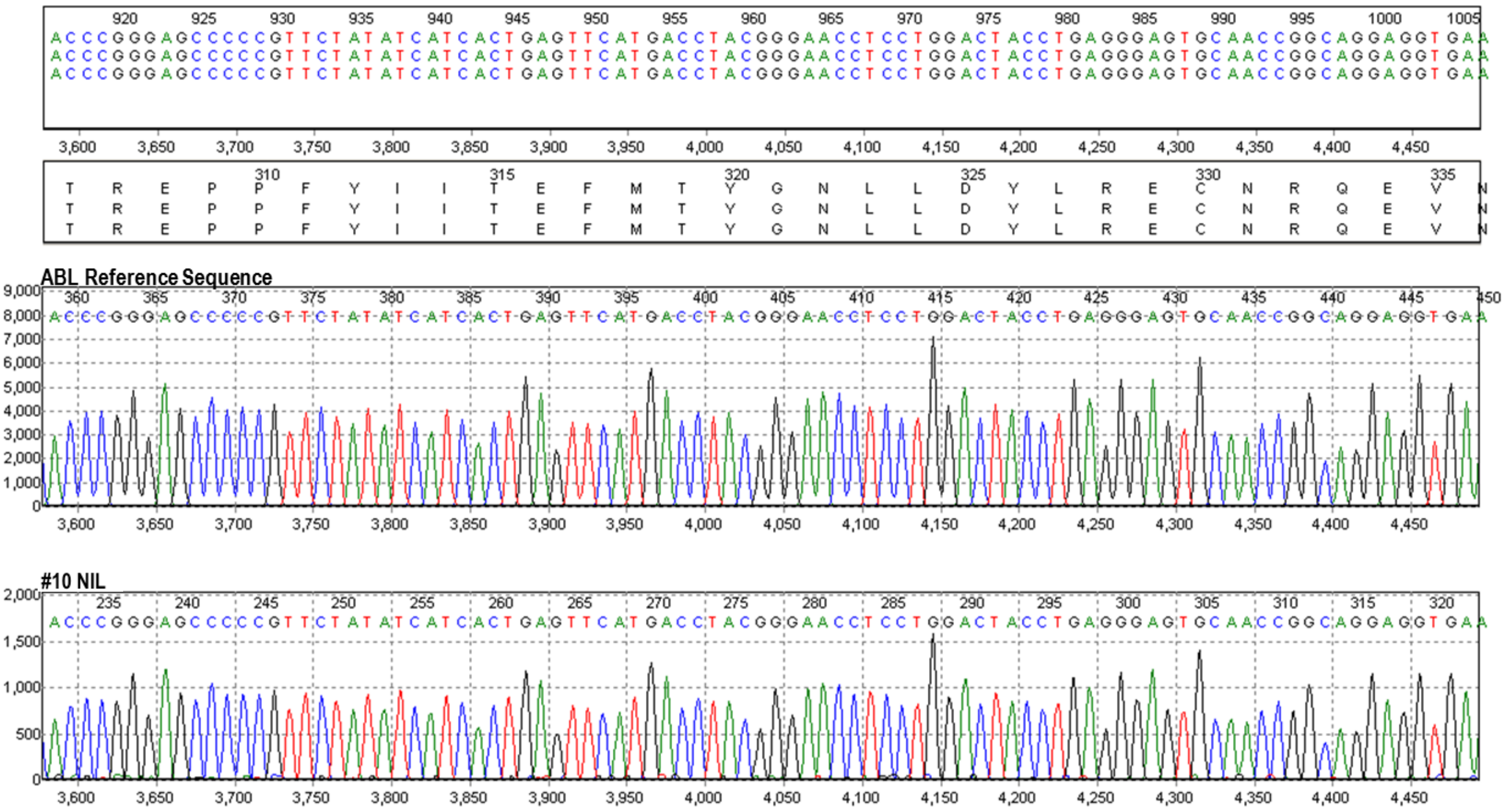

ACCCGGGAGCCCCCGTTCTATATCATCACTGAGTTCATGACCTACGGGAACCTCCTGGACTACCTGAGGGAGTGCAACCGGCAGGAGGTGAA
T R E P P F Y I I T E F M T Y G N L L D Y L R E C N R Q E V N
ABL Reference Sequence
#10 NIL

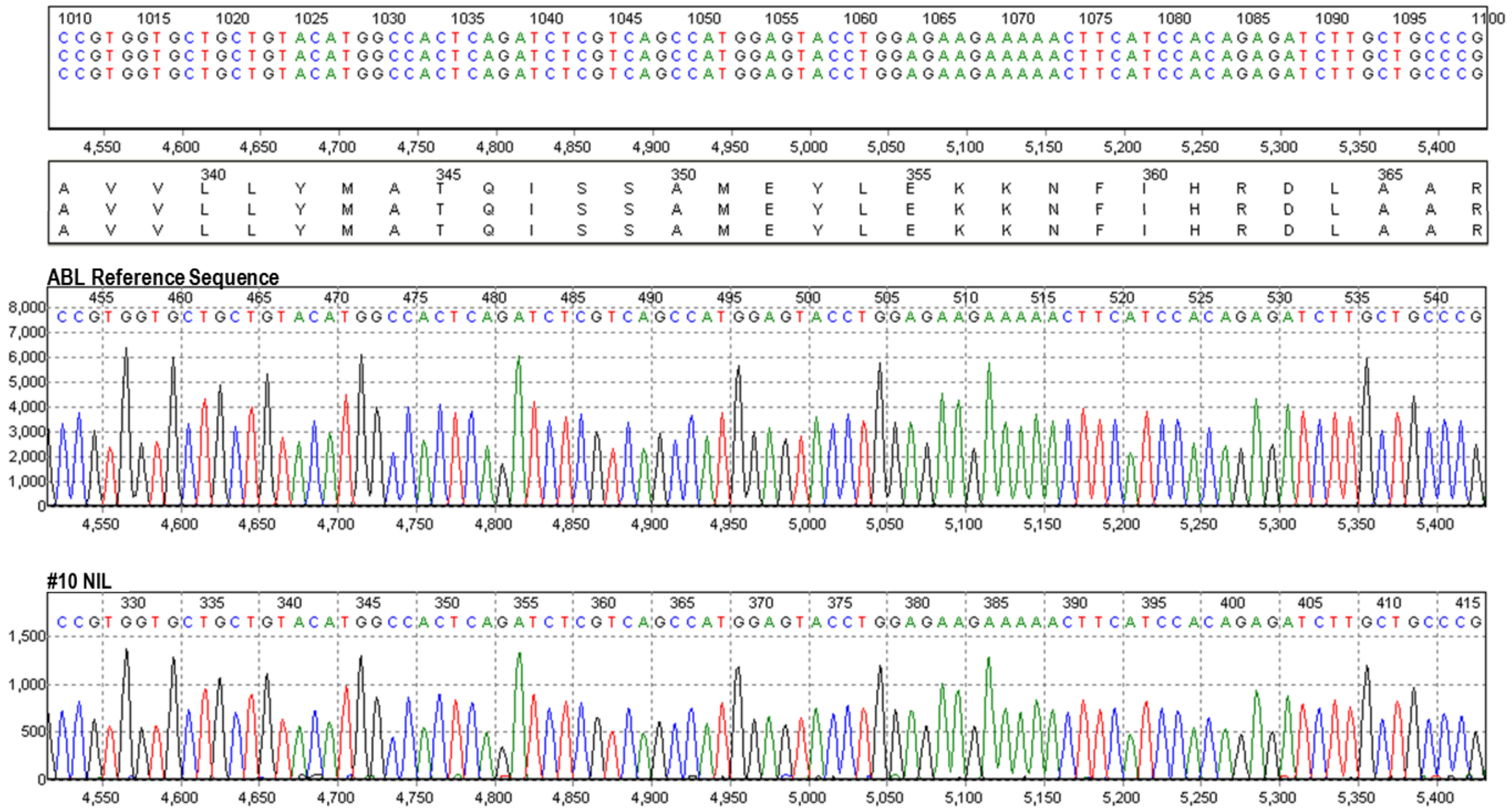
CCGTGGTGCTGCTGTACATGGCCACTCAGATCTCGTCAGCCATGGAGTACCTGGAGAAGAAAAACTTCATCCACAGAGATCTTGCTGCCCG
A V V L L Y M A T Q I S S A M E Y L E K K N F I H R D L A A R
ABL Reference Sequence
#10 NIL

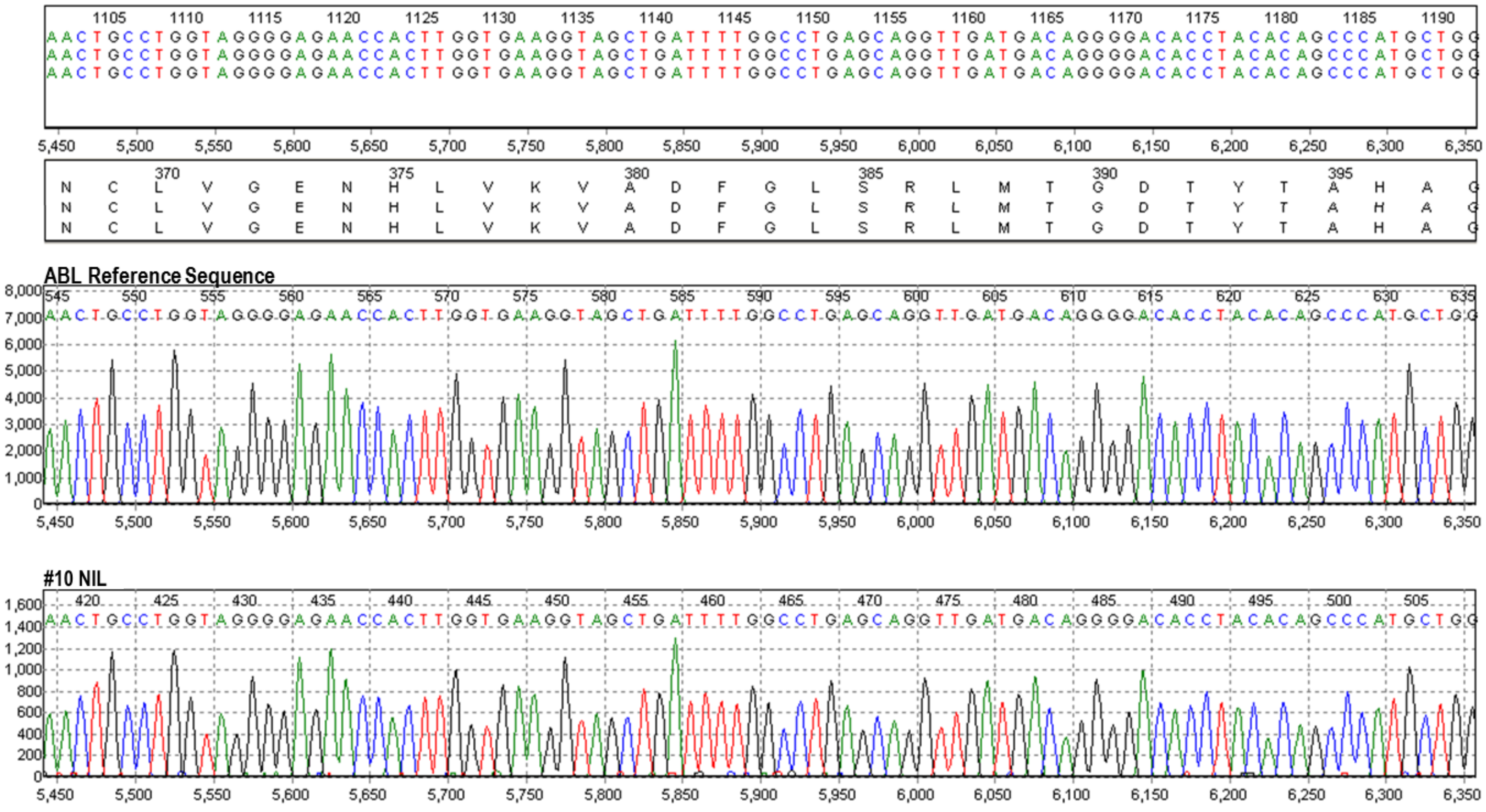

AACTGCCTGGTAGGGGAGAACCACTTGGTGAAGGTAGCTGATTTTGGCCTGAGCAGGTTGATGACAGGGGACACCTACACAGCCCATGCTGG
N C L V G E N H L V K V A D F G L S R L M T G D T Y T A H A G
ABL Reference Sequence
#10 NIL
