# Supplementary figures and images for: ABCB1 Overexpression Is a Key Initiator of Resistance to Tyrosine Kinase Inhibitors in CML Cell Lines
Source: PLoS One. 2016 Aug 18;11(8):e0161470. doi: 10.1371/journal.pone.0161470 (PMC4990177; doi:10.1371/journal.pone.0161470)

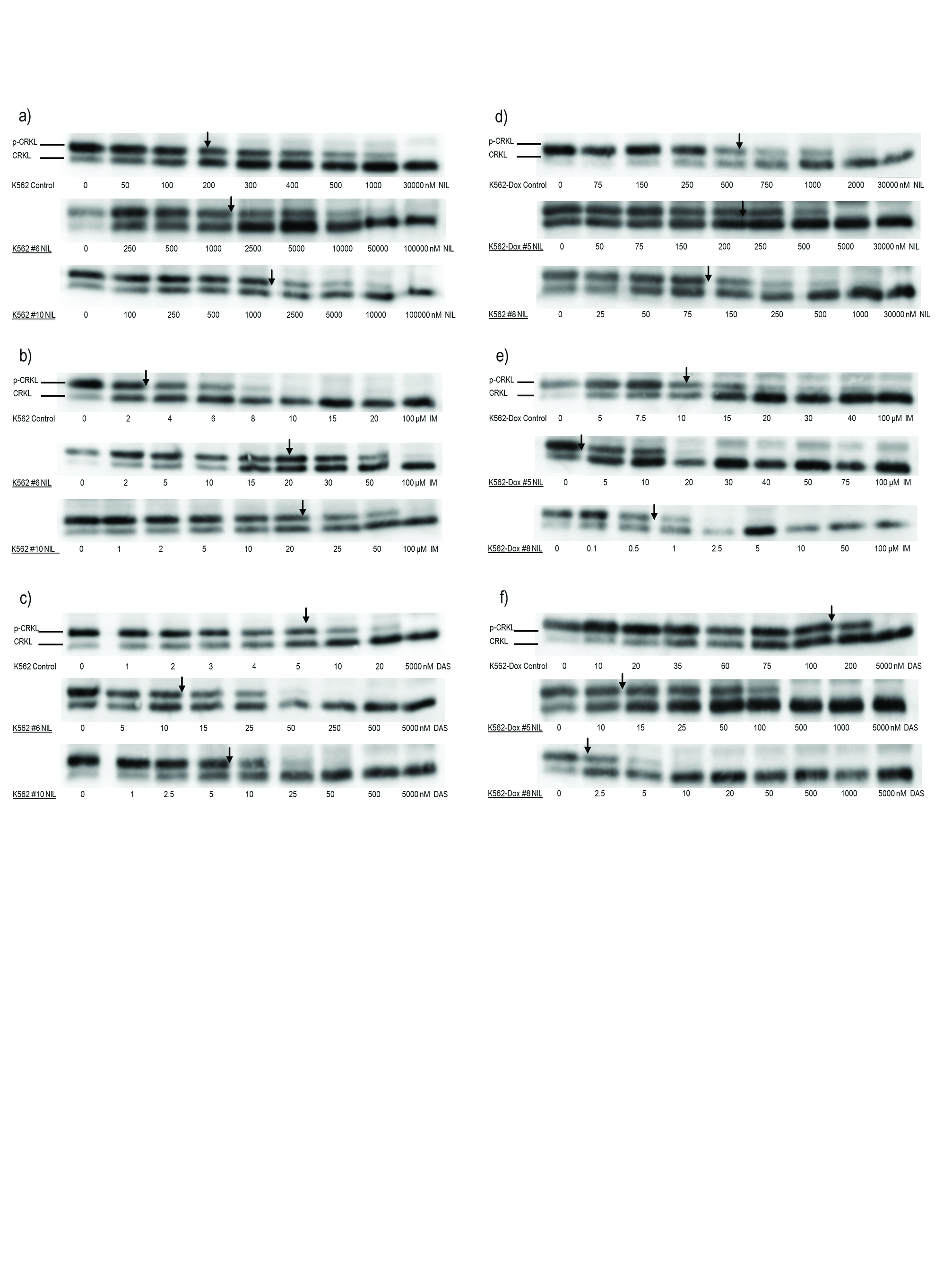

Supplement: S1 Fig — (a-c) K562 and (d-f) K562-Dox cells were incubated with the indicated concentrations of (a,d) nilotinib, (b,e) imatinib or (c,f) dasatinib. CRKL western blotting was performed to determine the concentration of TKI required for 50% BCR-ABL kinase inhibition. The western blot analyses are representative and the arrows indicate approximate IC50. NIL = nilotinib; IM = imatinib; DAS = dasatinib. (TIF) [file pone.0161470.s002.tif]

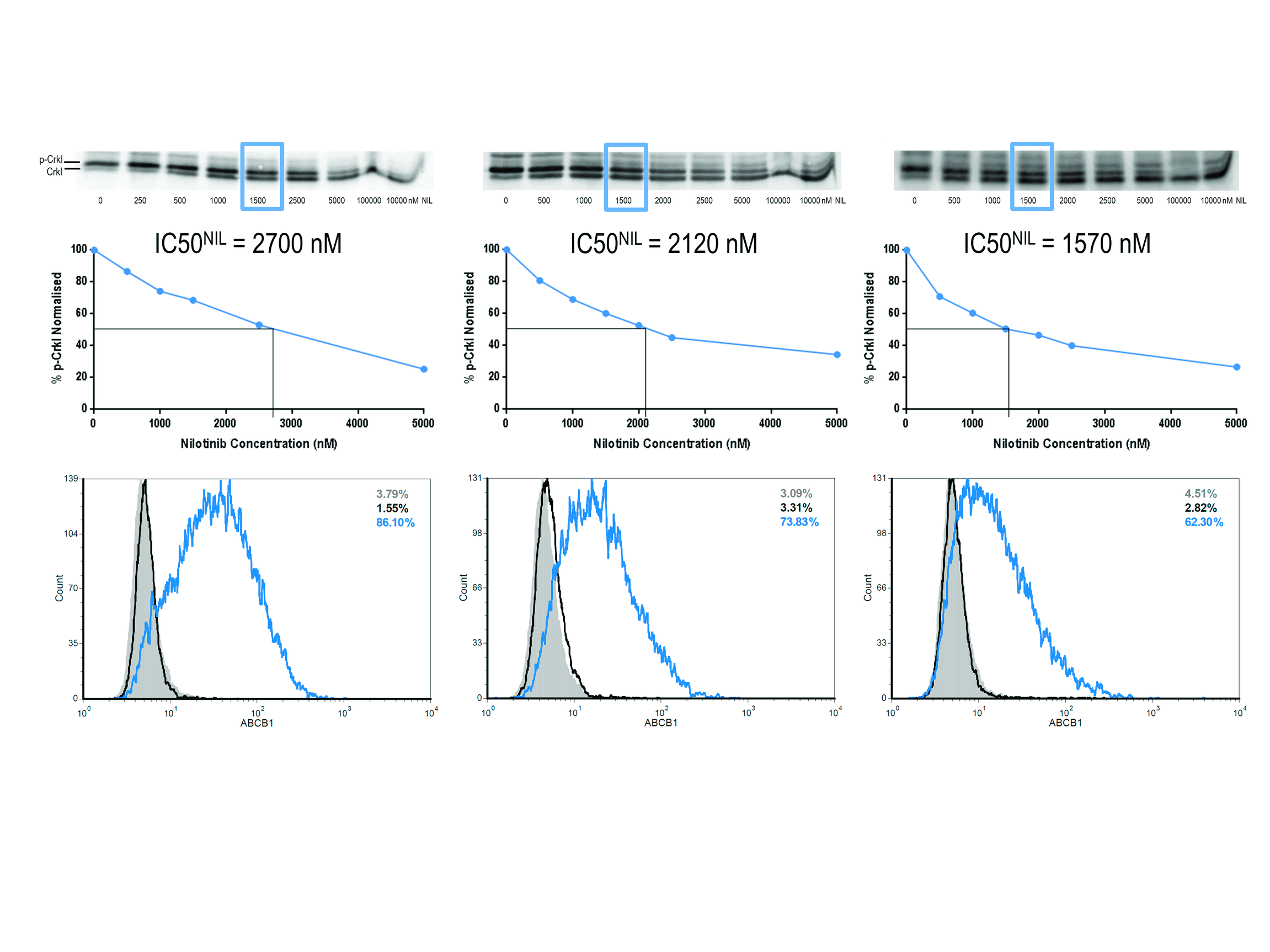

Supplement: S2 Fig — p-CRKL dependent IC50 (dose of TKI required to reduce p-CRKL levels by 50%) was determined three separate times over a period of seven days; ABCB1 expression was simultaneously determined. The western blot analyses shown represent a single experiment with the ImageQuant densitometry analyses depicted underneath. The boxes around the 1500 nM nilotinib western bands highlight the clear difference in %p-CRKL likely attributable to the level of ABCB1 expression. The percentages displayed in the histograms denote cells positive for ABCB1 expression. The bold blue and black lines represent resistant and control cells respectively, stained with ABCB1 antibody while the grey filled histograms represent cells stained with isotype control antibody. (TIF) [file pone.0161470.s003.tif]

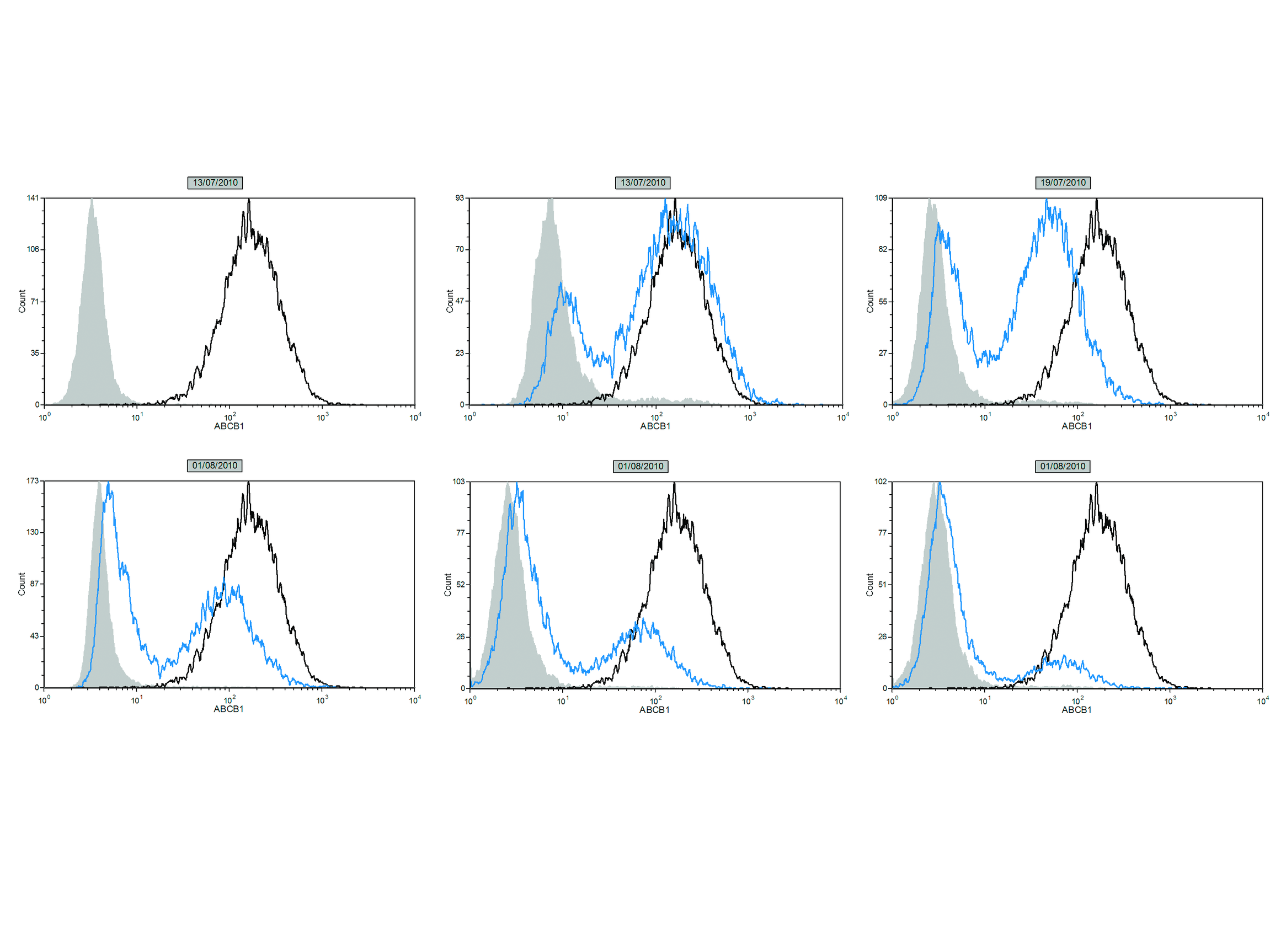

Supplement: S3 Fig — Expression levels of ABCB1 protein were assessed in K562-Dox #5 NIL cells over a period of two months compared with that in control cells. The histograms shown are representative of typical expression levels. The blue and black lines represent resistant and control cells respectively, the grey filled histograms represent cells stained with isotype control. (TIF) [file pone.0161470.s004.tif]

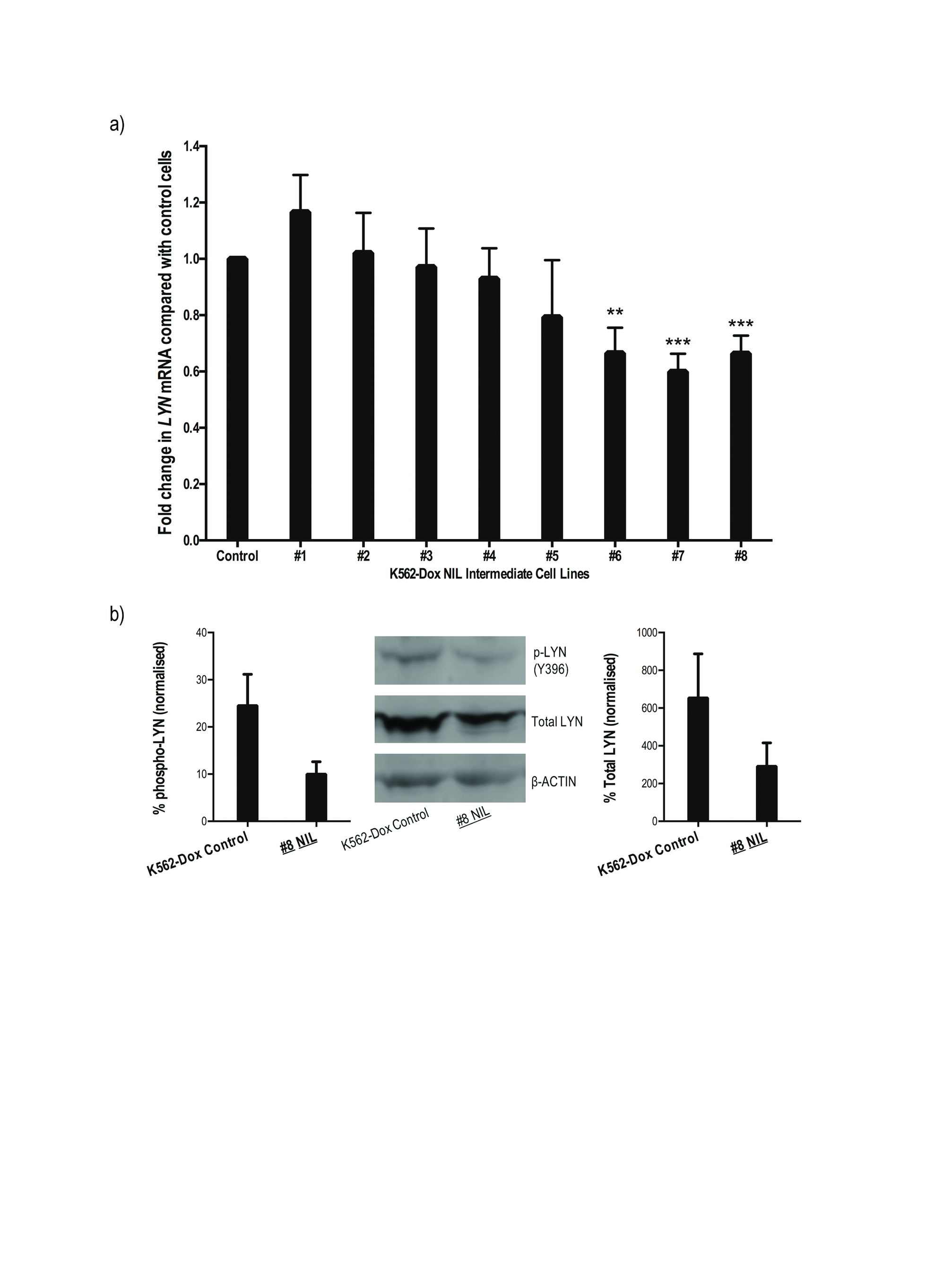

Supplement: S4 Fig — (a) mRNA and (b) protein expression levels for LYN kinase were assessed during development of nilotinib resistance in K562-Dox cells. mRNA expression represents the mean of at least three independent experiments performed in triplicate. Western blot analyses shown are representative with the corresponding quantitation representing the mean of three experiments. mRNA levels were normalised to GUSB, protein levels were normalised to β-ACTIN. Statistical analyses were performed using unpaired Student’s t-test with statistically significant p-values denoted by asterisks (** p<0.01; *** p<0.001). Error bars represent SEM. NIL = nilotinib. (TIF) [file pone.0161470.s005.tif]

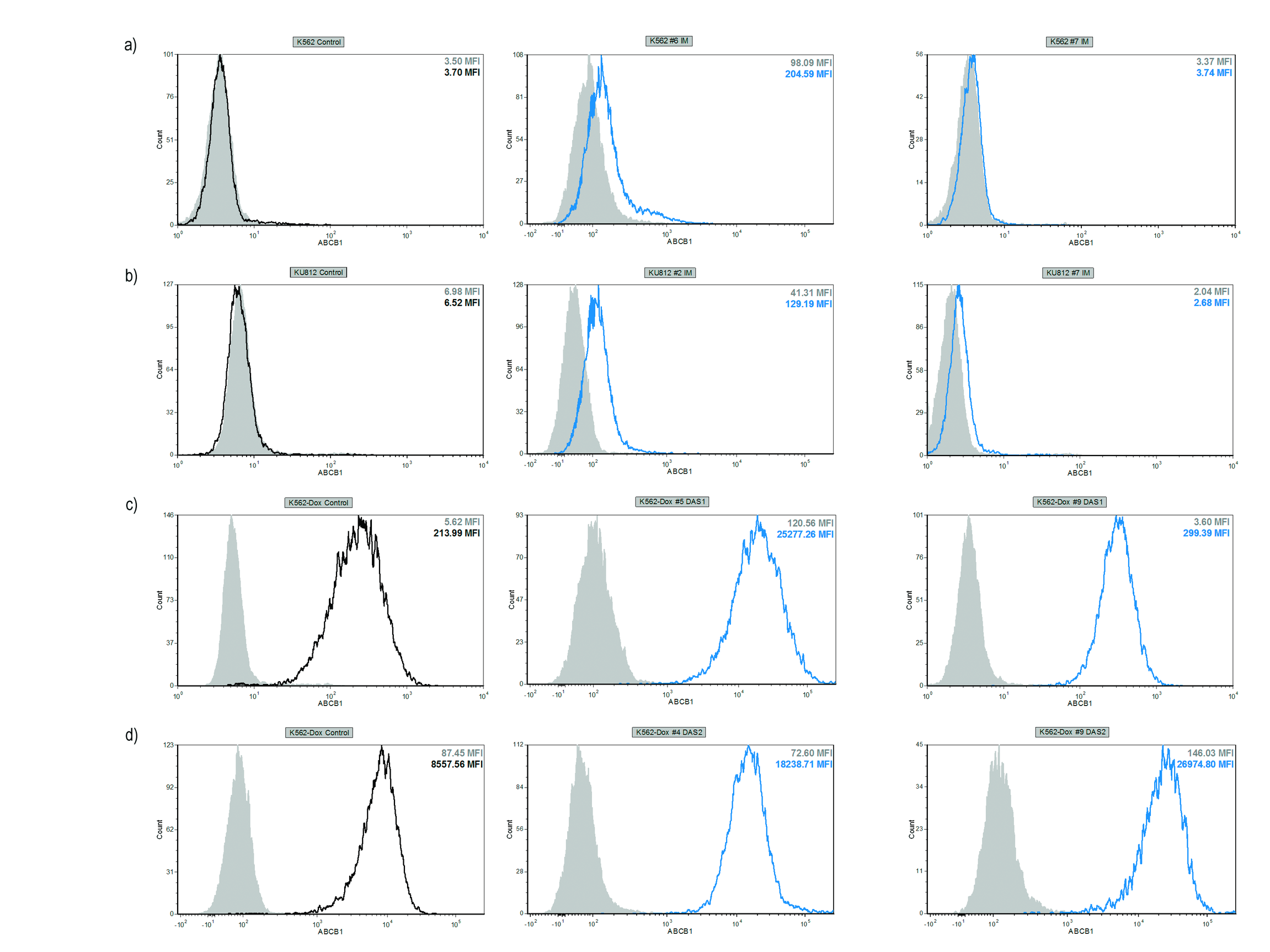

Supplement: S5 Fig — Expression levels of ABCB1 protein were assessed in (a) K562 (b) KU812 (c, d) K562-Dox cells cultured in increasing concentrations of (a,b) imatinib and (c,d) dasatinib and compared with levels in corresponding control cells. The histograms shown are representative of typical expression levels with the MFI indicated. The blue and black lines represent resistant and control cells respectively, the grey filled histograms represent cells stained with isotype control. IM = imatinib; DAS = dasatinib. (TIF) [file pone.0161470.s006.tif]

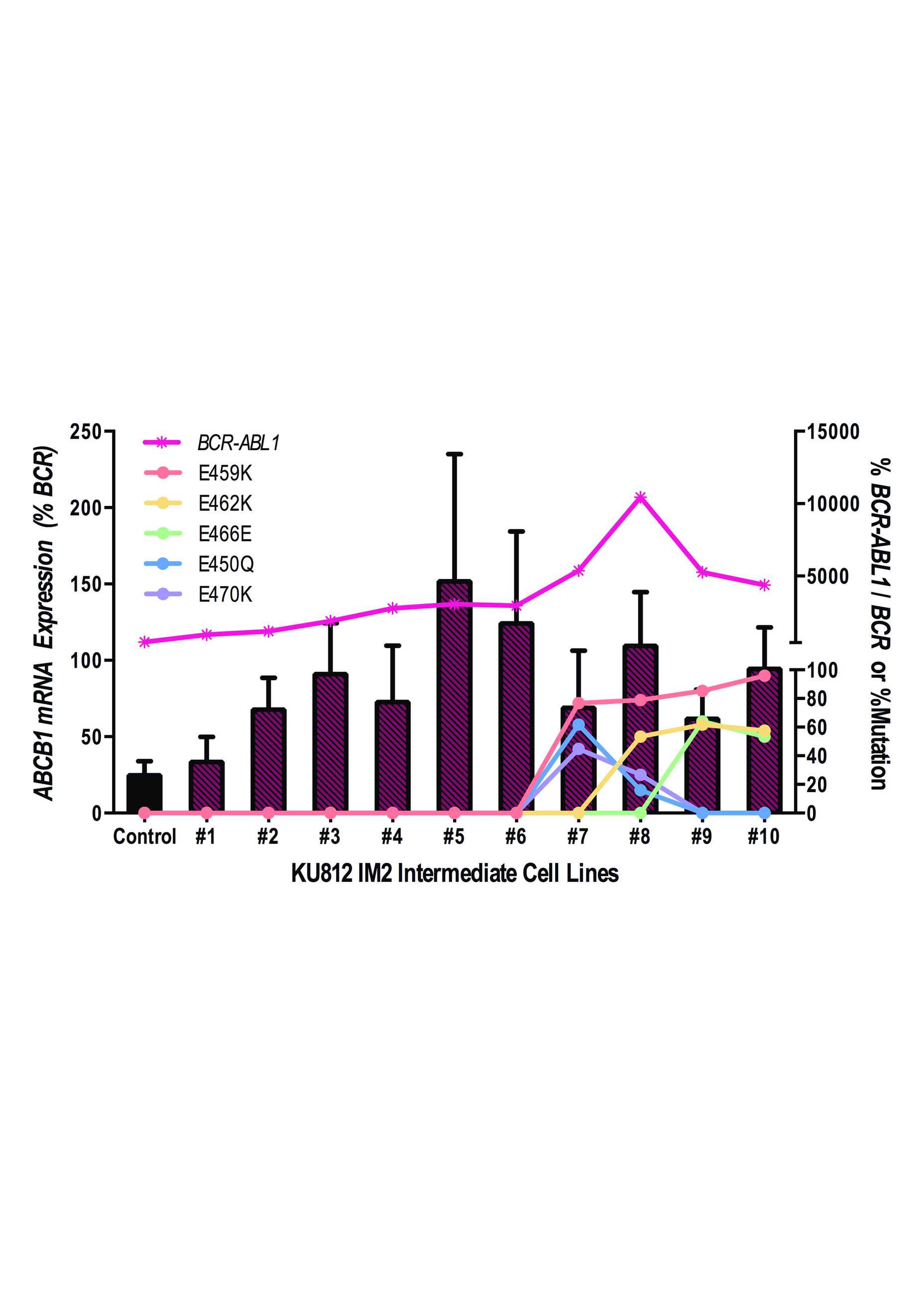

Supplement: S6 Fig — Expression levels of ABCB1 mRNA were assessed in KU812 cells resistant to imatinib. Expression levels were then correlated with other, previously defined, resistance mechanisms[15]. Specifically, % of BCR-ABL1 mRNA (maroon line) and % of various kinase domain mutations (orange, yellow, green, blue, purple lines) are indicated. mRNA expression represents the mean of at least three independent experiments performed in triplicate. Error bars represent SEM. IM = imatinib. (TIF) [file pone.0161470.s007.tif]
