# Supplementary material for: ABCB1 Overexpression Is a Key Initiator of Resistance to Tyrosine Kinase Inhibitors in CML Cell Lines
Source: PLoS One. 2016 Aug 18;11(8):e0161470. doi: 10.1371/journal.pone.0161470 (PMC4990177; doi:10.1371/journal.pone.0161470)
Supplement: S1 Table — (DOCX) [file pone.0161470.s008.docx]

**S1 Table: Summary of nilotinib (NIL) concentrations to which cell line resistance intermediates were exposed and the corresponding number of days before dose was increased**

| **K562** | | | |
| --- | --- | --- | --- |
| Nilotinib Concentration | Intermediate number | % Live Cells | Days in Culture |
| 15 nM NIL | #1 | 95.0 | 10 |
| 25 nM NIL | #2 | 99.1 | 11 |
| 50 nM NIL | #3 | 96.7 | 10 |
| 75 nM NIL | #4 | 93.7 | 14 |
| 100 nM NIL | #5 | 94.6 | 12 |
| **125 nM NIL** | **#6** | **83.6** | **28** |
| 140 nM NIL | #7 | 87.4 | 17 |
| 300 nM NIL | #8 | 93.3 | 12 |
| 400 nM NIL | #9 | 90.8 | 14 |
| 2 μM NIL | #10 | 94.1 | ∞ |

| **K562-Dox** | | | |
| --- | --- | --- | --- |
| Nilotinib Concentration | Intermediate number | % Live Cells | Days in Culture |
| 15 nM NIL | #1 | 99.6 | 11 |
| 30 nM NIL | #2 | 96.9 | 10 |
| 60 nM NIL | #3 | 98.5 | 11 |
| 100nM NIL | #4 | 91.3 | 12 |
| **125 nM NIL** | **#5** | **73.6** | **81** |
| 200 nM NIL | #6 | 76.6 | 16 |
| 1 μM NIL | #7 | 75.8 | 19 |
| 2 μM NIL | #8 | 90.7 | ∞ |
