# Supplementary material for: ABCB1 Overexpression Is a Key Initiator of Resistance to Tyrosine Kinase Inhibitors in CML Cell Lines
Source: PLoS One. 2016 Aug 18;11(8):e0161470. doi: 10.1371/journal.pone.0161470 (PMC4990177; doi:10.1371/journal.pone.0161470)
Supplement: S2 Table — Cells lines shown in bold have been assessed for ABCB1 expression in the current manuscript. Note that cell lines expressing negligible levels of ABCB1 (K562 and KU812) required longer periods of time to develop resistance to IM and DAS compared with K562-Dox cells which demonstrate overexpression of ABCB1 initially. Additionally, generation of a DAS resistant K562 cell line was extremely difficult (cells kept dying at the 1 nM DAS stage, intermediate #2) and was attempted three times before successful dose escalation occurred. A DAS resistant KU812 cell line could not be generated due to the inherent sensitivity to TKIs of this cell line. (DOCX) [file pone.0161470.s009.docx]

**S2 Table: Summary of imatinib (IM) and dasatinib (DAS) concentrations to which cell line resistance intermediates were exposed and the corresponding number of days before dose was increased**

|  | Imatinib resistance intermediate number  Days in Culture (d) | | | | | | | | | |
| --- | --- | --- | --- | --- | --- | --- | --- | --- | --- | --- |
| K562-Dox IM1 | #1 | #2 | #3 | #4 | #5 | #6 | #7 | #8 | #9 |  |
|  | 11 d | 12 d | 15 d | 12 d | 13 d | 16 d | 9 d | 13 d | ∞ |  |
|  | No mRNA/frozen cells from early intermediates (#1–#4) available for analysis  ABCB1 overexpression is a published mechanism of resistance in K562-Dox #9 IM1 cells: Tang *et al*.  Total time to terminal IM concentration: 3 months | | | | | | | | | |
| K562-Dox IM2 | #1 | #2 | #3 | #4 | #5 | #6 | #7 | #8 |  |  |
|  | 6 d | 22 d | 10 d | 45 d | 14 d | 20 d | 33 d | ∞ |  |  |
|  | ABCB1 overexpression is a published mechanism of resistance in K562-Dox #8 IM2 cells: Tang *et al*.  Total time to terminal IM concentration: 5 months | | | | | | | | | |
| K562-Dox IM3 | #1 | #2 | #3 | #4 | #5 | #6 |  |  |  |  |
|  | 20 d | 22 d | 21 d | 24 d | 21 d | ∞ |  |  |  |  |
|  | ABCB1 overexpression is a published mechanism of resistance in K562-Dox #6 IM3 cells: Tang *et al*.  Total time to terminal IM concentration: 4 months | | | | | | | | | |
| K562 IM1 | #1 | #2 | #3 | #4 | #5 | #6 | #7 | #8 | #9 | #10 |
|  | 8 d | 12 d | 11 d | 21 d | 33 d | 33 d | 25 d | 39 d | 17 d | ∞ |
|  | No mRNA/frozen cells from early intermediates (#1–#7) available for analysis  Total time to terminal IM concentration: 7.5 months | | | | | | | | | |
| **K562 IM2** | **#1** | **#2** | **#3** | **#4** | **#5** | **#6** | **#7** | **#8** |  |  |
|  | **2 d** | **24 d** | **21 d** | **16 d** | **14 d** | **37 d** | **49 d** | **∞** |  |  |
|  | **ABCB1 expression increased in response to culture with IM (Fig 4)**  **Total time to terminal IM concentration: 5 months** | | | | | | | | | |
| KU812 IM1 | #1 | #2 | #3 | #4 | #5 | #6 | #7 | #8 | #9 | #10 |
|  | 10 d | 10 d | 22 d | 11 d | 13 d | 9 d | 12 d | 17 d | 13 d | ∞ |
|  | No mRNA/frozen cells from early intermediates (#1–#6) available for analysis  Total time to terminal IM concentration: 4.5 months | | | | | | | | | |
| KU812 IM2 | #1 | #2 | #3 | #4 | #5 | #6 | #7 | #8 | #9 | #10 |
|  | 31 d | 26 d | 37 d | 51 d | 45 d | 33 d | 68 d | 32 d | 74 d | ∞ |
|  | ABCB1 expression increased with increasing IM concentration although significance was not achieved (S6 Fig)  Total time to terminal IM concentration: 13 months | | | | | | | | | |
| **KU812 IM3** | **#1** | **#2** | **#3** | **#4** | **#5** | **#6** | **#7** | **#8** |  |  |
|  | **40 d** | **37 d** | **35 d** | **15 d** | **22 d** | **19** | **27 d** | **∞** |  |  |
|  | **ABCB1 expression increased in response to culture with IM (Fig 4)**  **Total time to terminal IM concentration: 8 months** | | | | | | | | | |

|  | Dasatinib resistance intermediate number  Days in Culture (d) | | | | | | | | | | | | |
| --- | --- | --- | --- | --- | --- | --- | --- | --- | --- | --- | --- | --- | --- |
| **K562-Dox DAS1** | **#1** | **#2** | **#3** | **#4** | **#5** | **#6** | **#7** | **#8** | **#9** | **#10** |  |  |  |
|  | **18 d** | **73 d** | **21 d** | **30 d** | **35 d** | **19 d** | **32 d** | **14 d** | **13 d** | **∞** |  |  |  |
|  | **ABCB1 expression increased in response to culture with DAS (Fig 4)**  **Total time to terminal DAS concentration: 6.5 months** | | | | | | | | | | | | |
| **K562-Dox DAS2** | **#1** | **#2** | **#3** | **#4** | **#5** | **#6** | **#7** | **#8** | **#9** |  |  |  |  |
|  | **24 d** | **69 d** | **7 d** | **9 d** | **31 d** | **11 d** | **21 d** | **31 d** | **∞** |  |  |  |  |
|  | **ABCB1 expression increased in response to culture with DAS (Fig 4)**  **Total time to terminal DAS concentration: 8 months** | | | | | | | | | | | | |
| K562 DAS1 | **#1** | **#2** | **#3** | **#4** | **#5** | **#6** | **#7** | **#8** | **#9** | **#10** | **#11** | **#12** | **#13** |
|  | 38 d | 32 d | 39 d | 70 d | 21 d | 21 d | 42 d | 18 d | 13 d | 11 d | 17 d | 16 d | ∞ |
|  | ABCB1 expression was not evaluated in this cell line  Total time to terminal DAS concentration: 11 months | | | | | | | | | | | | |

Cells lines shown in bold have been assessed for ABCB1 expression in the current manuscript. Note that cell lines expressing negligible levels of ABCB1 (K562 and KU812) required longer periods of time to develop resistance to IM and DAS compared with K562-Dox cells which demonstrate overexpression of ABCB1 initially. Additionally, generation of a DAS resistant K562 cell line was extremely difficult (cells kept dying at the 1 nM DAS stage, intermediate #2) and was attempted three times before successful dose escalation occurred. A DAS resistant KU812 cell line could not be generated due to the inherent sensitivity to TKIs of this cell line.
